# Determinants of Performance of Health Systems Concerning Maternal and Child Health: A Global Approach

**DOI:** 10.1371/journal.pone.0120747

**Published:** 2015-03-30

**Authors:** Carlos Eduardo Pinzón-Flórez, Julián Alfredo Fernández-Niño, Myriam Ruiz-Rodríguez, Álvaro J. Idrovo, Abel Armando Arredondo López

**Affiliations:** 1 Centre for Research in Health Systems (CISS), Instituto Nacional de Salud Pública de México, Cuernavaca, Mexico; 2 Information Center for Public Health Decisions (CENIDSP), Instituto Nacional de Salud Pública, Cuernavaca, Mexico; 3 Department of Public Health, School of Medicine, School of Health, Universidad Industrial de Santander, Bucaramanga, Colombia; University Hospital Basel, SWITZERLAND

## Abstract

**Aims:**

To assess the association of social determinants on the performance of health systems around the world.

**Methods:**

A transnational ecological study was conducted with an observation level focused on the country. In order to research on the strength of the association between the annual maternal and child mortality in 154 countries and social determinants: corruption, democratization, income inequality and cultural fragmentation, we used a mixed linear regression model for repeated measures with random intercepts and a conglomerate-based geographical analysis, between 2000 and 2010.

**Results:**

Health determinants with a significant association on child mortality(<1year): higher access to water (βa Quartile 4(Q4) vs Quartile 1(Q1) = -6,14; 95%CI: -11,63 to -0,73), sanitation systems, (Q4 vs Q1 = -25,58; 95%CI: -31,91 to -19,25), % measles vaccination coverage (Q4 vs Q1 = -7.35; 95%CI: -10,18 to -4,52), % of births attended by a healthcare professional (Q4 vs Q1 = -7,91; 95%CI: -11,36 to -4,52) and a % of the total health expenditure (Q3 vs Q1 = -2,85; 95%CI: -4,93 to -0,7). Ethnic fragmentation (Q4 vs Q1 = 9,93; 95%CI: -0.03 to 19.89) had a marginal effect. For child mortality<5 years, an association was found for these variables and democratization (not free vs free = 11,23; 95%CI: -0,82 to 23,29), out-of-pocket expenditure (Q1 vs Q4 = 17,71; 95%CI: 5,86 to 29,56). For MMR (Maternal mortality ratio), % of access to water for all the quartiles, % of access to sanitation systems, (Q3 vs Q1 = -171,15; 95%CI: -281,29 to -61), birth attention by a healthcare professional (Q4 vs Q1 = -231,23; 95%CI: -349,32 to -113,15), and having corrupt government (Q3 vs Q1 = 83,05; 95%CI: 33,10 to 133).

**Conclusions:**

Improving access to water and sanitation systems, decreasing corruption in the health sector must become priorities in health systems. The ethno-linguistic cultural fragmentation and the detriment of democracy turn out to be two factors related to health results.

## Introduction

A widespread definition for the functioning of a health system is the one proposed by the World Health Organization (WHO), “the organized social response,” whose main goal is to promote, restore, or maintain health [1]. In 2007, with the purpose of promoting a common understanding about what a health system is and identifying action areas for the strengthening of health systems, the WHO prepared a framework made up of six building blocks, as follows: 1) health service coverage, 2) health human resources, 3) health information systems, 4) medical products, vaccines and health technologies, 5) health financing, and 6) leadership and governance [2]. The goal of these blocks is to support a health system that intends to prevent, treat, and control diseases as well as maintain the physical and mental welfare of all the persons, in an equal and efficient manner, within a specific geographical area [3].

The activities of the health system range from the direct rendering of the services through clinics and hospitals, to prevention strategies at the community level and education for health [3]. There has been a renewed interest during the last decade concerning the horizontal model of the health systems as per the promotion and maintenance of health [3]. In addition, work has been carried out concerning the strengthening of a public health system based on regional processes developed by the Mesoamerican Public Health Institute, Mercosur, and international processes in order to attain the Millennium Development Goals (MDG) [4,5]. Nevertheless, the indicators to assess the strengthening of health systems and the possible determinants related to that functioning have been analyzed less; therefore, they are less understood.

There is an ongoing debate on the global health “geometry” of the vertical and horizontal approaches for health care since both have advantages and limitations [6–8]. Both systems, private and public, may use vertical or horizontal approaches concerning health care; some using the term ‘diagonal’ along with them to describe the combination of the two approaches to optimize the processes and the results [9]. A notable trend is that the private organizations tend to have a more limited approach and use a more vertical one. For example, in many low-income countries (LIC), donors and projects promoted by external entities have had some success, particularly with the creation of health centers for HIV/AIDS treatment and prevention, immunization coverage, tuberculosis control, as well as to achieve the regression of malaria through prevention campaigns and educational measures: these activities and programs have been deemed a vertical approach for health [7,8].

In general, these initiatives focused on illnesses include three characteristics: 1) temporarily intense, as they are financed for a short period with specific interventions, 2) they may avoid the bureaucracy of the public sector since the money for their financing, and the purchase and procurement processes for them are carried out within an own time framework of the initiative thus avoiding the inefficiency of the health public sector and its actors, and 3) they may be implemented within any given process or context since they are described interventions that meet the own requirements of the financial backers (as vaccines through the GAVI alliance, the Global Alliance for Vaccines and Immunization). Nonetheless, the investments for the resources and functioning of a health system (for example, the horizontal approach to health) are based on the assumption that the adequate functioning depends on the efficiency of the health system in terms of the health improvement of the population [10]. The initiatives towards cross-sectional models is strengthened by the implementation of health promotion and prevention processes, including comprehensive primary care strategies which allow managing the risk of getting ill by handling the risk conditions of the individuals [11]. Some countries have had satisfactory isolated experiences as per reducing the risk conditions for non-communicable chronic diseases, child health and maternal health, with some obstacles to the implementation regarding the financing of the system, geographical and political limitations [12].

Although some countries have been able to substantially improve mortality rates of breastfed babies (IMR<1 year), children (IMR<5 years) and maternal mortality rates (MMRs) during the last century, improvements have decreased and have become less fast although some countries have even evidenced an increasing tendency [13]. It is calculated that around 9.7 million children under five years of age die in the world every year [14] and there were 529,000 maternal deaths for year 2000 [15]. In addition, mortality rates are quite variable among countries. Health inequalities, as well as social and environmental determinants may explain the behavior of mortality rates in these countries; likewise, the performance of the health systems of said nations [16].

The differences in the general mortality rates among nations may, in part, be explained by the functioning and ability of a health system to protect health beyond the specific approach of the disease [17]. Important financing entities, such as the U.S. Global Health Initiative have generated financial contributions to strengthen health systems at the expense of initiatives focused on the disease, despite the fact that the health policies have recommended to redirect said efforts towards the intervention of the healthy population and the development of valid indicators to determine and supervise the performance of the processes and functions of the health systems [18].

An element that has placed the problem of inequalities at the forefront is the persistence of the health gradient: the verification that health conditions are not only different among the poorest groups and the rest of society but also that health, in all societies, and above all the prevalence of all the chronic and acute diseases have the same tendency of the social structure; that is, the prevalence of almost all the diseases and health problems increases by going down one step of the social ladder [19, 20].

Social determinants, such as poverty, violence, migration, gender, and ethnic inequality have been explored as social determinants of health evidencing their effect from the individualistic model of health and the quality of the response of the health system in terms of the rendering of the services and, definitely, in relation to the impact on the health conditions [21]. However, there is no evidence of an aggregate analysis that includes the four social determinants proposed in this study; the association with health system performance and health status at the country and region levels. Therefore, the aim of this study was to develop an exploratory analysis to study the strength of the association among relevant health results at the country level (IMR<1 year, IMR<5 years, MMR, the performance of the health system, and four (4) social determinants: cultural fragmentation, corruption, social capital, democratization, and income inequality at an ecological level [22, 23].

## Materials and Methods

### Ethics statement

The sample for our work was derived from the World Bank and World Health Organization Database. These databases are public record and are available on internet portals and the offices of these organizations. This survey was approved by the Ethics and Research Committees of the National Institute of Public Health.

An ecological study was conducted with 154 countries (S1 Table).

### Data and variables

#### Data source

All the data were obtained from free-access databases of national and international official entities; some indicators evidenced few data reported during some time periods assessed so their use was done with caution. Data reported by the World Bank’s observatory were used concerning resulting variables, IMR<1 year, IMR<5 years, and MMR. The group of explanatory variables used were the indicators mentioned in Table 1 for each block of the functions of the health systems, and three (3) of the four (4) health determinants selected, except for the cultural fragmentation index, at the country level and for the period between 2000 and 2010 recommended by the WHO [1]. The financing data, in terms of health expenditure, were obtained from the WHO Statistical Information System (WHOSIS) and they are standardized through the international exchange rate of the dollar of the United States, which is a common monetary unit that takes into account the differences in the annual average of the relative purchasing power. The following were the indicators of the four (4) social determinants in this analysis: concerning corruption, the Corruption Perceptions Index, an indicator designed to measure levels of corruption perception of the public sector published every year by Transparency International [24]; this indicator was also used as a proxy of the assessment of the management function which includes three key factors: "The establishment, application, and follow-up of rules for the health system; ensuring equality of conditions for all the agents of the system; and, the definition of the strategic agents for the health system as a whole.” Currently, there is no indicator to measure health management at the national level; consequently, the corruption index may indirectly assess the government’s management and, therefore, the regulation of the health system [25].

**Table 1:** Indicators selected per function of the health system.

Function block of the health system	Indicator / variable
Health services coverage	Measles and DPT vaccination coverage, % of the population with sustainable access to fresh water and sanitation, % of births attended by qualified personnel, % of women with prenatal control (one visit), % of women with prenatal control (four visits).
Health human resources	Density of physicians per 1,000 inhabitants, density of beds per 1,000 inhabitants.
Health financing	Health out-of-pocket expenditure, health expenditure per capita, health expenditure: % of the GDP, public health expenditure.
Leadership and governance	Corruption index.

Since corruption depends on the cultural context [26] and it is related with government functioning [27], poverty and types of enterprises in the national economies [28], we used the 2000 Alessina et al. index was used regarding cultural fragmentation. It is a proxy for cultural similarities and it includes the definition of ethno-linguistic fragmentation as per the probability that when randomly selecting two individuals of a country they may belong to two different ethnic groups [29]. Alessina differentiates said heterogeneity in three dimensions: ethnical, linguistic, and religious. Thus, a higher fragmentation index indicates a higher fragmentation of the country. Cultural fragmentation has been used in previous studies exploring associations between income inequality and population health indicators, because ethnic heterogeneity in health models may bias the associations [30,31].

The Freedom House’s index was used concerning democratization; this index assesses the countries as per their political rights and civil liberties which mostly derive from the Universal Declaration of Human Rights [32]. The countries are categorized as free, partially free, or not free. Finally, the Gini coefficient was used regarding income equality; this coefficient assesses the income inequality of the countries [33].

The selection of the variables was made before hand for each block of the functions of the health systems according to the defined framework of the WHO [1]. The goal was to select the variables (indicators) that were more sensitive as per the assessment of the block and, therefore, representing it. This was attained through a systematic search of the indicators per block. The information of these indicators was subsequently extracted from international and national sources. These indicators were studied under the Pearson or Spearman correlation analysis as per main components depending on their behavior and analysis. The indicators that had correlation indices between 0.4 and 0.7, and a > 20 Eigenvalue were selected.

A block of a health system is the construction of information systems that may be captured by the presence of an operating surveillance system; nevertheless, this indicator was not available for this multinational data construction block. Together, these indicators act as a proxy representing the strength of the health systems as per the financing, the personnel, and the rendering of the health services to their citizens [34]. Demographic variables such as the fertility rate, the growth of the national population, the growth of the urban population, and the participation of the female labor force in order to assess the demographic transition and economic development variables such as the GDP were analyzed as covariables. This with the purpose of being able to explain heterogeneity among countries. These data were also obtained by the World Bank’s observatory for all the observation periods.

From the 217 countries reported by the World Bank, 154 countries provided enough data for the variables selected. Eight of the 154 countries would have been excluded due to the absence of data concerning access to water. The assumption of a 95% value was carried out for Poland and Portugal instead of excluding them because of the lack of data; we assumed 100% in Belgium, France, Ireland, Italy, New Zealand, and the United Kingdom (the average value of Australia, the countries of Western Europe, and North America). This region would have a low level of representation without this assumption of Western and Southern European countries.

### Statistical analysis

The qualitative variables were summarized with proportions along with their respective 95% confidence intervals. On the other hand, the median, the range, and the interquartile range were used in order to describe the quantitative variables since the distribution of most of the indicators was markedly asymmetric and there were extreme values. In addition, the distributions were explained through histograms and “q-q plots.” The relation between each response variable (IMR< 1year, IMR<5 years, MMR) and each independent variable (described in the previous section) was explored with Kruskal-Wallis tests for the bivariate analysis.

A mixed linear models was used for the multivariate analysis for repeated measures with a country random intercept [35] and a geographically weighted regression models for the graphic assessment of the associations. We use three mixed lineal models, the first type of model allows explaining the variance of the annual rates (IMR <1 year, IMR <5 years, and MMR) taking into consideration their belonging to the same country. Two models were devised for the three scenarios: the first one with the highest possible number of observations, and the second one with the highest possible number of explanatory variables. This, due to the availability of the information for the countries as per all the variables. For IMR <1 year, model 1 included the following independent variables: % of access to fresh water, % of access to sanitation services, % of measles vaccination coverage, health expenditure per capita as a percentage of the overall health expenditure, % of primary education in women and ethno-linguistic cultural fragmentation. The variables were the same for model 2, adding the number of physicians per 1,000 inhabitants and the Gini coefficient. For IMR<5 years, model 1 included the percentage of access to fresh water, % of coverage of measles vaccination, health expenditure per capita. Model 2 added to the analysis the percentage of access to sanitation systems, the coverage for the first prenatal control visit, DPT vaccination coverage, number of physicians per 1,000 inhabitants, health out-of-pocket expenditure, religious cultural fragmentation, the Gini coefficient, and % of women with a job. As per MMR, model 1 included: % of access to fresh water, % of access to sanitation services, % of births attended by healthcare professionals, and fertility rate. Model 2 also included the number of physicians per 1,000 inhabitants, total health expenditure, and the corruption index. All the models were adjusted due to the effect of time as a qualitative variable. Random intercepts models were compared with models of fixed effects for the difference in the intra-country measured with mean intra-country (fixed effects model for intra-country variability). Using the Hausman test no statistically significant systematic differences in the estimated coefficients are found by comparing these fixed models with random intercepts models (p> 0.10). Therefore, to maintain statistical consistency and not to find a correlation between the independent variables and the random component, we chose random patterns, which are known to be more efficient (have lower variance estimators) and also other to assess the association of variables fixed in time.

The geospatial regression models included the same variables of the mixed regression model with repeated measures; the models were adjusted in terms of the variables that assessed the functions of the health systems. The level of association was established through quartiles, and the unit of observation was the country and the geographical region. The exploratory analysis of spatial data in terms of spatial patterns is applied first. The spatial autocorrelation and non-seasonality were calculated as a starting point in the variables by using the Moran index estimator for the global effect and the local G for the regional effect. The Moran index [36] is a widely used global spatial autocorrelation measure which verifies if there are any relations between the location and attribute values. A positive significant statistics indicates that other values of similar attributes are spatially grouped instead of being randomly distributed. In contrast, a negative significant statistics shows different values in close localities which evidence a more disperse pattern. Getis and Ord [37] have introduced the statistical G to detect groups with high or low values according to their location. High positive values refer to “hot spots” and high negative values refer to “cold spots.” "Hot spots" may be described as areas in which countries with high levels are surrounded by other countries with high levels. In contrast, "cold spots" are groups with low-level countries surrounded by other low-level countries. A crucial step in spatial modeling is the selection of an adequate representation of the space.

The assumptions of the models were verified and calculations were made concerning their coefficient of determination (R2). In addition, the models were compared through the Akaike Information Criteria (AIC) for the mixed linear regression models for repeated measures and the Bayesian Information Criterion (BIC) for both models [38].The existence of random slopes was assessed but the variance of the slopes was not significant; therefore, we used mixed effect models only with random intercepts. All the analyses were completed with the STATA 12 statistical program and ArcGIS.

## Results and Discussion

**Fig 1:**
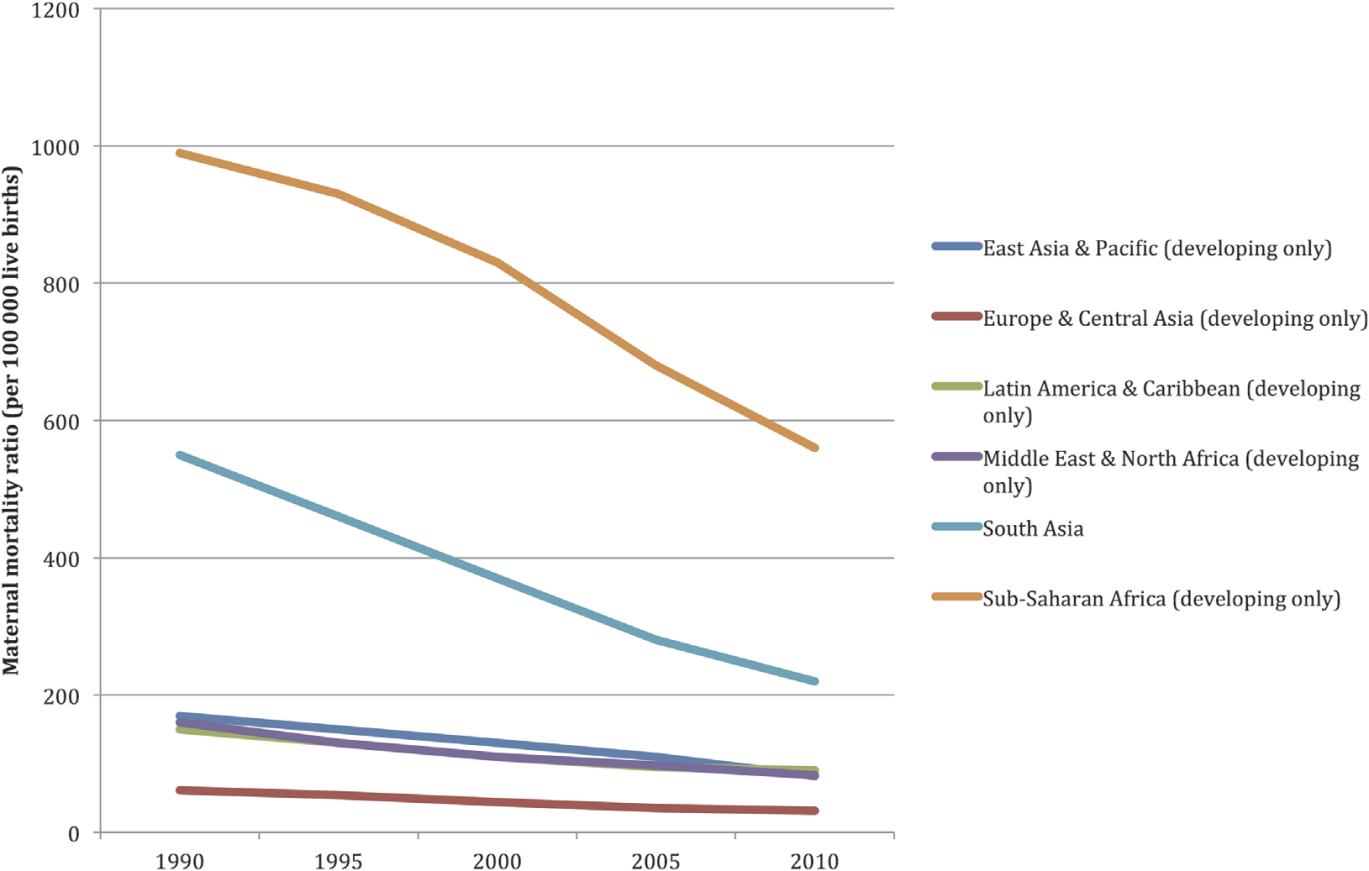
Maternal mortality rate (MMR) tendency: 1990 to 2010. Aggregation unit: country. World Bank

**Fig 2:**
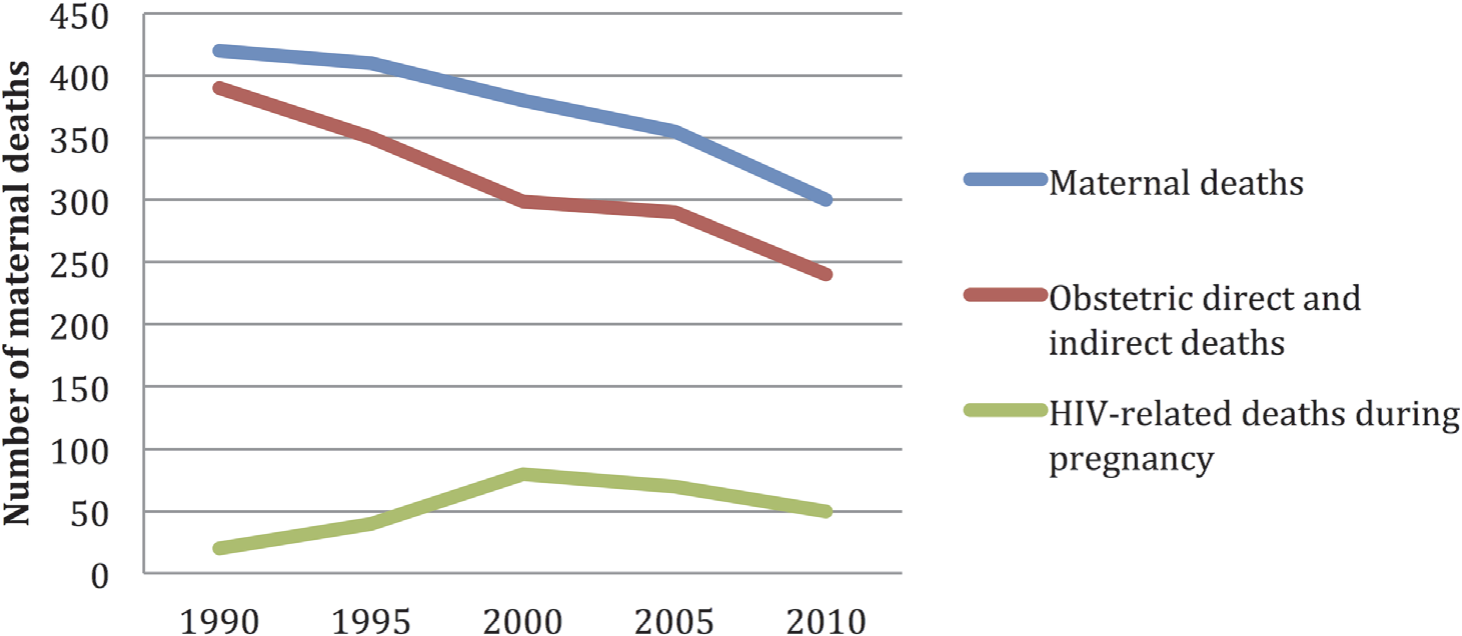
Number of maternal deaths due to a specific cause of death: 1990 to 2010. Aggregation unit: country. World Bank

**Fig 3:**
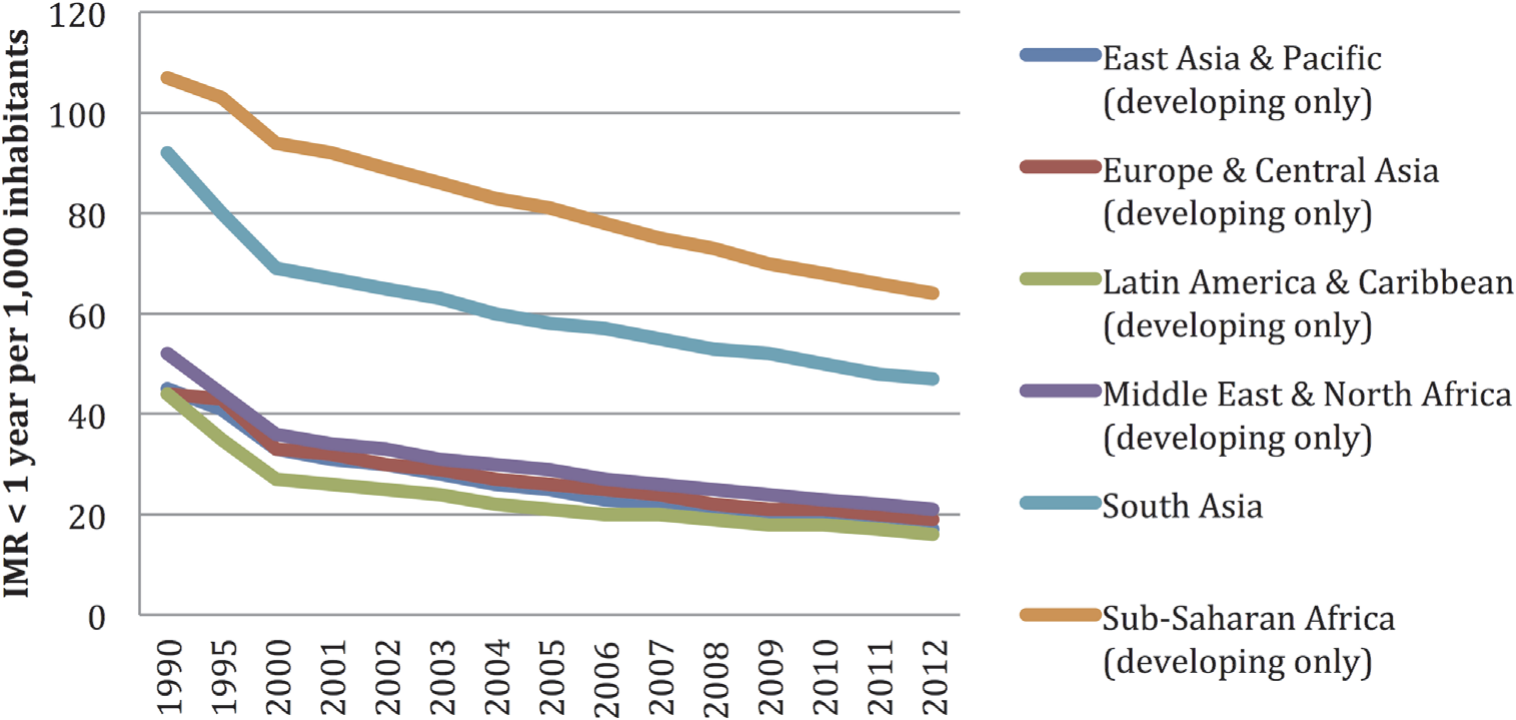
Infant mortality rate (IMR) <1 years tendency: 1990 to 2010. Aggregation unit: country. World Bank

**Fig 4:**
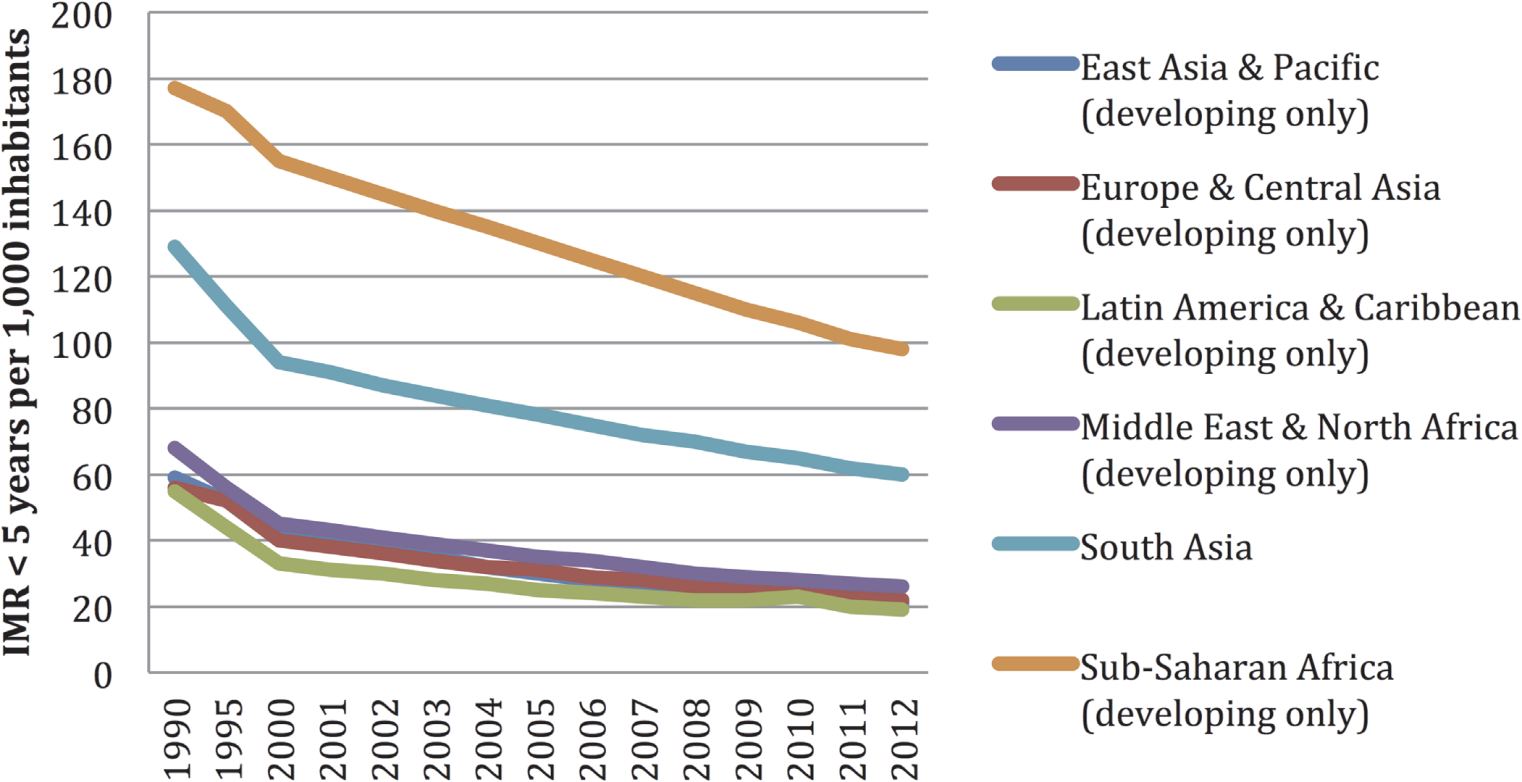
Infant mortality rate (IMR) <5 years tendency: 1990 to 2010. Aggregation unit: country. World Bank

The descriptive statistical data for each one of the outcome variables (IMR< 1 year, IMR <5 years, and the MMR) and the explanatory variables are included in S2 Table. The IMR< 1 year, IMR<5 years, and the MMR around the world, evidenced a descending trend during the periods included (2000–2010): from 48.8 deaths per 1,000 live births/year to 28.2 deaths per 1,000 live births/year, from 71.3 deaths per 1,000 live births/year to 39.37 deaths per 1,000 live births, and from 333.31 deaths per 1,000 live births to 178.99 deaths per 1,000 live births, respectively. Africa is the region with the worst figures as per child and maternal mortality; it behaves in a differential manner throughout the analysis periods (see Figs 1–4).

From the 217 countries reported by the World Bank, 54 (24.8%) are in Africa, 51 (23.5%) in Asia; 49 (22.5%) in Europe; 42 (19.35%) in Latin America and the Caribbean, 2 (0.92%) in North America, and 19 (8.76%) in Oceania. The proportion of countries included in the model varies among regions, where 90% of all the African countries are included, 73.5% from Europe, 72.4% from Asia, 100% from North America, 50% from Latin America, but only 47% from Oceania had enough data in order to be included in this model (Table 2). The countries included in the analysis and the mortality rates are depicted in Fig 1; Figs 2, 3, and 4 depict the world distribution of mortality rates from 2000 to 2010. A comparative analysis was conducted between years 2000 and 2010; nevertheless, the explanatory variables did not evidence variations above 10% as per the years assessed.

**Table 2:** Description of the classification of the countries per region (n = 154 countries).

**Region**	**Total countries identified**		**Total number of countries per region, % included in the analysis per region**	
	**N**	**%**	**n**	**%**
Africa	54	24.88	49	90.74
Asia	51	23.50	37	72.55
Europe	49	22.58	36	73.47
Latin America and the Caribbean	42	19.35	21	50.00
North America	2	0.92	2	100
Oceania	19	8.76	9	47.37
Total	217		154	70.97

All the selected indicators of the health system were significantly associated to the infant mortality rate, infant mortality rate < 1 year, and the maternal mortality ratio in the bivariate analysis (S3 Table). The country, region, year, and other variables of the model were controlled in the multivariate analysis. The determinants of the health system with a significant association in terms of infant mortality < 1 year were identified: higher access to water for quartiles 2 to 4 in relation to quartile 1 (**β**a Quartile 4 (Q4) vs Quartile 1 (Q1) = -6.14; 95% CI: -11.63 to -0.73), and sanitation systems for quartiles 2 to 4 in comparison to quartile (**β**a Q4 vs Q1 = -25,58; 95% CI: -31.91 to -19.25); likewise, for the % of measles vaccination coverage (**β**a Q4 vs Q1 = -7.35; 95% CI: -10.18 to -4.52); % of births attended by healthcare professionals for quartiles Q2 and Q4 (**β**a Q2 vs Q1 = -2,10; 95% CI: -3.91 to -0.3) and (**β**a Q4 vs Q1 = -7,91; 95% CI: -11.36 to -4.52) and public health expenditure as a % of the overall health expenditure for quartiles 2 to 4 in comparison with quartile 1 (**β**a Q3 vs Q1 = -2,85; 95% CI: -4.93 to -0.7). A higher cultural-ethnical fragmentation between Q1 vs Q4 (**β**a Q4 vs Q1 = 9,93; 95% CI: -0.03 to 19.89) and higher cultural-linguistic fragmentation between Q1 vs Q4 (**β**a Q4 vs Q1 = 8,80; 95% CI: -1 to 18.6), have an association of risk, although this is not statistically significant (Table 3).

**Table 3:** Multivariate analysis for IMR < 1 years.

Function of the health system	Variable	Categories	Model 1 (n = 112 countries)				Model 2 (n = 60 countries)			
			Beta	95% CI		P	Beta	95% CI		p
Coverage of health services	% of access to fresh water	Q1	Reference				Reference			
	% of access to fresh water	Q2	-8.63	-12.61	-4.64	<0.01	-26.28	-33.63	-18.93	<0.01
	% of access to fresh water	Q3	-8.68	-13.10	-4.26	<0.01	-25.69	-33.28	-18.09	<0.01
	% of access to fresh water	Q4	-6.18	-11.64	-0.73	0.026	-30.31	-42.87	-17.75	<0.01
	% of access to sanitation systems	Q1	Reference				Reference			
	% of access to sanitation systems	Q2	-11.91	-16.12	-7.69	<0.01	-6.94	-15.54	1.65	0.113
	% of access to sanitation systems	Q3	-19.16	-24.58	-13.74	<0.01	-13.12	-23.03	-3.20	0.01
	% of access to sanitation systems	Q4	-25.59	-31.92	-19.26	<0.01	-16.19	-26.87	-5.51	0.003
	% of births attended by healthcare professionals	Q1	Reference				Reference			
	% of births attended by healthcare professionals	Q2	-2.11	-3.91	-0.30	0.022	-11.07	-15.10	-7.03	<0.01
	% of births attended by healthcare professionals	Q3	-2.09	-4.45	0.28	0.084	-12.16	-16.66	-7.66	<0.01
	% of births attended by healthcare professionals	Q4	-7.92	-11.36	-4.47	<0.01	-18.99	-24.48	-13.49	<0.01
Medical products, vaccines, and technologies	Measles vaccination coverage	Q1	Reference				Reference			
	Measles vaccination coverage	Q2	-6.49	-8.87	-4.12	<0.01	-8.64	-12.68	-4.59	<0.01
	Measles vaccination coverage	Q3	-6.27	-8.97	-3.58	<0.01	-5.41	-9.68	-1.14	0.013
	Measles vaccination coverage	Q4	-7.36	-10.19	-4.52	<0.01	-7.53	-11.94	-3.12	<0.01
Health human resources	Physicians per 1,000 inhabitants	Q1	-	-	-	-	Reference			
	Physicians per 1,000 inhabitants	Q2	-	-	-	-	-1.43	-6.16	3.31	0.554
	Physicians per 1,000 inhabitants	Q3	-	-	-	-	-0.02	-5.26	5.22	0.994
	Physicians per 1,000 inhabitants	Q4	-	-	-	-	2.53	-3.32	8.38	0.397
Health financing	Health expenditure per capita	Q1	Reference				Reference			
	Health expenditure per capita	Q2	0.53	-2.08	3.14	0.69	1.10	-3.23	5.44	0.618
	Health expenditure per capita	Q3	-2.78	-5.99	0.42	0.09	-2.49	-7.56	2.59	0.337
	Health expenditure per capita	Q4	-2.28	-6.31	1.75	0.27	-1.54	-7.50	4.42	0.612
	Public health expenditure—% of total expenditure	Q1	Reference				Reference			
	Public health expenditure—% of total expenditure	Q2	-1.95	-3.64	-0.25	0.02	-1.64	-4.99	1.72	0.339
	Public health expenditure—% of total expenditure	Q3	-2.85	-4.93	-0.78	0.01	-0.62	-4.40	3.16	0.746
	Public health expenditure—% of total expenditure	Q4	-2.60	-5.47	0.27	0.08	-2.58	-7.27	2.12	0.283
Social determinants	% of primary education in women	Q1	Reference				Reference			
	% of primary education in women	Q2	0.52	-1.17	2.21	0.55	-0.87	-3.31	1.57	0.484
	% of primary education in women	Q3	1.50	-0.96	3.95	0.23	1.93	-2.51	6.36	0.394
	% of primary education in women	Q4	6.87	3.30	10.45	<0.01	3.92	-1.98	9.82	0.193
	Ethnic fragmentation	Q1	Reference				Reference			
	Ethnic fragmentation	Q2	2.57	-5.25	10.39	0.52	-6.44	-14.67	1.78	0.125
	Ethnic fragmentation	Q3	-0.41	-8.27	7.46	0.92	-0.84	-10.61	8.93	0.866
	Ethnic fragmentation	Q4	9.93	-0.03	19.90	0.05	12.98	-0.24	26.20	0.054
	Linguistic fragmentation	Q1	Reference				Reference			
	Linguistic fragmentation	Q2	2.34	-5.17	9.85	0.54	-3.14	-11.51	5.22	0.461
	Linguistic fragmentation	Q3	-3.04	-10.69	4.61	0.44	-6.16	-15.93	3.61	0.217
	Linguistic fragmentation	Q4	8.80	-1.00	18.60	0.08	-3.34	-16.66	9.98	0.623
	Gini Coefficient	-	-	-	-	-	0.02	-0.33	0.36	0.923
Year		2000	63.95	54.96	72.94	<0.01	87.33	68.38	106.28	<0.01
		2001	-4.99	-6.78	-3.21	<0.01	-5.17	-7.61	-2.72	<0.01
		2002	-5.41	-7.15	-3.68	<0.01	-6.64	-9.56	-3.72	<0.01
		2003	-6.15	-7.78	-4.52	<0.01	-7.49	-10.33	-4.65	<0.01
		2004	-7.68	-9.40	-5.96	<0.01	-10.42	-14.82	-6.02	<0.01
		2005	-8.08	-9.74	-6.43	<0.01	-9.28	-12.48	-6.07	<0.01
		2006	-9.20	-10.71	-7.68	<0.01	-9.12	-11.65	-6.59	<0.01
		2007	-10.71	-12.29	-9.13	<0.01	-9.61	-12.31	-6.90	<0.01
		2008	-11.79	-13.60	-9.97	<0.01	-10.98	-13.81	-8.14	<0.01
		2009	-11.67	-13.51	-9.84	<0.01	-15.42	-19.42	-11.42	<0.01
		2010	-15.42	-17.12	-13.73	<0.01	-14.07	-16.48	-11.66	<0.01
Assessment of the model		AIC	2200.601				903.617			
		Bic	2354.227				1032.096			
		P R2	0.89				0.93			

Concerning infant mortality, <5 years, the determinants that evidence an association were: % of access to fresh water for quartiles 2 to 4 in comparison with quartile 1 (βa Q4 vs Q1 = -30.9; 95% CI: -48.02 to -13.79), % of measles vaccination coverage (**β**a Q2 vs Q1 = -8,02; 95% CI: -14.17 to -1.88), ethnical fragmentation (**β**a Q4 vs Q1 = 26,1; 95% CI: 8.9 to 43.21), and linguistic fragmentation (**β**a Q4 vs Q1 = 34,92; 95% CI: 16.14 to 53.7), democratization (**β**a not free vs partially free = 16,69; 95% CI: 6.08 to 27.31), and (**β**a not free vs free = 11.23; 95% CI: -0.82 to 23.29), health out-of-pocket expenditure (**β**a Q1 vs Q3 = 13.06; 95% CI: 1.79 to 24.3) and (**β**a Q1 vs Q4 = 17.71; 95% CI: 5.86 to 29.56), and the Gini coefficient (**β**a = 1.18; 95% CI: 0.22 to 2.13) (Table 3 and Table 4).

**Table 4:** Multivariate analysis for IMR < 5 years.

**Function of the health system**	**Variable**	**Categories**	**Model 1 (n = 139 countries)**				**Model 2 (n = 69 countries)**			
			**Beta**	**95% CI**		**p**	**Beta**	**95% CI**		**p**
**Health services coverage**	% of access to fresh water	Q1	Reference				Reference			
	% of access to fresh water	Q2	-21.48	-35.42	-7.53	0.003	-28.54	-39.45	-17.62	0.02
	% of access to fresh water	Q3	-27.50	-42.10	-12.91	<0.01	-31.04	-48.71	-13.38	<0.01
	% of access to fresh water	Q4	-30.91	-48.02	-13.80	0.053	-24.08	-56.52	8.35	0.14
	% of access to sanitation systems	Q1	-	-	-	-	Reference			
	% of access to sanitation systems	Q2	-	-	-	-	-14.63	-32.75	3.49	0.11
	% of access to sanitation systems	Q3	-	-	-	-	-22.17	-43.71	-0.62	0.04
	% of access to sanitation systems	Q4	-	-	-	-	-32.33	-58.01	-6.66	0.01
	Prenatal control coverage—one (1) visit	Q1	-	-	-	-	Reference			
	Prenatal control coverage—one (1) visit	Q2	-	-	-	-	0.61	-7.43	8.64	0.88
	Prenatal control coverage—one (1) visit	Q3	-	-	-	-	-5.40	-15.74	4.94	0.31
	Prenatal control coverage—one (1) visit	Q4	-	-	-	-	-2.07	-14.39	10.25	0.74
**Medical products, vaccines, and technologies**	Measles vaccination coverage	Q1	Reference				Reference			
	Measles vaccination coverage	Q2	-8.03	-14.17	-1.88	0.01	-17.60	-27.53	-7.68	<0.01
	Measles vaccination coverage	Q3	-6.31	-12.77	0.15	0.056	-16.40	-28.19	-4.62	<0.01
	Measles vaccination coverage	Q4	-6.33	-13.41	0.76	0.08	-23.03	-38.61	-7.45	<0.01
	DPT vaccination coverage	Q1	-	-	-	-	Reference			
	DPT vaccination coverage	Q2	-	-	-	-	-1.09	-10.06	7.89	0.81
	DPT vaccination coverage	Q3	-	-	-	-	-0.23	-11.24	10.78	0.97
	DPT vaccination coverage	Q4	-	-	-	-	10.41	-6.45	27.27	0.23
**Health human resources**	Physicians per 1,000 inhabitants	Q1	-	-	-	-	Reference			
	Physicians per 1,000 inhabitants	Q2	-	-	-	-	-6.72	-14.01	0.58	0.07
	Physicians per 1,000 inhabitants	Q3	-	-	-	-	-5.93	-16.38	4.52	0.27
	Physicians per 1,000 inhabitants	Q4	-	-	-	-	-0.90	-14.65	12.84	0.90
**Health financing**	Health expenditure per capita	Q1	Reference				Reference			
	Health expenditure per capita	Q2	-0.25	-6.45	5.95	0.937	-2.49	-11.73	6.74	0.60
	Health expenditure per capita	Q3	-12.48	-20.59	-4.36	<0.01	-6.60	-23.09	9.90	0.43
	Health expenditure per capita	Q4	-11.85	-23.19	-0.52	0.04	-2.46	-21.90	16.97	0.80
	Out-of-pocket health expenditure	Q1	-	-	-	-	Reference			
	Out-of-pocket health expenditure	Q2	-	-	-	-	5.01	-5.23	15.26	0.34
	Out-of-pocket health expenditure	Q3	-	-	-	-	13.07	1.79	24.34	0.02
	Out-of-pocket health expenditure	Q4	-	-	-	-	17.72	5.87	29.57	0.00
**Social determinants**	Ethnic fragmentation	Q1	Reference				Reference			
	Ethnic fragmentation	Q2	3.25	-7.79	14.28	0.564	-3.22	-23.31	16.86	0.75
	Ethnic fragmentation	Q3	-0.14	-10.22	9.94	0.978	-7.59	-29.02	13.84	0.49
	Ethnic fragmentation	Q4	26.11	9.00	43.22	<0.01	16.29	-8.68	41.25	0.20
	Linguistic fragmentation	Q1	Reference				Reference			
	Linguistic fragmentation	Q2	5.28	-4.42	14.97	0.286	0.14	-17.26	17.53	0.99
	Linguistic fragmentation	Q3	-2.25	-10.93	6.44	0.612	-3.35	-21.13	14.44	0.71
	Linguistic fragmentation	Q4	34.92	16.14	53.70	<0.01	7.75	-11.64	27.14	0.43
	Religious fragmentation	Q1	-	-	-	-	Reference			
	Religious fragmentation	Q2	-	-	-	-	2.20	-12.56	16.95	0.77
	Religious fragmentation	Q3	-	-	-	-	-2.79	-16.75	11.18	0.70
	Religious fragmentation	Q4	-	-	-	-	-4.72	-20.27	10.84	0.55
	Index of freedom	Not free	Reference				Reference			
	Index of freedom	Partially free	1.45	-3.53	6.43	0.57	16.70	6.09	27.31	<0.01
	Index of freedom	Free	1.34	-5.01	7.68	0.68	11.23	-0.83	23.30	0.07
	Gini Coefficient		-	-	-	-	1.18	0.23	2.14	0.02
	% of women employed	Q1	-	-	-	-	Reference			
	% of women employed	Q2	-	-	-	-	2.59	-11.49	16.66	0.72
	% of women employed	Q3	-	-	-	-	4.49	-9.71	18.69	0.54
	% of women employed	Q4	-	-	-	-	11.38	-4.14	26.90	0.15
**Year **		2000	80.90	63.02	98.77	0.1	47.44	-9.01	103.88	0.10
		2001	-2.64	-8.42	3.13	0.37	-7.52	-15.65	0.62	0.07
		2002	-6.41	-10.16	-2.67	<0.01	-6.92	-16.81	2.98	0.17
		2003	-8.57	-12.15	-4.99	<0.01	-7.28	-14.91	0.35	0.06
		2004	-11.87	-15.62	-8.12	<0.01	-9.56	-20.37	1.24	0.08
		2005	-15.77	-19.71	-11.83	<0.01	-13.22	-21.50	-4.94	<0.01
		2006	-17.69	-21.73	-13.65	<0.01	-16.77	-23.63	-9.90	<0.01
		2007	-17.64	-22.82	-12.46	<0.01	-14.52	-22.72	-6.31	<0.01
		2008	-20.85	-25.70	-16.00	<0.01	-21.29	-29.23	-13.35	<0.01
		2009	-20.81	-25.52	-16.10	<0.01	-23.49	-37.13	-9.84	<0.01
		2010	-28.49	-34.11	-22.87	<0.01	-22.84	-29.47	-16.20	<0.01
** Assessment of the model**		AIC	3190.498				1082.008			
		Bic	3356.096				1228.241			
		P R2	0.83				0.94			

As per the maternal mortality ratio, the determinants that evidenced a protective association include: % of access to fresh water for all the quartiles (**β**a Q2 vs Q1 = -147,3; 95% CI: -233.38 to -61.32), % of access to sanitation systems for all the quartiles, (**β**a Q3 vs Q1 = -171.15; 95% CI: -281.29 to -61), and birth attention by healthcare professionals for all the quartiles (**β**a Q4 vs Q1 = -231.23; 95%CI: -349.32 to 113.15). Having a more corrupt government (**β**a Q3 vs Q1 = 83.05; 95% CI: 33.10 to 133) and the highest fertility rates (**β**a Q4 vs Q1 219.94; 95% CI: 88.17 to 351.71) proved to be significant risk determinants as per MMR (Table 5).

**Table 5:** MMR multivariate model.

**Function of the health system**	**Variable**	**Categories**	**Model 1 (n = 114 countries)**				**Model 2 (n = 65 countries)**			
			**Beta**	**95% CI**		**p**	**Beta**	**95% CI**		**p**
**Coverage of health services**	% of access to fresh water	Q1	Reference				Reference			
	% of access to fresh water	Q2	-147.36	-233.38	-61.33	<0.01	-111.90	-242.00	18.20	0.092
	% of access to fresh water	Q3	-146.70	-251.42	-41.98	0.006	-59.90	-190.98	71.17	0.37
	% of access to fresh water	Q4	-133.00	-272.01	6.01	0.061	-22.07	-217.91	173.78	0.825
	% of access to sanitation systems	Q1	Reference				Reference			
	% of access to sanitation systems	Q2	-119.90	-214.81	-24.99	0.013	4.23	-125.48	133.95	0.949
	% of access to sanitation systems	Q3	-171.15	-281.30	-61.01	0.002	-94.04	-240.22	52.13	0.207
	% of access to sanitation systems	Q4	-170.64	-328.50	-12.79	0.034	-130.42	-338.09	77.26	0.218
	% of births attended by healthcare professionals	Q1	Reference				Reference			
	% of births attended by healthcare professionals	Q2	-133.33	-204.68	-61.99	<0.01	-72.93	-125.15	-20.72	<0.01
	% of births attended by healthcare professionals	Q3	-157.33	-251.36	-63.30	0.001	-62.33	-119.33	-5.32	0.032
	% of births attended by healthcare professionals	Q4	-231.24	-349.32	-113.16	<0.01	-76.88	-140.16	-13.59	0.017
**Human Resources**	Physicians per 1,000 inhabitants	Q1	-	-	-	-	Reference			
	Physicians per 1,000 inhabitants	Q2	-	-	-	-	-29.53	-135.88	76.81	0.586
	Physicians per 1,000 inhabitants	Q3	-	-	-	-	-30.80	-137.34	75.74	0.571
	Physicians per 1,000 inhabitants	Q4	-	-	-	-	-18.26	-124.96	88.44	0.737
**Health financing**	Health total expenditure	Q1	-	-	-	-	Reference			
	Health total expenditure	Q2	-	-	-	-	-66.97	-105.21	-28.73	0.001
	Health total expenditure	Q3	-	-	-	-	-5.72	-39.97	28.53	0.743
	Health total expenditure	Q4	-	-	-	-	0.49	-36.51	37.48	0.979
**Social determinants**	Index of corruption	Q1	-	-	-	-	Reference			
	Index of corruption	Q2	-	-	-	-	15.53	0.27	30.79	0.046
	Index of corruption	Q3	-	-	-	-	83.05	33.10	133.01	0.001
	Index of corruption	Q4	-	-	-	-	-68.64	-164.48	27.20	0.16
**Demographic transition**	Fertility rate	Q1	Reference				Reference			
	Fertility rate	Q2	10.70	-81.79	103.20	0.821	34.36	-53.96	122.68	0.446
	Fertility rate	Q3	8.44	-89.93	106.80	0.866	90.57	-0.92	182.06	0.052
	Fertility rate	Q4	219.94	88.17	351.71	<0.01	383.58	203.98	563.18	<0.01
**Year**		2000	551.80	405.55	698.05	<0.01	262.14	73.70	450.59	<0.01
		2005	-91.28	-137.76	-44.80	<0.01	-13.01	-28.65	2.63	0.103
		2010	-101.96	-146.09	-57.82	<0.01	-32.15	-45.45	-18.85	<0.01
**Assessment of the model**		AIC	2618.58				1045.2			
		Bic	2674.651				1109.314			
		P R2	0.85				0.92			

### Geospatial analysis

**Fig 5:**
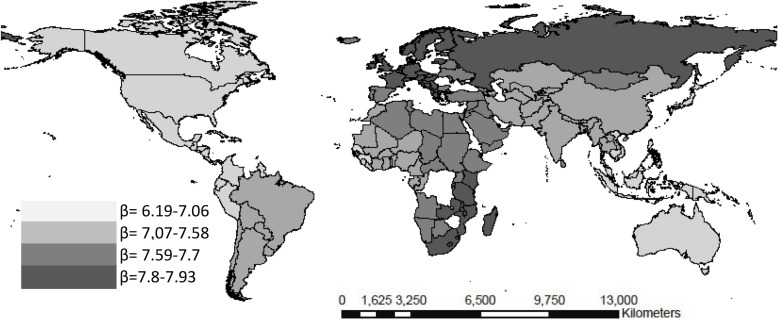
Weighted beta coefficients for the corruption index and maternal mortality rate per quartiles.

**Fig 6:**
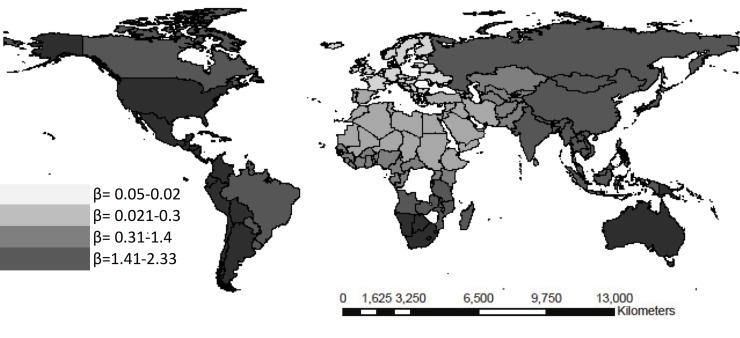
Weighted beta coefficients for cultural-ethnic fragmentation and infant mortality rate <5 years per quartiles.

**Fig 7:**
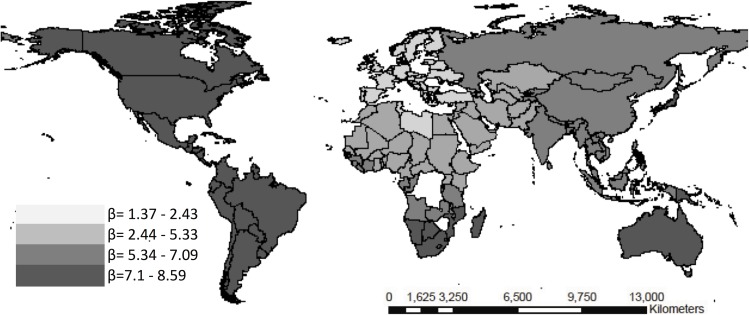
Weighted beta coefficients for the Gini coefficient and infant mortality rate <5 years per quartiles.

The African continent evidences a different trend vis-à-vis the rest of the continents as per the social determinants assessed and the health results. The corruption index shows a 6.01 MMR risk relationship in Africa with respect to Europe where it was **β**a -1.04 (95% CI -1.24 to 15,24) (see Fig 5). Regarding the < 5 years mortality rate and the cultural fragmentation index, the results evidenced a region effect for Latin America (**β**a 5.03 vs 0.34 95% CI—0.32 to 18.41) and Asia (**β**a 8.45 vs -1.32 95% CI—3.48 to 26.83) in comparison with Europe, Fig 6. Likewise, regarding the infant mortality rate, income inequality evidenced a region effect for Latin America (**β**a 12.34 vs 2.56 95% CI 0.21 to 22.3), North America (**β**a 7.61 vs 1,42 95% CI -1.42 to 34.7), and Asia (**β**a 11.87 vs 2.03 95% CI -2.8 to 23) (see Figs 5–7).

### Country level analyze

Quartile analysis for each variables included in this study in the countries where the information was available, S4 Table. Following, Quartile analysis of selected social determinants and an analysis of the relationship between health outcomes and health expenditures:

#### Social determinants

A difference between countries in each continent in relation to the behavior of social determinants was evident. For ethnic cultural fragmentation two (4.76%) European countries (Andorra and Monaco) were in Q4 vs 18 (42.85%) countries (Ireland, Sweden, Finland, Norway, Germany, Malta, Austria, Portugal, United Kingdom, Netherlands, Hungary, Iceland, Italy, Poland, France, Denmark, Cyprus, Greece) in Q1; 32 (62.7%) countries (Sudan, Gambia, The, Guinea-Bissau, Central African Republic, Nigeria, Cote d'Ivoire, Angola, South Africa, Mali, Benin, Congo, Rep., Somalia, Congo, Dem. Rep., Liberia, Ghana, Zambia, Mozambique, Cameroon, Uganda, Ethiopia, Gabon, Chad, Guinea, Malawi, Tanzania, Burkina Faso, Togo, Senegal, Djibouti, Madagascar, Kenya, Sierra Leone) in Africa were in Q4 vs. 4 (7.8%) countries (Comoros, Tunisia, Egypt, Arab Rep., Swaziland) in Q1; 3 (8.8%) countries (Bolivia, Suriname, Belize) in Latin America and The Caribbean were in Q4 vs 10 (27.02%) countries (Haiti, St. Kitts and Nevis, Paraguay, Saint Lucia, El Salvador, Barbados, Antigua and Barbuda, Chile and Honduras) in Q1; 8 (16%) countries (Indonesia, Pakistan, Libya, Nepal, Qatar, Afghanistan, Iran, Islamic Rep, Kyrgyz Republic) Asia were in Q4 vs 9 (20%) countries (Lebanon, Armenia, Saudi Arabi, Bangladesh, Hong Kong SAR, China, Korea, Rep, Korea, Dem and Japan) in Q1; one country (50%) of North America (United States) was in Q3 vs one (50%) country (Canada) in Q4 and 1 (7.1%) country (Micronesia) of Oceania was in Q4 vs 8 (57.1%) countries (Samoa, Tonga, Vanuatu, Solomon Islands, Kiribati, Marshall Island, Australia and Tuvalu) in Q1.

For linguistic fragmentation, 4 (10%) European countries (Bosnia and Herzegovina, Luxembourg, Andorra and Monaco) were in Q4 vs 14 (35%) countries (Ireland, Croatia, Norway, Malta, Portugal, United Kingdom, Hungary, Iceland, Italy, Poland, France, Denmark, Albania, Greece) in Q1; 32 (64%) countries in Africa (Sudan, The Gambia, Guinea-Bissau, Central African Republic, Nigeria, Cote d'Ivoire, Angola, South Africa, Mali, Benin, Congo, Republic., Congo, Dem. Rep, Liberia, Ghana, Zambia, Mozambique, Niger, Cameroon, Uganda, Ethiopia, Gabon, Chad, Namibia, Guinea, Tanzania, Burkina Faso, Togo, Senegal, Eritrea, Djibouti, Kenya, Sierra Leone) were in Q4 vs 5 (10%) countries (Comoros, Tunisia, Egypt, Arab Rep Somalia and Madagascar.) in Q1; no country in Latin America and The Caribbean was in Q4 vs 16 (53%) countries (Brazil, Puerto Rico, Jamaica, Trinidad and Tobago, Dominic Republic, St. Vincent and the Grenadines, Venezuela, RB, Uruguay, Guyana, Nicaragua, Costa Rica, Colombia, Barbados, Argentina, Antigua and Barbuda and Honduras); 8 (16.6%) countries in Asia (India, Indonesia, Pakistan, Nepal, Philippines, Lao PDR, Iran, Islamic, Kazakhtan) were in Q4 vs 10 (20.8%) countries (Yemen, Rep., Libya, Jordan, Armenia, Saudia Arabia, Bangladesh, Korea, Rep Korea, Dem West Bank and Gaza and Japan) in Q1; one country (50%) of North America (United States) was in the Q2 vs Canada (50%) that was in Q3 and 4 (21%) countries of Oceania (Micronesia, Guam, New Caledonia and Northern Mariana Islands) were in Q4 vs. 3 (15.78%) countries (Samoa, Kiribati and Marshall Islands) in Q1.

For religious cultural fragmentation, six (14.63%) countries (Georgia, Germany, Bosnia and Herzegovina, United Kingdom, Czech Republic and Netherlands) in Europe were in Q4 vs 11 (26.8%) countries (Ireland, Belgium, Norway, Malta, Portugal, Luxembourg, San Marino, Andorra, Iceland, Poland and Greece) who were in Q1; 19 (36.53%) countries (Central African Republic, Nigeria, Cote d'Ivoire, South Africa, Congo, Republic, Congo, Dem, Rep., Ghana, Zambia, Mozambique, Cameroon, Gabon, Chad, Zimbabwe, Namibia, Malawi, Mauritius, Lesotho, Togo, Kenya) Africa were in Q4 vs 16 (30.76%) countries (Comoros, Tunisia, The Gambia, Mauritania, Egypt, Arab Rep., Algeria, Morocco, Mali, Somalia, Cape Verde, Niger, Sao Tome and Principe, Seychelles, Equatorial Guinea, Senegal and Djibouti) that were in Q1; 8 (22%) of countries (Trinidad and Tobago, St. Kitts and Nevis, St. Vincent and the Grenadines, Suriname, Guyana, The Bahamas, Barbados and Antigua and Barbuda) in Latin America and The Caribbean were in Q4 vs 8 (22.2%) of countries (Peru, Mexico, Bolivia, Paraguay, Ecuador, Venezuela, Colombia and Argentina) were in Q1; 6 (12.5%) countries (Kuwait, Lebanon, Singapore, Malaysia, China and Korea, Dem. Rep) of Asia were in Q4 vs 16 (33.3%) countries (Yemen, Rep., Cambodia, Libya, Nepal, Uzbekistan, Qatar, Jordan, Saudi Arabia, Bangladesh, Thailand, Turkey, Burma, Mongolia, West Bank and Gaza, Turkmenistan and Iran, Islamic Rep) who were in Q1; 2 (100%) countries of North America (USA and Canada) were in Q4; and 8 (42.1%) countries (Micronesia, Samoa, Vanuatu, Solomon islands, Palau, Américan Samoa, New Zealand and Australia) of Oceania were in Q4 and no country was in the Q1.

For corruption index behavior we did not found change in quartiles by period between 2007 and 2010, therefore we only show the result of 2010 corruption index. This indicator is higher if the corruption perceived is low. 21 (52.5%) countries (Ireland, Belgium, Sweden, Switzerland, Finland, Norway, Germany, Malta, Slovenia, Austria, Portugal, Luxembourg, United Kingdom, Netherlands, Estonia, Iceland, Poland, France, Denmark, Cyprus and Spain) of Europe were in Q4 vs 1 (2.5%) country (Ukraine) in Q1; 2 (5.5%) countries (Botswana and Mauritius) of Africa were in Q4 vs 21 (38.8%) countries (Sudan, Comoros, Guinea-Bissau, Mauritania, Central African Republic, Burundi, Nigeria, Cote d'Ivoire, Angola, Congo, Rep, Somalia, Congo, Dem Rep, Cameroon, Chad, Zimbabwe, Guinea, Equatorial Guinea, Togo, Kenya and Sierra Leone) were in Q1; 6 (23.07%) countries (Puerto Rico, Dominica, Uruguay, Costa Rica, Barbados and Chile) in Latin America and The Caribbean are found in Q4 vs. 4 (15.38%) countries (Haiti, Paraguay, Venezuela and Honduras) that were in Q1. 10 countries in Asia were found (21.27%) in Q4 (Israel, Qatar, Oman, Brunei Darussalam, Singapore, United Arab Emirates, Hong Kong, Korea, Dem. Rep., Japan, Bhutan) vs 19 (40.42%) countries (Yemen, Rep., Cambodia, Pakistan, Iraq, Libya, Nepal, Uzbekistan, Philippines, Tajikistan, Bangladesh, Azerbaijan, Russian Federation, Turkmenistan, Maldives, Afghanistan, Lao PDR, Iran, Islamic Rep., Kyrgyz Republic, Myanmar) in Q1. The two countries (100%) of North America (US and Canada) were in Q4. 2 (25%) countries of Oceania (New Zealand and Australia) were in Q4 vs 1 (12.5%) country (Papua New Guinea) was in the Q1.

To evaluate the democratization we used freedom index from 2000 to 2010, but no significant changes in levels of freedom occurred in the countries (S4). We only show the result of 2010 freedom index; Europe: 37 (82.22%) countries were listed as free countries (Finland, France, Andorra, Austria, Belgium, Bulgaria, Croatia, Cyprus, Czech Republic, Denmark, Estonia, Germany, Greece, Hungary, Iceland, Ireland, Italy, Latvia, Liechtenstein, Lithuania, Luxembourg, Malta, Monaco, Montenegro, Netherlands, Norway, Poland, Portugal, Romania, San Marino, Serbia, Slovak Republic, Slovenia, Spain, Sweden, Switzerland and United Kingdom), 7 (15.5%) countries were listed as partially free (Albania, Bosnia and Herzegovina, Georgia, Kosovo, Macedonia, Moldova and Ukraine) and 1 (2.2%) was ranked not free country (Belarus); Africa: 9 (17.3%) countries (Benin, Botswana, Cape Verde, Ghana, Mali, Mauritius, Namibia, Sao Tome and Principe and South Africa) were listed as free, 23 (44.23%) countries were listed as part free (Burkina Faso, Burundi, Central African Republic, Comoros, The Gambia, Guinea, Guinea-Bissau, Kenya, Lesotho, Liberia, Madagascar, Malawi, Morocco, Mozambique, Niger, Nigeria, Senegal, Seychelles, Sierra Leone, Tanzania, Togo, Uganda and Zambia) and 20 (38.46%) listed as unfree countries (Algeria, Angola, Cameroon, Chad, Congo, Dem. Rep., Congo, Rep., Cote d'Ivoire, Djibouti, Egypt, Arab Rep., Equatorial Guinea, Eritrea, Ethiopia, Gabon, Mauritania, Rwanda, Somalia, Sudan, Swaziland, Tunisia and Zimbabwe). Latin America and the Caribbean: 22 (66.6%) countries were listed as a free (Antigua and Barbuda, Argentina, The Bahamas, Barbados, Belize, Brazil, Chile, Costa Rica, Dominica, Dominican Republic, El Salvador, Grenada, Guyana, Jamaica, Panama, Peru, St. Kitts and Nevis, St. Lucia, St. Vincent and the Grenadines, Suriname, Trinidad and Tobago and Uruguay), 10 (30.3%) countries listed as partly free (Bolivia, Colombia, Ecuador, Guatemala, Haiti, Honduras, Mexico, Nicaragua, Paraguay and Venezuela) and one (3%) listed as unfree country (Cuba). Asia: 7 (14.58%) countries were listed as free countries (India, Indonesia, Israel, Japan, Korea, Dem Rep, Mongolia and Taiwan), 16 (33.3%) countries were listed partially free (Armenia, Bangladesh, Bhutan, East Timor, Kuwait, Kyrgyz Republic, Lebanon, Malaysia, Maldives, Nepal, Pakistan, Philippines, Singapore, Sri Lanka, Thailand and Turkey) and 25 (52.08%) classified unfree countries (Afghanistan, Azerbaijan, Bahrain, Brunei Darussalam, Burma, Cambodia, China, Iran, Islamic Rep., Iraq, Jordan, Kazakhstan, Korea, Rep., Lao PDR, Libya, Oman, Qatar, Russian Federation, Saudi Arabia, Syrian Arab Republic, Tajikistan, Turkmenistan, United Arab Emirates, Uzbekistan, Vietnam and Yemen, Rep). North America: the two (100%) countries were listed free (Canada, USA). Oceania: 10 (71.42%) countries were listed as free (Australia, Kiribati, Marshall Islands, Micronesia, Fed, Sts, Nauru, New Zealand, Palau, Samoa, Tuvalu and Vanuatu.), 4 (28.57%) countries were listed as partly free (Fiji, Papua New Guinea, Solomon Islands and Tonga) and no country was ranked not free.

In the analysis of the Gini coefficient as a result of income inequality; no country in Europe was in the Q4 vs 23 (62.1%) countries (Belgium, Sweden, Croatia, Switzerland, Montenegro, Finland, Norway, Ukraine, Germany, Slovenia, Austria, Luxembourg, Czech Republic, Netherlands, Belarus, Bulgaria, Serbia, Hungary, Romania, France, Slovak Republic, Moldova, Denmark) who were in Q1; 18 (37.5%) countries of Africa (Comoros, The Gambia, Rwanda, Central African Republic, Nigeria, Botswana, South Africa, Congo, Rep., Cape Verde, Zambia, Mozambique, Sao Tome and Principe, Zimbabwe, Seychelles, Namibia, Swaziland, Lesotho and Kenya) were in Q4 vs. 4 countries (Egypt, Arab Rep, Burundi, Mali, Ethiopia) who were in Q1; 16 (69.56%) countries of Latin America and The Caribbean (Brazil, Haiti, Dominican Republic, Peru, Mexico, Bolivia, Paraguay, Ecuador, Suriname, El Salvador, Costa Rica, Colombia, Panama, Belize, Chile and Honduras) were in Q4 vs no countries in Q1; One (2.6%) country of Asia (Malasya) was found in Q4 vs 10 (26.31%) countries (Pakistan, Iraq, Nepal, Armenia, Tajikistan, Bangladesh, Korea, Rep., Japan, Afghanistan and Kazakhstan) who were in Q1; any countries in North America was in the Q4 vs one (50%) country (Canada) was in Q1 (S4 Table).

#### Health result and Health expenditure

Countries with better indicators of health outcomes and behaviors of health expenditure per capita were for Latin America: Chile Costa Rica Uruguay, Cuba and Costa Rica, countries with worse health indicators and higher health spending per capita were: Paraguay, Bolivia, Nicaragua, Haiti, Belize and Suriname. For North America excluding Mexico (considered within ALC), both countries had adequate indicators in health outcomes but United States showed an increase in spending on health and pocket health spending between 2000 and 2010. For Europe, countries with better health outcomes and improved performance of health expenditure per capita were: Switzerland, Norway, Sweden, Denmark and the UK and countries with poorer health outcomes and poor health expenditure per capita are Georgia, Moldova, Ukraine and Albania

For African countries with better health outcomes and improved performance of health expenditure per capita: Arabian Republic of Egypt, Algeria, Morocco, Sao Tome and Principe, Dijibouti; and countries with worst health indicators and misbehavior of health expenditure per capita were: Chad, Sierra Leone, Central African Republic, Burundi, Dem Rep Congo, Nigeria, Cote d'Ivoire and Guinea. For Asian countries with better health outcomes and improved performance of health expenditure per capita were: Sri Lanka, Tajikistan, Uzbekistan, Kyrgyz Republic, China, Vietnam and Japan and countries with worse indicators of health outcomes and increased spending on health per capita were: Afghanistan, Iraq, Timor-Leste, Yemen, Rep, Lao PDR, Indonesia, Bangladesh, Pakistan, Bhutan and Philippines;. countries like South Korea, India and Syria have adequate indicators of health outcomes with high health expenditure per capita for all periods observed 2000–2010. For Oceania countries with better health outcomes and improved performance of health expenditure per capita were: Micronesia, Fed Sts, New Zealand and Australia and countries with worst health indicators and misbehavior of health expenditure per capita were: Papua New Guinea, Tonga, Samoa and Vanuatu (S4 Table).

This ecological analysis explains the relationship among the functioning of the health systems and four social determinants that are not classically related with the performance of the system itself in terms of health results through 154 countries around the world. Basically, the coverage of health services, measured through the sustainable access to water is associated to a lower mortality during all the periods assessed. Leadership and governance, measured through the corruption index (that is, governance in less corrupt scenarios) is associated to a lower maternal mortality [39]. Countries with lower ethno-linguistic cultural fragmentation were likewise associated to a lower infant mortality rate, <1 year and lower than <5 years. Health human resources measured through the density of physicians, and health financing, measured through less out-of-pocket payments, and health expenditure per capita are associated to a decrease of mortality in the three scenarios; these findings are consistent with other studies [40, 41]. Sustainable access to fresh water and sanitation services were significantly associated to the <1 year IMR, <5 years IMR, and the MMR, when the model was adjusted by other variables, presumably, due to several reasons [42, 43] The high incidence and prevalence rates of acute diarrheal disease are commonly observed in places with limited access to fresh water and sanitation services [43].

The help from donations or governments is associated to higher access to fresh water but not necessarily to sanitation [43]. The acute diarrheal disease derived from contaminated water represents, in itself, 19% of the deaths of children < 5 years of age, and 1% of neonatal deaths [44]. Other studies at the ecological level have also shown that the MMR is strongly associated to the sustainable access to fresh water and sanitation systems since access to fresh water is a fundamental pillar of maternal health [45]. Birth practices at unhygienic facilities that are not duly equipped to provide a sterile milieu for a postpartum mother commonly contribute to the high maternal mortality rates. The mothers who cannot breastfeed their children run the risk of using water that is not suitable for special formula feeding in low-income countries as a way to prevent the vertical transmission of HIV [46].

The financing of the health system is a consistent finding in the three models. The health expenditure per capita and the out-of-pocket health expenditure were significantly related to the mortality results, but once both were included in the multivariate models, the health expenditure per capita better explained the behavior of the < 1 year IMR and the < 5 years IMR. This finding is not indicative of the fact that the out-of-pocket health expenditure or the overall expenditure are not important in order to assess the impact, in economic terms, of a health system, but that the variable that describes the strongest association in the models selected is the health expenditure per capita. It is observed that the sustainable health expenditure per capita reduces the infant mortality rate. This may be caused by the fact that the economic situation of the country as per financial sustainability has incidence on the management for the rendering of accessible and quality health services [47].

The out-of-pocket health expenditure has become important as per the comprehensive assessment of a health system, incurring in a catastrophic and impoverishing expense for the household, and the scope of health universal coverage. In African countries, where the economic systems are weak and, therefore, incapable of supporting a health system, systems require direct payments from the citizens for health care. A study that included 15 African countries calculated the prevalence of health out-of-pocket expenditure and its effects on the household economy; it was found that between 23% and 68% of the households resorted to loans and to selling household equipment to have access to health services [46, 47]. This situation generates negative effects in two ways: 1) The loss of direct economic income due to labor losses or labor absence of the sick individuals, and 2) the worsening of the impoverishment of the family unit because of acquiring external debts with no labor-related production generating social inequalities [48].

On the other hand, it has been evidenced that the density of physicians significantly reduces infant and maternal mortality. Nevertheless, none of the health-related results in this study, after controlling other indicators of the health system, had a significant relationship. This may be due to the quality of the information for these indicators since they had over 10% of missing data concerning the number of observations, or because the observation period was relatively short to be able to note any effect on the response variables [49]. Nevertheless, concerning MMR, it can be evidenced how relevant health human resources are since birth attention by healthcare professionals has a strong relationship with the reduction of maternal deaths in this study. This has also been evidenced in other studies [50–53]. It is interesting that Farahani et al. (2009) [4] have examined the short and long-term effects of health human resources, evidencing that these may have more long-term benefits within the management structures of the health system and, therefore, as per the health results.

The high fertility rates have been noted as related to high infant and pregnant women death rates. This association is not easy to explain since this relationship arises in countries where the infant mortality rate is high due to social and economic factors: these families have multiple children as per replacing the losses. Thus, biological risk factors arise such as multiparity and complications while attending births, and social risk factors such as malnutrition and poverty thus becoming a vicious cycle [54].

Democratization was relevant vis-à-vis the reduction of the infant mortality rate < 5 years, a relevant finding since there is no consistent evidence in this regard. Nevertheless, the relationship of the process of democratic governments has been explored in relation to the behavior of life expectancy. Democratization becomes relevant as per the reduction of infant mortality in terms of a country being freer. This may be due to the strengthening of public health in some countries with a higher level of democracy through the issuance of health policies, nutrition, and the prevention of infectious-contagious diseases [55]. Political decisions concerning the collective provision of education, social security, housing, etc. may influence the health of the population by providing protection against health risks, and the increase of resiliency [56]. This process is interrupted in countries with armed conflicts, with oppressing political regimes due to particular needs, or due to the polarization of the executive or judicial power [57].

Corruption is widely defined by “Transparency International” as the abuse of entrusted power for private gain [58]. Our results are limited to corruption in the public sector although it must be acknowledged that corruption in the private sector is reported in other studies and it must be taken into consideration for health systems within the assurance and rendering of the services. We have found in our study that a government is perceived as more corrupt (that is, with a lower CPI score) has a stronger association with the increase of the maternal mortality ratio.

Since the health systems are managed by the public sector and they require a strong commitment and strong resources, a corrupt government runs the risk of diverting public health resources for private gains [59]. Our findings suggest that a transparent government is an essential component of the strengthening of the health system and an important way to improve the health of the population. Three fourths of the countries of the world have a CPI score under five, meaning a serious corruption level [60]; as a result, it has been acknowledged by the UN that the fight against corruption must be the central focus for international aid and development [51]. Corruption is systematic and exists within and through the scales and sectors of the government; therefore, it requires the effort of the different actors and sectors. Private vertical programs are frequently fast and effective since they often operate off the public sphere. However, an undesired consequence of this approach could be the ease to generate a corruption agent in the public sphere. An effective strategy, reported in several countries, is the strengthening of public health as a discipline capable of generating health policies and systems for the oversight of the administrative processes of the health systems by demanding transparency and accountability [61]. Contributions made to the empirical evidence of the social determinants have explained the consistent behavior of the lower life expectancy and the higher infant mortality rates in groups that have their basic needs met, and that even have income levels that are higher than the national average in their countries, but that live in highly unequal societies as, for example, the population of African descent of the United States and England [58, 59].

On the other hand, the relationship between the main political conditions and the infant mortality rate < 5 years also evidences statistically significant associations. Our results show that the political world history tending towards democratic processes has had an effect on the reduction of the infant mortality rate < 5 years; the countries that have partially-free or not free regimes have higher infant mortality rates [61]. Some authors have connected the effect of democratization on the results of life expectancy at birth, finding consistency as per the improvement of health conditions in countries that have efforts concerning the establishment of democratic governments, egalitarian social inclusion, the cessation of armed conflicts, the creation of international agreements for economic growth, such as the European Union, and the transition of communist countries to becoming democratic countries, as the Soviet Union and East Germany with the increase of life expectancy at birth and quality of life indicators at the national level [62, 63].

Little has been explored about cultural fragmentation and its effect on performance and, therefore, on health indicators. This study makes clear the relationship among cultural fragmentation, IMR, and MMR. This relationship has many explanations both from the capitalist economic model, which partly explains the structure of the health systems and the healthcare models that differ according to the ability to pay of the user [64, 65], as the loss of social cohesion due to the social exclusion of ethnic minorities [66, 67]. The cultural fragmentation phenomenon has been studied in countries such as India, as well as the post-modern processes of the last three decades such as migration and globalization. The latter has been identified as a factor related to the increase of said cultural fragmentation and, in turn, the loss of acknowledgment of cultural beliefs. This has allowed that ethnic minorities evidence a higher obstacle as per having access to national public entities [68]. There are particular processes in Latin America such as the existence of ethnic minorities which are a challenge for the health systems due to the access barriers generated by geographical, language, and economic factors[69]. Some strategies implemented to solve these problems have been decentralizing both the financing and the rendering of health services; nonetheless, said measure has had contrary effects and some countries have decided to reconsider it [70].

Sustainable development economists have joined forces to research on ethnic diversity, or "fragmentation" as a possible cause of corruption, political instability and poor economic performance. Political scientists have debated for years about the possible links between ethnic diversity (or structure) and civil violence, democratic stability, and political systems [71]. The effect of cultural fragmentation is unclear in health status, however if has been highly correlated with poorly functioning processes of governance systems and poor socioeconomic conditions. Somalia and Botswana are two countries in which we can observe this complex relationship; in the 1960 Soviet ethnographers declared Somalia homogeneous in language, religion and cultural customs, but its economic growth has been poor and post-Civil War 1990 it was found that ethnic fragmentation by different clans has generated a chaotic social situation that associated with poor sustainable economic growth with poor indicators in education and health, particularly in child mortality <5 years, increasing access barriers in two ways 1) ideological differences between clans 2) hard empowerment health systems and education within the clans by the State [72]. Furthermore Botswana has presented better economic growth, but slowed down in the last 30 years; has a large ethnic Tswana and has been codified as homogeneous culturally, however—the Tswana—are divided into eight sub tribes who are socially and politically different, struggles between tribes for political and social representation in situations and processes of the State have generated political and social tensions throughout history, but likewise interesting strategies of political, social and ethnic inclusion which has resulted in pluralistic governance systems with better economic, health and social performance [72].

Providing physical, human and technological resources for the provision of health care resources can be deteriorate in societies with high levels of cultural fragmentation, whether by barriers of cultural access or purely geographical as documented in Latin America and Africa countries [73]. The lack of governance by the State on ethnic minorities also has a negative effect on the governance of the health system in these populations and territories in which they reside, creating an environment conducive to corruption, producing a lack of social protection of the state [74]. However, it is likely that this relationship is much more complex than here discussed and operate differently in different social contexts. For example, it is known that certain countries have managed to deal effectively with ethnic pluralism and even establish it as a cultural asset in a delicate balance with the assimilation process [75, 76], so that such companies may even be a input for collective efficacy. Bolivia may have about their new attitude to ethnic pluralism government, and against the white minority. groups. It remains to understand what the impact of this type of Governments on the operation of state and citizenship law [77]. Finally, it is not known what the best strategies that states can then re-organized historical ethnic conflicts, to be efficient and promote social justice, as happened in South Africa or currently Nigeria [78].

For all the above and having exposed its complexity, this relationship can be explored limitedly in this study and other studies need to understand their role. So this is only a hypothesis that requires further not only epidemiological but political science studies and sociology.

### Limitations of the study

This study depended on the availability of the data in the countries; therefore, it was not possible to include all the variables that could explain the health results approached. In addition, there were countries that were excluded due to the absence of data of some explanatory variables (n = 154 countries). This may lead to a selection bias since it is very probable that the countries that do not report data for the indicators are the countries with the worst health condition.

## Conclusions

The functions of the health system are related to own social processes of the countries and regions of the world [65]. The health systems and the States must make great efforts in order to guarantee the access to fresh water and sanitation services [65]. The ethno-linguistic fragmentation, corruption, and the detriment of democracy, are determinants as the per the functioning of the health system and, therefore, the increase of infant and maternal deaths. The historical processes of the societies as per the establishment of legitimate, transparent, and inclusive government systems may be relevant characteristics of the countries and regions that would give way to the establishment of effective and sustainable public policies to achieve the required health condition. Africa evidences a different behavior in relation to other regions and continents of the world. This is relevant concerning the measures to be adopted in order to achieve improvement in terms of health results because the social, financial, and cultural problems must be approached first before trying to establish organized operating systems to guarantee health-related results based on a system.

The Sweden, Norway, Denmark and Holland countries are the countries with better health indicators, democratization, perception of corruption and cultural fragmentation, Bolivia, Sierra Leone, Democratic Republic of Congo, Chad, Nigeria and Cote d'Ivoire countries were countries with worst health indicators, democratization and perception of corruption. The countries of Europe and North America have a better performance in terms of health outcomes, democratization and corruption. The countries of Latin America and Asia countries were heterogeneous results with excellent health indicators and social determinants such as Uruguay, Chile, Costa Rica for Latin America and China, Japan and South Korea to Asia vs Bolivia, Suriname and Belize for Latin America and North Korea, Afghanistan, Indonesia, Iraq and Yemen with poor health indicators and social determinants.

The information systems of health systems must be strengthened since only three indicators of all the indicators assessed and selected do evaluate the functions of the systems (vaccination coverage, coverage of births attended by healthcare professionals, and prenatal control coverage). This recommendation is made since the result indicators as per health are not necessarily assessing the functions of the health systems and mistaken inferences or conclusions may be reached.

## Supporting Information

S1 Table This is the S1 Table. List of countries selected for this study.
(DOCX)

S2 Table This is the S2 Table. Description of the IMR <1 year, IMR <5 years, and MMR tendencies between years 1990 to 2010
(DOCX)

S3 Table This is the S3 Table. Bivariate analysis per health result
(DOCX)

S4 Table This is the S4 Table. Quartile analyse per variable of health system performance and social determinants.
(DOCX)
