# Supplementary material for: Determinants of Performance of Health Systems Concerning Maternal and Child Health: A Global Approach
Source: PLoS One. 2015 Mar 30;10(3):e0120747. doi: 10.1371/journal.pone.0120747 (PMC4378969; doi:10.1371/journal.pone.0120747)
Supplement: S1 Table — List of countries selected for this study. (DOCX) [file pone.0120747.s001.docx]

Supplement 1. List of countries selected for this study.

| **Region** | **Country** | **Region** | **Country** | **Region** | **Country** |
| --- | --- | --- | --- | --- | --- |
| Africa | Congo | North America | United States | Asia | Myanmar (Burma) |
|  | Cameroon |  | Canada |  | Cambodia |
|  | Cape Verde | Latin America | Costa Rica |  | Iran |
|  | Djibouti |  | El Salvador |  | Iraq |
|  | Egypt |  | Peru |  | Jordan |
|  | Gabon |  | Brazil |  | Laos |
|  | Guinea |  | Dominican Republic |  | Nepal |
|  | Libya |  | Guyana |  | Qatar |
|  | Mongolia |  | Mexico |  | Singapore |
|  | Mali |  | Nicaragua |  | Thailand |
|  | Mauritania |  | Chile |  | Turkey |
|  | Mozambique |  | Colombia |  | Uzbekistan |
|  | Nigeria |  | Cuba |  | China |
|  | Rwanda |  | Ecuador |  | Georgia |
|  | Senegal |  | Guatemala |  | Israel |
|  | Sudan |  | Haiti |  | Japan |
|  | Togo |  | Jamaica |  | Kuwait |
|  | Uganda |  | Paraguay |  | Pakistan |
|  | Zambia |  | Panama |  | Philippines |
|  | Benin |  | Trinidad and Tobago |  | Saudi Arabia |
|  | Burundi |  | Uruguay |  | United Arab Emirates |
|  | Chad |  | Honduras |  | Tajikistan |
|  | Comoros | Europe | Bulgaria |  | Turkmenistan |
|  | Central African Republic |  | Finland |  | Vietnam |
|  | Ethiopia |  | Germany |  | Bhutan |
|  | Kenya |  | Iceland |  | Sri Lanka |
|  | Lesotho |  | Slovakia |  | India |
|  | Madagascar |  | Montenegro |  | Kyrgyzstan |
|  | Malawi |  | Portugal |  | Kazakhstan |
|  | Morocco |  | Switzerland |  | Maldives |
|  | Niger |  | Cyprus |  | Russia |
|  | São Tome and Principe |  | Ireland |  | Syria |
|  | Burkina Faso |  | France |  | Yemen |
|  | Swaziland |  | Greece |  |  |
|  | Gambia, The |  | Croatia |  |  |
|  | Ghana |  | Lithuania |  |  |
|  | Ivory Coast |  | Malta |  |  |
|  | Liberia |  | Netherlands |  |  |
|  | Mauritius |  | Slovenia |  |  |
|  | Guinea-Bissau |  | Spain |  |  |
|  | South Africa |  | Sweden |  |  |
|  | Sierra Leone |  | United Kingdom |  |  |
|  | Tunisia |  | Belarus |  |  |
|  | Tanzania, United Republic of |  | Denmark |  |  |
|  | Namibia |  | Estonia |  |  |
| Oceania | Solomon Islands |  | Czech Republic |  |  |
|  | Kiribati |  | Hungary |  |  |
|  | Western Samoa |  | Luxembourg |  |  |
|  | Indonesia |  | Moldova |  |  |
|  | Papua New Guinea |  | Macedonia |  |  |
|  | Malaysia |  | Norway |  |  |
|  | Vanuatu |  | Serbia |  |  |
|  | Tonga |  | Ukraine |  |  |
