# Supplementary material for: Determinants of Performance of Health Systems Concerning Maternal and Child Health: A Global Approach
Source: PLoS One. 2015 Mar 30;10(3):e0120747. doi: 10.1371/journal.pone.0120747 (PMC4378969; doi:10.1371/journal.pone.0120747)
Supplement: S2 Table — Description of the IMR <1 year, IMR <5 years, and MMR tendencies between years 1990 to 2010 (DOCX) [file pone.0120747.s002.docx]

Supplement 2. Description of the IMR <1 year, IMR <5 years, and MMR tendencies between years 1990 to 2010.

| **Variable/indicator** | **WORLD** | | | | | **LATIN AMERICA** | | | | |
| --- | --- | --- | --- | --- | --- | --- | --- | --- | --- | --- |
|  | **Average (Interquartile range) 2005** | **DS** | **Median** | **Range** | | **Average (Interquartile range) 2005** | **DS** | **Median** | **Range** | |
| **Results** | | | | | | | | | | |
| **Infant mortality rate <1 year** | | | | | | | | | | |
| 1990 | 48.84 | 39.49 | 37 | 5 | 165 | 34.87 | 20.29 | 29 | 11 | 100 |
| 1995 | 44.4 | 37.39 | 31 | 4 | 153 | 29.15 | 16.91 | 25 | 8 | 87 |
| 2000 | 39.16 | 34.23 | 25.5 | 3 | 143 | 24.3 | 13.78 | 21 | 7 | 75 |
| 2005 | 33.21 | 29.99 | 20 | 2 | 134 | 20.75 | 11.43 | 19 | 5 | 66 |
| 2010 | 28.22 | 26.27 | 17 | 2 | 123 | 18.48 | 11.79 | 16 | 5 | 73 |
| **Infant mortality rate < 5 years** | | | | | | | | | | |
| 1990 | 71.33 | 67.42 | 46 | 6 | 326 | 44.66 | 29.72 | 33 | 13 | 144 |
| 1995 | 64.93 | 63.95 | 38.5 | 5 | 279 | 36.51 | 24.29 | 30 | 11 | 124 |
| 2000 | 56.77 | 57.26 | 31 | 4 | 234 | 29.84 | 19.31 | 25 | 8 | 105 |
| 2005 | 47.32 | 48.85 | 24 | 3 | 216 | 25.12 | 15.71 | 22 | 7 | 91 |
| 2010 | 39.37 | 41.98 | 20 | 3 | 193 | 24.3 | 28.29 | 19 | 6 | 175 |
| **Maternal mortality ration** | | | | | | | | | | |
| 1990 | 333.31 | 445.53 | 96.5 | 6 | 2300 | 143.8 | 140.54 | 98 | 29 | 670 |
| 1995 | 300.24 | 415.09 | 89 | 2 | 2400 | 121.7 | 119.54 | 89 | 28 | 580 |
| 2000 | 259.08 | 356.08 | 80.5 | 4 | 2200 | 111.32 | 98.45 | 85 | 24 | 510 |
| 2005 | 213.95 | 288.04 | 69.5 | 2 | 1600 | 100.09 | 89.08 | 79 | 21 | 470 |
| 2010 | 178.99 | 237.99 | 69.5 | 2 | 1200 | 96.74 | 79.54 | 82 | 20 | 420 |
| **Life expectancy at birth** | | | | | | | | | | |
| 1990 | 64.85 | 9.99 | 68.29 | 32.61 | 78.83 | 68.98 | 4.69 | 70.35 | 54.42 | 75.72 |
| 1995 | 65.7 | 10.13 | 68.62 | 31.23 | 79.53 | 70.22 | 4.35 | 70.42 | 56.09 | 76.76 |
| 2000 | 66.99 | 10.26 | 70.44 | 38.11 | 81.07 | 71.57 | 4.38 | 70.98 | 57.42 | 77.88 |
| 2005 | 68.42 | 10.11 | 71.59 | 42.14 | 81.97 | 72.8 | 4.27 | 72.41 | 59.28 | 78.68 |
| 2010 | 70.06 | 9.47 | 73.07 | 44.83 | 83.15 | 73.89 | 3.98 | 73.9 | 61.86 | 79.28 |
| **Explanatory** | | | | | | | | | | |
| **Health human resources** | | | | | | | | | | |
| **Density of nurses / midwives (per 1,000 inhabitants)** | | | | | | | | | | |
| 1990 | 48.17 | 66.9 | 48.17 | 0.86 | 95.48 | - | - | - | - | - |
| 1995 | 14.5 | . | 14.5 | 14.5 | 14.5 | - | - | - | - | - |
| 2000 | 2.92 | 2.82 | 1.83 | 0.53 | 11.03 | 2.24 | 1.26 | 1.74 | 0.9 | 4.71 |
| 2005 | 3.51 | 3.13 | 2.74 | 0.043 | 10.91 | 2.96 | 2.66 | 2.96 | 1.08 | 4.85 |
| 2010 | 4.07 | 4.06 | 3.24 | 0.043 | 17.48 | 3.02 | 2.86 | 1.97 | 0.14 | 9.05 |
| **Density of physicians (per 1,000 inhabitants)** | | | | | | | | | | |
| 1990 | 1.7 | 4.08 | 0.97 | 0.007 | 47.35 | 1.12 | 0.82 | 0.9 | 0.08 | 3.68 |
| 1995 | 1.67 | 1.36 | 1.33 | 0.034 | 5.81 | 1.22 | 0.93 | 1.03 | 0.16 | 5.18 |
| 2000 | 2.04 | 1.36 | 2.1 | 0.021 | 5.897 | 1.45 | 1.26 | 1.19 | 0.45 | 5.89 |
| 2005 | 1.73 | 1.37 | 1.56 | 0.024 | 5 | 1.69 | 1.17 | 1.6 | 0.37 | 3.21 |
| 2010 | 1.81 | 1.59 | 1.59 | 0.008 | 7.739 | 2 | 1.63 | 1.6 | 0.21 | 6.72 |
| **Infrastructure** | | | | | | | | | | |
| **Number of beds (per 1,000 inhabitants)** | | | | | | | | | | |
| 1990 | 4.37 | 3.9 | 2.72 | 0.24 | 16.7 | 3.37 | 2.23 | 2.85 | 0.75 | 9.2 |
| 1995 | 6.53 | 3.85 | 5.62 | 0.12 | 19.57 | 3.64 | 1.8 | 3.3 | 1.2 | 7.4 |
| 2000 | 5.33 | 2.82 | 4.78 | 0.7 | 14.7 | 3.09 | 1.6 | 3.4 | 0.8 | 6.2 |
| 2005 | 3.66 | 2.63 | 3 | 0.3 | 14.1 | 2.84 | 1.61 | 2.4 | 0.7 | 6.7 |
| 2010 | 3.1 | 2.08 | 2.6 | 0.1 | 8.25 | 2.37 | 1.57 | 1.9 | 0.6 | 6.6 |
| **Health services coverage** | | | | | | | | | | |
| % of the population with access to fresh water |  |  |  |  |  |  |  |  |  |  |
| 1990 | 80.14 | 21.62 | 89 | 13 | 100 | 84.84 | 11.52 | 89 | 53 | 100 |
| 1995 | 81.18 | 20.92 | 90 | 5 | 100 | 87.75 | 9.39 | 91 | 61 | 100 |
| 2000 | 83.28 | 18.87 | 91 | 22 | 100 | 89.75 | 8.11 | 92 | 61 | 100 |
| 2005 | 85.61 | 17.02 | 93 | 28 | 100 | 91.38 | 7.27 | 93 | 62 | 100 |
| 2010 | 87.86 | 15.43 | 95 | 32 | 100 | 92.88 | 7.18 | 94 | 62 | 100 |
| % of the population with access to sanitation systems |  |  |  |  |  |  |  |  |  |  |
| 1990 | 66.14 | 33.45 | 76 | 2 | 100 | 70.93 | 21.05 | 75 | 19 | 99 |
| 1995 | 66.54 | 32.1 | 77 | 3 | 100 | 75.08 | 18.61 | 80 | 20 | 99 |
| 2000 | 68.43 | 31.26 | 79.5 | 7 | 100 | 77.72 | 17.49 | 81 | 21 | 99 |
| 2005 | 70.72 | 30.45 | 83 | 8 | 100 | 80.21 | 16.45 | 81 | 23 | 99 |
| 2010 | 72.2 | 30.02 | 87 | 9 | 100 | 81.53 | 16.77 | 82 | 24 | 99 |
| % of births attended by qualified personnel |  |  |  |  |  |  |  |  |  |  |
| 1990 | 90.16 | 17.73 | 99 | 31 | 100 | 85.75 | 14.56 | 89 | 66 | 99 |
| 1995 | 84.89 | 23.86 | 98 | 21 | 100 | 87 | 23.52 | 99 | 21 | 100 |
| 2000 | 77.84 | 27.76 | 95 | 6 | 100 | 88.89 | 19.39 | 98 | 24 | 100 |
| 2005 | 87.35 | 22.75 | 99 | 6 | 100 | 97.81 | 3.54 | 100 | 89 | 100 |
| 2010 | 79.94 | 24.31 | 94 | 23 | 100 | 95.12 | 5.51 | 96.5 | 84 | 100 |
| **Health technologies, vaccines, medical products** | | | | | | | | | | |
| % of measles vaccination coverage |  |  |  |  |  |  |  |  |  |  |
| 1990 | 74.45 | 20.69 | 80 | 0 | 99 | 78.93 | 15.15 | 82 | 31 | 99 |
| 1995 | 78.36 | 19.99 | 85 | 0 | 99 | 86.09 | 13.09 | 90 | 47 | 99 |
| 2000 | 81.28 | 18.24 | 88 | 24 | 99 | 90.78 | 8.34 | 92 | 55 | 99 |
| 2005 | 85.02 | 15.8 | 92 | 27 | 99 | 92.24 | 8.38 | 94 | 58 | 99 |
| 2010 | 87.54 | 13.55 | 94 | 39 | 99 | 91.96 | 8.57 | 95 | 58 | 99 |
| % of DPT vaccination coverage |  |  |  |  |  |  |  |  |  |  |
| 1990 | 77.4 | 21.72 | 85 | 0 | 99 | 79.96 | 15.51 | 84 | 41 | 99 |
| 1995 | 79.63 | 21.07 | 88 | 0 | 99 | 85.84 | 12.36 | 87 | 42 | 99 |
| 2000 | 81.71 | 19.32 | 90 | 24 | 99 | 88.15 | 11.09 | 91 | 49 | 99 |
| 2005 | 86.26 | 15.4 | 93 | 25 | 99 | 92.33 | 7.42 | 94 | 60 | 99 |
| 2010 | 88.6 | 12.79 | 94 | 33 | 99 | 92.45 | 7.8 | 95 | 60 | 99 |
| **Financing** | | | | | | | | | | |
| Health expenditure per capita (US$) |  |  |  |  |  |  |  |  |  |  |
| 1995 | 444.3 | 817.56 | 93.23 | 2.37 | 4308.47 | 215.18 | 215.79 | 143.49 | 22.71 | 933.83 |
| 2000 | 454.78 | 814.06 | 100.54 | 3.14 | 4790.01 | 268.88 | 235.43 | 189.41 | 25.88 | 1106.76 |
| 2005 | 751.39 | 1370.86 | 185.3 | 4.72 | 6732.24 | 317.4 | 262.13 | 255.1 | 19.77 | 1394.27 |
| 2010 | 1001.99 | 1713.37 | 309.85 | 11.61 | 8694.29 | 515.2 | 342.76 | 438.11 | 44.93 | 1626.22 |
| Health out-of-pocket expenditure (as a % of the total health expenditure) |  |  |  |  |  |  |  |  |  |  |
| 1995 | 81 | 20.44 | 87.19 | 2.51 | 100 | 80.08 | 20.23 | 87.15 | 19.48 | 100 |
| 2000 | 79.51 | 20.28 | 83.81 | 2.51 | 100 | 79.77 | 17.69 | 86.07 | 31.17 | 100 |
| 2005 | 78.76 | 20.36 | 83.59 | 2.51 | 100 | 79.79 | 17.34 | 82.74 | 30.24 | 100 |
| 2010 | 77.22 | 21.06 | 83.76 | 0.53 | 100 | 79.08 | 18.26 | 85.11 | 27.14 | 100 |
| Public health expenditure |  |  |  |  |  |  |  |  |  |  |
| 1995 | 56.82 | 20.83 | 58.64 | 4.11 | 99.74 | 54.99 | 15.8 | 54.39 | 20.9 | 90.22 |
| 2000 | 56.08 | 20.99 | 54.73 | 4.15 | 99.97 | 55.48 | 15.64 | 53.39 | 27.72 | 90.83 |
| 2005 | 56.75 | 20.51 | 59.19 | 9.01 | 99.9 | 54.06 | 15.57 | 51.2 | 21.29 | 91.95 |
| 2010 | 58.93 | 18.97 | 60.01 | 15.34 | 99.9 | 57.22 | 14.38 | 56.33 | 34.35 | 95.18 |
| **Social determinants** | | | | | | | | | | |
| **Cultural fragmentation** | | | | | | | | | | |
| Linguistic | 0.38 | 0.25 | 0.35 | 0 | 0.93 | 0.18 | 0.2 | 0.11 | 0.017 | 0.63 |
| Ethnic | 0.43 | 0.27 | 0.43 | 0.0021 | 0.92 | 0.4 | 0.17 | 0.42 | 0.095 | 0.739 |
| Religion | 0.43 | 0.22 | 0.46 | 0.0023 | 0.86 | 0.45 | 0.2 | 0.42 | 0.13 | 0.79 |
| **Income inequality** | | | | | | | | | | |
| 1990 | 44.79 | 9.86 | 43.6 | 29.19 | 61.04 | 50.33 | 8.59 | 50.27 | 40.84 | 61.04 |
| 1995 | 44.62 | 10.36 | 44.14 | 28.76 | 60.65 | 51.57 | 6.53 | 50.54 | 42.11 | 60.24 |
| 2000 | 40.36 | 10.8 | 36.03 | 25 | 62.78 | 53.11 | 5.23 | 52.01 | 44.39 | 62.78 |
| 2005 | 41.9 | 8.94 | 41.05 | 27.92 | 59.65 | 51.74 | 4.86 | 51.13 | 40.47 | 59.65 |
| 2010 | 39.95 | 8.92 | 39.37 | 24.24 | 57.49 | 49.09 | 3.69 | 48.14 | 44.49 | 55.91 |
| **Corruption index** | | | | | | | | | | |
| 2007 | 3.9 | 2.08 | 3.3 | 1.4 | 9.4 | 3.79 | 1.55 | 3.4 | 1.6 | 7 |
| 2008 | 4.02 | 2.1 | 3.4 | 1 | 9.3 | 3.93 | 1.67 | 3.5 | 1.4 | 7.1 |
| 2009 | 4.03 | 2.09 | 3.3 | 1.1 | 9.4 | 3.95 | 1.68 | 3.4 | 1.8 | 7.4 |
| 2010 | 4 | 2.08 | 3.3 | 1.1 | 9.3 | 3.77 | 1.6 | 3.4 | 2 | 7.8 |
| 2011 | 4.02 | 2.09 | 3.19 | 0.98 | 9.46 | 3.99 | 1.77 | 3.34 | 1.79 | 7.77 |
| 2012 | 0.43 | 0.19 | 0.37 | 0.08 | 0.9 | 0.43 | 0.16 | 0.38 | 0.19 | 0.76 |
|  | | | | | | | | | | |
| **Index of freedom** | **Free** | **Partially free** | **Not free** |  | | **Free** | **Partially free** | **Not free** |  | |
| 1990 | 63 (39.13%) | 48 (29.8%) | 50 (31.06%) |  |  | 21 (63.6%) | 11 (33.3%) | 1 (3%) |  |  |
| 1995 | 76 (40%) | 62 (32.63%) | 52 (27.37%) |  |  | 17 (51.5%) | 15 (45.4%) | 1 (3%) |  |  |
| 2000 | 86 (45.03%) | 57 (29.84%) | 48 (25.13%) |  |  | 35 (83%) | 6 (14%) | 1 (2%) |  |  |
| 2005 | 88 (46.07%) | 58 (30.37%) | 45 (23.56%) |  |  | 36 (85.7%) | 5 (11.9%) | 1 (2.3%) |  |  |
| 2010 | 87 (44.85%) | 60 (30.93%) | 47 (24.23%) |  |  | 37 (82.2%) | 7 (15%) | 1 (2.2%) |  |  |

| **Variable/indicator** | **AFRICA** | | | | | **ASIA** | | | | |
| --- | --- | --- | --- | --- | --- | --- | --- | --- | --- | --- |
|  | **Average (Interquartile range) 2005** | **DS** | **Median** | **Range** | | **Average (Interquartile range) 2005** | **DS** | **Median** | **Range** | |
| **Results** | | | | | | | | | | |
| **Infant mortality rate <1 year** | | | | | | | | | | |
| 1990 | 90.46 | 35.54 | 92 | 14 | 165 | 50.93 | 33.61 | 42 | 5 | 129 |
| 1995 | 86.11 | 33.06 | 86.5 | 12 | 153 | 44.66 | 29.96 | 37 | 4 | 104 |
| 2000 | 79.36 | 29.4 | 80.5 | 12 | 143 | 37.29 | 25.98 | 30 | 3 | 94 |
| 2005 | 68.78 | 26.86 | 71.5 | 12 | 134 | 29.77 | 21.58 | 21.5 | 2 | 83 |
| 2010 | 58.63 | 24.89 | 61 | 12 | 123 | 24.37 | 18.44 | 18 | 2 | 74 |
| **Infant mortality rate < 5 years** | | | | | | | | | | |
| 1990 | 146.82 | 68.36 | 150.5 | 17 | 326 | 67.79 | 48.14 | 53.5 | 6 | 176 |
| 1995 | 140.07 | 63.88 | 143 | 14 | 279 | 58.56 | 42.04 | 46.5 | 5 | 148 |
| 2000 | 127.46 | 54.11 | 127 | 14 | 234 | 47.85 | 35.6 | 37 | 4 | 134 |
| 2005 | 108.05 | 47.06 | 109.5 | 14 | 216 | 37.29 | 28.8 | 26 | 3 | 118 |
| 2010 | 88.4 | 41.61 | 84.5 | 14 | 193 | 29.89 | 24.16 | 21 | 3 | 104 |
| **Maternal mortality ratio** | | | | | | | | | | |
| 1990 | 816.88 | 480.04 | 720 | 70 | 2300 | 248.14 | 353.53 | 84 | 8 | 1200 |
| 1995 | 762.05 | 467.17 | 660 | 68 | 2400 | 204.06 | 285.35 | 77.5 | 8 | 1200 |
| 2000 | 669.05 | 397.47 | 610 | 28 | 2200 | 159.43 | 219.93 | 64 | 8 | 1100 |
| 2005 | 559.9 | 312.39 | 530 | 35 | 1600 | 117.72 | 151.39 | 53.5 | 6 | 730 |
| 2010 | 466.47 | 262.45 | 450 | 48 | 1200 | 89.81 | 105.94 | 40 | 4 | 500 |
| **Explanatory** | | | | | | | | | | |
| **Health human resources** | | | | | | | | | | |
| **Density of nurses/midwives (per 1,000 inhabitants)** | | | | | | | | | | |
| 1990 |  |  |  |  |  | 0.86 | . | 0.86 | 0.86 | 0.86 |
| 1995 |  |  |  |  |  |  |  |  |  |  |
| 2000 |  |  |  |  |  | 1.56 | 1.09 | 1.02 | 0.85 | 2.83 |
| 2005 | 1.14 | 1.31 | 0.56 | 0.04 | 3.35 | 2.81 | 2.93 | 1.32 | 0.28 | 10.91 |
| 2010 | 0.91 | 0.93 | 0.56 | 0.043 | 3.52 | 3.69 | 2.93 | 3.49 | 0.07 | 11.87 |
| **Density of physicians (per 1,000 inhabitants)** | | | | | | | | | | |
| 1990 | 0.19 | 0.25 | 0.08 | 0.01 | 0.94 | 1.36 | 1.39 | 0.82 | 0.007 | 4.07 |
| 1995 | 0.21 | 0.24 | 0.13 | 0.03 | 0.85 | 1.82 | 1.43 | 1.59 | 0.04 | 3.92 |
| 2000 | 0.32 | 0.67 | 0.094 | 0.02 | 2.11 | 1.73 | 1.33 | 1.3 | 0.16 | 4.21 |
| 2005 | 0.57 | 1.04 | 0.11 | 0.02 | 2.43 | 1.61 | 1.14 | 1.55 | 0.2 | 4 |
| 2010 | 0.27 | 0.53 | 0.089 | 0.008 | 2.83 | 1.81 | 1.49 | 1.53 | 0.023 | 7.73 |
| **Infrastructure** | | | | | | | | | | |
| **Number of beds (per 1,000 inhabitants)** | | | | | | | | | | |
| 1990 | 1.41 | 0.8 | 1.35 | 0.24 | 3.35 | 3.97 | 4.2 | 2.26 | 0.24 | 13.66 |
| 1995 | 0.71 | 0.83 | 0.71 | 0.12 | 1.3 | 6.97 | 4.36 | 7.92 | 0.17 | 15.4 |
| 2000 | 1.4 | 0.28 | 1.4 | 1.2 | 1.6 | 5.5 | 3.64 | 5.47 | 0.7 | 14.7 |
| 2005 | 1.19 | 0.89 | 0.87 | 0.3 | 3 | 3.58 | 3.03 | 2.6 | 0.3 | 14.1 |
| 2010 | 1.49 | 1.42 | 1.4 | 0.1 | 6.3 | 2.22 | 1.73 | 1.8 | 0.4 | 5.9 |
| **Health services coverage** | | | | | | | | | | |
| % of the population with access to fresh water |  |  |  |  |  |  |  |  |  |  |
| 1990 | 57.71 | 21.59 | 54 | 13 | 99 | 81.34 | 17.37 | 85.5 | 22 | 100 |
| 1995 | 61.09 | 20.19 | 59 | 20 | 99 | 80.06 | 20.53 | 86.5 | 5 | 100 |
| 2000 | 64.73 | 18.6 | 63 | 24 | 99 | 82.7 | 17.7 | 88 | 22 | 100 |
| 2005 | 68.57 | 17.72 | 68.5 | 28 | 100 | 86.21 | 14.39 | 89.5 | 40 | 100 |
| 2010 | 72.74 | 17.16 | 73 | 32 | 100 | 88.86 | 12.02 | 92 | 55 | 100 |
| % of the population with access to sanitation systems |  |  |  |  |  |  |  |  |  |  |
| 1990 | 30.54 | 25.45 | 24.5 | 2 | 97 | 70.94 | 31.65 | 84 | 3 | 100 |
| 1995 | 32.88 | 25.59 | 26 | 26 | 97 | 70.13 | 28.95 | 85 | 8 | 100 |
| 2000 | 34.51 | 25.77 | 26 | 7 | 97 | 72.7 | 27.09 | 87 | 16 | 100 |
| 2005 | 36.82 | 26.23 | 27.5 | 8 | 97 | 76.46 | 24.95 | 89 | 25 | 100 |
| 2010 | 38.4 | 25.78 | 29.5 | 9 | 97 | 79.26 | 23.02 | 90.5 | 28 | 100 |
| % of births attended by qualified personnel |  |  |  |  |  |  |  |  |  |  |
| 1990 | 58.75 | 26.66 | 56.5 | 31 | 91 | 90.9 | 18 | 98 | 41 | 100 |
| 1995 | 48.11 | 21.53 | 46 | 21 | 82 | 91.38 | 13.56 | 96 | 50 | 100 |
| 2000 | 55.14 | 23.56 | 58 | 6 | 94 | 74.37 | 31.01 | 95 | 12 | 99 |
| 2005 | 55.3 | 25.31 | 52 | 6 | 99 | 90.47 | 20.43 | 98 | 31 | 100 |
| 2010 | 57 | 17.33 | 59 | 23 | 82 | 77.8 | 27.1 | 88 | 27 | 100 |
| **Health technologies, vaccines, medical products** | | | | | | | | | | |
| % of measles vaccination coverage |  |  |  |  |  |  |  |  |  |  |
| 1990 | 64.6 | 23.35 | 75.5 | 0 | 93 | 76.02 | 19.27 | 80 | 20 | 99 |
| 1995 | 63.63 | 23.59 | 67.5 | 0 | 97 | 82.06 | 15.6 | 86.5 | 40 | 99 |
| 2000 | 63.84 | 20.26 | 69 | 24 | 98 | 85.23 | 16.06 | 92 | 27 | 99 |
| 2005 | 72.61 | 18.01 | 73 | 27 | 99 | 87 | 15.3 | 94 | 41 | 99 |
| 2010 | 80.15 | 15.29 | 83.5 | 46 | 99 | 90.61 | 11.11 | 96 | 62 | 99 |
| % DPT vaccination coverage |  |  |  |  |  |  |  |  |  |  |
| 1990 | 64.78 | 26.94 | 77 | 0 | 99 | 79.63 | 19.84 | 85.5 | 18 | 98 |
| 1995 | 63 | 25.77 | 68.5 | 0 | 97 | 81.6 | 18.37 | 89 | 20 | 99 |
| 2000 | 64.38 | 22.15 | 67.5 | 27 | 98 | 85.15 | 16 | 92 | 24 | 99 |
| 2005 | 74.46 | 19.56 | 79 | 25 | 99 | 87.56 | 13.14 | 94.5 | 49 | 99 |
| 2010 | 81.28 | 16.7 | 85 | 33 | 99 | 90.43 | 10.06 | 94 | 62 | 99 |
| **Financing** | | | | | | | | | | |
| Health expenditure per capita (US$) |  |  |  |  |  |  |  |  |  |  |
| 1995 | 47.75 | 66.84 | 22.64 | 3.8 | 349.6 | 249.48 | 496.79 | 49.41 | 2.37 | 2891.41 |
| 2000 | 46.62 | 66.92 | 19.17 | 5.28 | 371.31 | 256.17 | 487.4 | 52.21 | 3.14 | 2834.21 |
| 2005 | 78.12 | 102.25 | 34.86 | 6.39 | 450.47 | 334.93 | 556.17 | 90.41 | 4.72 | 2928 |
| 2010 | 125.4 | 161.19 | 57.31 | 11.61 | 756.69 | 499.7 | 754.76 | 163.07 | 15.19 | 4115.43 |
| Health out-of-pocket expenditure (as a % of the total health expenditure) |  |  |  |  |  |  |  |  |  |  |
| 1995 | 78.73 | 22 | 84.86 | 21.81 | 100 | 86.71 | 14.08 | 92.57 | 36.07 | 100 |
| 2000 | 77.44 | 22 | 81.32 | 18.15 | 100 | 84.18 | 16.75 | 89.57 | 11.66 | 100 |
| 2005 | 76.04 | 23.65 | 83.82 | 7.28 | 100 | 84.93 | 15.85 | 88.27 | 12.03 | 100 |
| 2010 | 74.35 | 25.09 | 84.56 | 11.93 | 100 | 82.07 | 16.1 | 84.94 | 15.39 | 100 |
| Public health expenditure |  |  |  |  |  |  |  |  |  |  |
| 1995 | 43.81 | 17.58 | 42.33 | 4.11 | 85.16 | 51.13 | 19.25 | 50.5 | 16.98 | 83.96 |
| 2000 | 43.91 | 17.54 | 43.27 | 4.15 | 82.72 | 49.47 | 21.63 | 48.41 | 4.76 | 86.48 |
| 2005 | 47.38 | 17.44 | 49.84 | 16.56 | 92.99 | 48.93 | 21.94 | 50.826 | 9.01 | 85.52 |
| 2010 | 48.98 | 16.62 | 50.15 | 15.34 | 89.6 | 53.54 | 19.99 | 56.01 | 15.58 | 92.19 |
| **Social determinants** | | | | | | | | | | |
| **Cultural fragmentation** | | | | | | | | | | |
| Linguistic | 0.59 | 0.29 | 0.7 | 0.01 | 0.92 | 0.37 | 0.24 | 0.37 | 0.002 | 0.83 |
| Ethnic | 0.62 | 0.25 | 0.71 | 0 | 0.93 | 0.41 | 0.22 | 0.41 | 0.002 | 0.79 |
| Religion | 0.46 | 0.27 | 0.55 | 0.002 | 0.86 | 0.36 | 0.21 | 0.4 | 0.0023 | 0.78 |
| **Income inequality** | | | | | | | | | | |
| 1990 | 40.24 | - | 40.24 | 40.24 | 40.24 | 37.62 | 8 | 38.015 | 29.19 | 45.27 |
| 1995 | 45.85 | 9.9 | 41.66 | 35.33 | 60.65 | 38.89 | 8.38 | 34.96 | 33.2 | 48.52 |
| 2000 | 43.09 | 10.6 | 40.81 | 30 | 58.64 | 40.79 | 6.55 | 42.84 | 33.46 | 46.09 |
| 2005 | 41.12 | 6.5 | 41.45 | 29.83 | 47.68 | 36.9 | 3.74 | 37.51 | 31.18 | 42.56 |
| 2010 | 44.98 | 8.54 | 44.11 | 33.02 | 57.49 | 35.34 | 2.9 | 35.5 | 31.3 | 40.03 |
| **Corruption index** | | | | | | | | | | |
| 2007 | 2.85 | 0.91 | 2.7 | 1.4 | 5.4 | 3.56 | 1.83 | 3 | 1.4 | 9.3 |
| 2008 | 2.86 | 1.06 | 2.75 | 1 | 5.8 | 3.57 | 1.9 | 2.85 | 1.3 | 9.2 |
| 2009 | 2.87 | 1 | 2.8 | 1.1 | 5.6 | 3.62 | 1.93 | 2.7 | 1.3 | 9.2 |
| 2010 | 2.94 | 1 | 2.81 | 0.98 | 6.07 | 3.65 | 1.97 | 2.7 | 1.4 | 9.3 |
| 2011 | 2.94 | 1 | 2.81 | 0.98 | 6.07 | 3.59 | 1.94 | 2.69 | 1 | 9.16 |
| 2012 | 0.33 | 0.11 | 0.33 | 0.08 | 0.65 | 0.37 | 0.18 | 0.33 | 0.08 | 0.87 |
|  | | | | | | | | | | |
| **Index of Freedom** | **Free** | **Partially free** | **Not free** |  | | **Free** | **Partially free** | **Not free** |  | |
| 1990 | 4 (7%) | 19 (37.2%) | 28 (54.9%) |  |  | 5 (12.8%) | 15 (38.46%) | 19 (48.7%) |  |  |
| 1995 | 9 (17.3%) | 20 (38.4%) | 23 (44.23%) |  |  | 4 (8,5%) | 16 (34.04%) | 27 (57.4%) |  |  |
| 2000 | 9 (17.3%) | 25(48.07%) | 18 (34.6%) |  |  | 8 (16.6%) | 13 (27.08%) | 27 (56.25%) |  |  |
| 2005 | 11 (21.15%) | 24 (46.15%) | 17 (32.69%) |  |  | 7 (14.89%) | 16 (34%) | 25 (53.19%) |  |  |
| 2010 | 9 (17.3%) | 23 (44.23%) | 20 (38.46%) |  |  | 7 (14.6%) | 16 (33.3%) | 25 (52.08%) |  |  |

| **Variable/indicator** | **NORTH AMERICA** | | | | | **EUROPE** | | | | |
| --- | --- | --- | --- | --- | --- | --- | --- | --- | --- | --- |
|  | **Average (Interquartile range) 2005** | **DS** | **Median** | **Range** | | **Average (Interquartile range) 2005** | **DS** | **Median** | **Range** | |
| **Results** | | | | | | | | | | |
| **Infant mortality rate <1 year** | | | | | | | | | | |
| 1990 | 8 | 1.41 | 8 | 7 | 9 | 13.3 | 8.03 | 10 | 5 | 37 |
| 1995 | 7 | 1.41 | 7 | 6 | 8 | 11.3 | 8.28 | 8 | 4 | 39 |
| 2000 | 6 | 1.41 | 6 | 5 | 7 | 8.97 | 6.55 | 6 | 3 | 30 |
| 2005 | 6 | 1.41 | 6 | 5 | 7 | 7.11 | 5.17 | 5 | 2 | 23 |
| 2010 | 5.5 | 0.7 | 5.5 | 5 | 6 | 5.6 | 3.94 | 4 | 2 | 19 |
| **Infant mortality rate < 5 years** | | | | | | | | | | |
| 1990 | 9.5 | 2.12 | 9.5 | 8 | 11 | 15.65 | 9.3 | 11 | 6 | 43 |
| 1995 | 8.5 | 2.1 | 8.5 | 7 | 10 | 13.39 | 9.61 | 9 | 5 | 45 |
| 2000 | 7 | 1.4 | 7 | 6 | 8 | 10.58 | 7.56 | 7 | 4 | 34 |
| 2005 | 7 | 1.41 | 7 | 6 | 8 | 8.32 | 5.73 | 6 | 3 | 26 |
| 2010 | 6.5 | 0.7 | 6.5 | 6 | 7 | 6.6 | 4.53 | 5 | 3 | 18 |
| **Maternal mortality ratio** | | | | | | | | | | |
| 1990 | 9 | 4.24 | 9 | 6 | 12 | 22.52 | 28.32 | 12.5 | 6 | 170 |
| 1995 | 9 | 2.82 | 9 | 7 | 11 | 18.87 | 18.16 | 11 | 2 | 72 |
| 2000 | 10 | 4.24 | 10 | 7 | 13 | 15.87 | 13.68 | 11 | 4 | 60 |
| 2005 | 14 | 4.24 | 14 | 11 | 17 | 12.57 | 8.95 | 9.5 | 2 | 48 |
| 2010 | 20 | 9.89 | 20 | 13 | 27 | 11.65 | 9.77 | 8 | 2 | 42 |
| **Explanatory** | | | | | | | | | | |
| **Health human resources** | | | | | | | | | | |
| **Density of nurses/midwives (per 1,000 inhabitants)** | | | | | | | | | | |
| 1990 |  |  |  |  |  | 95.48 | - | 95.48 | 95.48 | 95.48 |
| 1995 |  |  |  |  |  | 14.5 |  | 14.5 | 14.5 | 14.5 |
| 2000 | 9.37 | - | 9.37 | 9.37 | 9.37 | 11.03 |  | 11.03 | 11.03 | 11.03 |
| 2005 | 9.81 | - | 9.81 | 9.81 | 9.81 | 6.38 | 2.28 | 5.9 | 3.6 | 10.36 |
| 2010 | 9.81 | - | 9.81 | 9.81 | 9.81 | 7.27 | 4.91 | 5.96 | 0.23 | 17.48 |
| **Density of physicians (per 1,000 inhabitants)** | | | | | | | | | | |
| 1990 | 1.95 | 0.21 | 1.95 | 1.8 | 2.1 | 4.09 | 7.81 | 2.8 | 1.37 | 47.35 |
| 1995 | 2.05 | 0.07 | 2.05 | 2 | 2.1 | 2.99 | 0.93 | 2.97 | 1.3 | 5.81 |
| 2000 | 2.33 | 0.32 | 2.33 | 2.1 | 2.56 | 3.04 | 0.82 | 3.19 | 1.38 | 4.73 |
| 2005 |  |  |  |  |  | 3.1 | 1.2 | 2.85 | 1.41 | 5 |
| 2010 | 2.24 | 0.25 | 2.24 | 2.06 | 2.42 | 3.3 | 0.94 | 3.34 | 1.15 | 6.16 |
| **Infrastructure** | | | | | | | | | | |
| **Number of beds (per 1,000 inhabitants)** | | | | | | | | | | |
| 1990 | 5.45 | 0.77 | 5.45 | 4.9 | 6 | 8.62 | 3.39 | 7.6 | 4.02 | 16.7 |
| 1995 | 4.55 | 0.63 | 4.55 | 4.1 | 5 | 7.86 | 3.62 | 7.1 | 3.19 | 19.57 |
| 2000 | 3.45 | 0.07 | 3.45 | 3.4 | 3.5 | 6.25 | 2.25 | 5.81 | 3.2 | 12.61 |
| 2005 | 3.3 | 0.14 | 3.3 | 3.2 | 3.4 | 5.85 | 1.99 | 5.55 | 2.7 | 11.1 |
| 2010 | 2.85 | 0.21 | 2.85 | 2.7 | 3 | 5.11 | 1.59 | 5.32 | 2.73 | 8.25 |
| **Health services coverage** | | | | | | | | | | |
| % of the population with access to fresh water |  |  |  |  |  |  |  |  |  |  |
| 1990 | 99.33 | 1.15 | 100 | 98 | 100 | 98 | 5.04 | 100 | 75 | 100 |
| 1995 | 99.66 | 0.57 | 100 | 99 | 100 | 98.12 | 4.14 | 100 | 80 | 100 |
| 2000 | 99.66 | 0.57 | 100 | 99 | 100 | 98.48 | 3.36 | 100 | 84 | 100 |
| 2005 | 99.66 | 0.57 | 100 | 99 | 100 | 98.82 | 2.48 | 100 | 88 | 100 |
| 2010 | 99.66 | 0.57 | 100 | 99 | 100 | 99.32 | 1.28 | 100 | 95 | 100 |
| % of the population with access to sanitation systems |  |  |  |  |  |  |  |  |  |  |
| 1990 | 100 | 0 | 100 | 100 | 100 | 97.2 | 6.5 | 100 | 71 | 100 |
| 1995 | 100 | 0 | 100 | 100 | 100 | 96.7 | 6.76 | 100 | 72 | 100 |
| 2000 | 100 | 0 | 100 | 100 | 100 | 96.02 | 6.79 | 100 | 72 | 100 |
| 2005 | 100 | 0 | 100 | 100 | 100 | 96.29 | 6.47 | 100 | 72 | 100 |
| 2010 | 100 | 0 | 100 | 100 | 100 | 97.73 | 3.79 | 100 | 85 | 100 |
| % of births attended by qualified personnel |  |  |  |  |  |  |  |  |  |  |
| 1990 | - | - | - | - | - | 98.47 | 3.06 | 100 | 89 | 100 |
| 1995 | 98 | - | 98 | 98 | 98 | 98.75 | 3 | 100 | 89 | 100 |
| 2000 | - | - | - | - | - | 99.38 | 1.07 | 100 | 96 | 100 |
| 2005 | - | - | - | - | - | 99.61 | 0.77 | 100 | 98 | 100 |
| 2010 | - | - | - | - | - | 99.66 | 0.5 | 100 | 99 | 100 |
| **Health technology, vaccines, medical products** | | | | | | | | | | |
| % of measles vaccination coverage |  |  |  |  |  |  |  |  |  |  |
| 1990 | 89.5 | 0.7 | 89.5 | 89 | 90 | 82.03 | 20.97 | 87 | 0 | 99 |
| 1995 | 92 | 5.65 | 92 | 88 | 96 | 86.56 | 13.63 | 92 | 50 | 99 |
| 2000 | 93.5 | 3.53 | 93.5 | 91 | 96 | 90.02 | 8.12 | 91.5 | 73 | 99 |
| 2005 | 93 | 1.41 | 93 | 92 | 94 | 93.23 | 5.31 | 96 | 75 | 99 |
| 2010 | 94.5 | 3.53 | 94.5 | 92 | 97 | 92.34 | 8.86 | 95 | 56 | 99 |
| % of DPT vaccination coverage |  |  |  |  |  |  |  |  |  |  |
| 1990 | 89 | 1.41 | 89 | 88 | 90 | 88.77 | 11.57 | 90 | 54 | 99 |
| 1995 | 91 | 5.65 | 91 | 87 | 95 | 92.51 | 8.59 | 96 | 55 | 99 |
| 2000 | 93 | 1.41 | 93 | 92 | 94 | 94.07 | 4.99 | 95 | 80 | 99 |
| 2005 | 95 | 1.41 | 95 | 94 | 96 | 95.46 | 3.45 | 96 | 84 | 99 |
| 2010 | 95 | 0 | 95 | 95 | 95 | 93.54 | 8.41 | 96 | 52 | 99 |
| **Financing** | | | | | | | | | | |
| Health expenditure per capita (US$) |  |  |  |  |  |  |  |  |  |  |
| 1995 | 2804.46 | 1391.47 | 2804.46 | 1820.54 | 3788.38 | 1184.29 | 1182.77 | 784.76 | 25.73 | 4308.48 |
| 2000 | 3440.08 | 1909.08 | 3440.08 | 2090.16 | 4790.01 | 1164.3 | 1117.02 | 830.75 | 23.52 | 3540.86 |
| 2005 | 5092.12 | 2319.47 | 5092.12 | 3452.01 | 6732.24 | 2150.92 | 1935.26 | 1494.02 | 75.97 | 6484.84 |
| 2010 | 6763.57 | 2108 | 6763.57 | 5272.99 | 8254.16 | 2714.95 | 2362.06 | 2043.91 | 190.42 | 8694.29 |
| Health out-of-pocket expenditure (as a % of the total health expenditure) |  |  |  |  |  |  |  |  |  |  |
| 1995 | 40.98 | 20.67 | 40.98 | 26.36 | 55.6 | 84.58 | 18.97 | 93.25 | 33.29 | 100 |
| 2000 | 39.34 | 20.26 | 39.34 | 25.02 | 53.67 | 82.09 | 19.61 | 89.21 | 24.32 | 100 |
| 2005 | 36.42 | 17.94 | 36.42 | 23.73 | 49.11 | 81.01 | 17.57 | 88.02 | 26.84 | 100 |
| 2010 | 35.48 | 18.48 | 35.48 | 22.4 | 48.55 | 80.51 | 17.74 | 88.43 | 32.16 | 100 |
| Public health expenditure |  |  |  |  |  |  |  |  |  |  |
| 1995 | 58.16 | 18.5 | 58.16 | 45.07 | 71.24 | 71.32 | 16.9 | 72.89 | 5.21 | 92.43 |
| 2000 | 56.69 | 19.3 | 56.69 | 43.04 | 70.35 | 69.82 | 15.22 | 72.46 | 17.01 | 90.31 |
| 2005 | 57.23 | 18.38 | 57.23 | 44.23 | 70.23 | 69.89 | 13.9 | 72.68 | 19.17 | 88.19 |
| 2010 | 59.16 | 16.4 | 59.16 | 47.57 | 70.76 | 70.45 | 13.57 | 73.98 | 22.78 | 88.53 |
| **Social determinants** | | | | | | | | | | |
| **Cultural fragmentation** | | | | | | | | | | |
| Linguistic | 0.34 | 0.19 | 0.25 | 0.21 | 0.57 | 0.29 | 0.22 | 0.22 | 0.019 | 0.73 |
| Ethnic | 0.6 | 0.15 | 0.6 | 0.49 | 0.71 | 0.3 | 0.2 | 0.29 | 0.04 | 0.71 |
| Religion | 0.65 | 0.18 | 0.69 | 0.45 | 0.82 | 0.39 | 0.18 | 0.4 | 0.09 | 0.72 |
| **Income inequality** | | | | | | | | | | |
| 1990 | - | - | - | - | - | - | - | - | - | - |
| 1995 | - | - | - | - | - | 32.14 | 3.73 | 31 | 28.76 | 39.29 |
| 2000 | 36.68 | 5.83 | 36.68 | 32.56 | 40.81 | 31.82 | 3.97 | 31.85 | 25 | 41.09 |
| 2005 | - | - | - | - | - | 33.22 | 4.3 | 33.03 | 27.92 | 41.05 |
| 2010 | - | - | - | - | - | 32.02 | 6.86 | 29.62 | 24.24 | 43.56 |
| **Corruption index** | | | | | | | | | | |
| 2007 | 7.95 | 1.06 | 7.95 | 7.2 | 8.7 | 5.85 | 2.26 | 5.3 | 2.1 | 9.4 |
| 2008 | 8 | 0.98 | 8 | 7.3 | 8.7 | 5.83 | 2.15 | 5.5 | 2 | 9.3 |
| 2009 | 8.1 | 0.84 | 8.1 | 7.5 | 8.7 | 5.78 | 2.16 | 5.15 | 2.2 | 9.3 |
| 2010 | 8 | 1.27 | 8 | 7.1 | 8.9 | 5.66 | 2.2 | 5.3 | 2.4 | 9.3 |
| 2011 | 7.9 | 1.08 | 7.9 | 7.13 | 8.67 | 5.64 | 2.26 | 5.48 | 2.29 | 9.4 |
| 2012 | 7.8 | 0.07 | 7.85 | 7.3 | 8.4 | 5.8 | 1.8 | 5.7 | 2.6 | 9 |
|  | | | | | | | | | | |
| **Index of freedom** | **Free** | **Partially free** | **Not free** |  | | **Free** | **Partially free** | **Not free** |  | |
| 1990 | 2 (100%) |  |  |  |  | 22 (88%) | 1 (4%) | 2 (8%) |  |  |
| 1995 | 2 (100%) |  |  |  |  | 33 (78.5%) | 8 (19%) | 1 (2.3%) |  |  |
| 2000 | 2 (100%) |  |  |  |  | 35(83.3%) | 6 (14.3%) | 1 (2.38%) |  |  |
| 2005 | 2 (100%) |  |  |  |  | 36 (85.7%) | 5 (11.9%) | 1 (2.3%) |  |  |
| 2010 | 2 (100%) |  |  |  |  | 37 (82.2%) | 7 (15.5%) | 1 (2%) |  |  |

| **Variable/indicator** | **OCEANIA** | | | | |
| --- | --- | --- | --- | --- | --- |
|  | **Average (Interquartile range) 2005** | **DS** | **Median** | **Range** | |
| **Results** | | | | | |
| **Infant mortality rate <1 year** | | | | | |
| 1990 | 32.75 | 18.82 | 29.5 | 8 | 68 |
| 1995 | 29.33 | 17.81 | 25.5 | 6 | 61 |
| 2000 | 27.08 | 16.99 | 21.5 | 5 | 58 |
| 2005 | 25.16 | 16.39 | 19 | 5 | 56 |
| 2010 | 23.25 | 15.29 | 17.5 | 4 | 51 |
| **Infant mortality rate < 5 years** | | | | | |
| 1990 | 41.58 | 26.84 | 34.5 | 9 | 94 |
| 1995 | 37 | 24.81 | 29.5 | 7 | 83 |
| 2000 | 34.08 | 23.34 | 26 | 6 | 79 |
| 2005 | 31.83 | 21.9 | 23.5 | 6 | 75 |
| 2010 | 29.16 | 20.21 | 22.5 | 5 | 67 |
| **Maternal mortality ratio** | | | | | |
| 1990 | 171.5 | 143.49 | 160 | 7 | 470 |
| 1995 | 143.9 | 113.34 | 125 | 8 | 370 |
| 2000 | 127.3 | 100.3 | 105.5 | 9 | 340 |
| 2005 | 110 | 81.56 | 100 | 6 | 280 |
| 2010 | 97.1 | 69.01 | 95 | 5 | 240 |
| **Explanatory** | | | | | |
| **Health human resources** | | | | | |
| **Density of nurses/midwives (per 1,000 inhabitants)** | | | | | |
| 1990 |  |  |  |  |  |
| 1995 |  |  |  |  |  |
| 2000 | 1.75 | 1.73 | 1.75 | 0.53 | 2.98 |
| 2005 | 1.55 | 0.66 | 1.45 | 0.94 | 2.26 |
| 2010 | 4.07 | 3.15 | 3.31 | 0.45 | 10.87 |
| **Density of physicians (per 1,000 inhabitants)** | | | | | |
| 1990 | 1.09 | 1.11 | 1.04 | 0.07 | 2.2 |
| 1995 | 1.51 | 1.2 | 2 | 0.14 | 2.4 |
| 2000 | 1.19 | 0.9 | 1.08 | 0.05 | 2.5 |
| 2005 | 0.34 | 0.19 | 0.27 | 0.186 | 0.564 |
| 2010 | 0.91 | 1.14 | 0.43 | 0.053 | 3.85 |
| **Infrastructure** | | | | | |
| **Number of beds (per 1,000 inhabitants)** | | | | | |
| 1990 | 3.97 | 2.88 | 4.02 | 0.82 | 8.5 |
| 1995 | 7.45 | 1.76 | 7.45 | 6.2 | 8.7 |
| 2000 | 4.63 | 2.75 | 3.3 | 2.8 | 7.8 |
| 2005 | 2.48 | 1.3 | 2.09 | 0.97 | 4.1 |
| 2010 | 3.08 | 1.3 | 2.7 | 1.4 | 4.8 |
| **Health services coverage** | | | | | |
| % of the population with access to fresh water | | | | | |
| 1990 | 85.33 | 20.24 | 92 | 34 | 100 |
| 1995 | 86.73 | 19.54 | 93 | 34 | 100 |
| 2000 | 88 | 17.38 | 93 | 35 | 100 |
| 2005 | 89.47 | 16.72 | 96 | 37 | 100 |
| 2010 | 90.52 | 16.12 | 97 | 39 | 100 |
| % of the population with access to sanitation systems | | | | | |
| 1990 | 67.28 | 30.02 | 67 | 19 | 100 |
| 1995 | 67.86 | 28.56 | 67 | 20 | 100 |
| 2000 | 68.06 | 28.37 | 74 | 19 | 100 |
| 2005 | 71.12 | 27.9 | 79 | 19 | 100 |
| 2010 | 73.06 | 26.97 | 83 | 19 | 100 |
| % of births attended by qualified personnel | | | | | |
| 1990 | 87.5 | 16.26 | 87.5 | 76 | 99 |
| 1995 | 77 | 30.8 | 89 | 42 | 100 |
| 2000 | 86.8 | 25.67 | 99 | 41 | 100 |
| 2005 | 63 | - | 63 | 63 | 63 |
| 2010 | 99.25 | 0.95 | 99.5 | 98 | 100 |
| **Health technologies, vaccines, medical products** | | | | | |
| % of measles vaccination coverage | | | | | |
| 1990 | 78.66 | 12.95 | 82.5 | 52 | 86 |
| 1995 | 76.5 | 20.52 | 85.5 | 42 | 99 |
| 2000 | 82.91 | 11.19 | 85 | 61 | 95 |
| 2005 | 81.08 | 16.33 | 85.5 | 53 | 99 |
| 2010 | 77 | 21.09 | 84.5 | 39 | 99 |
| % of DPT vaccination coverage | | | | | |
| 1990 | 88.33 | 9.83 | 91 | 68 | 99 |
| 1995 | 81.41 | 14.03 | 84.5 | 60 | 99 |
| 2000 | 82.16 | 17.62 | 90 | 39 | 99 |
| 2005 | 82.91 | 13.6 | 84 | 61 | 99 |
| 2010 | 84.33 | 13.62 | 89 | 56 | 99 |
| **Financing** | | | | | |
| Health expenditure per capita (US$) | | | | | |
| 1995 | 354.27 | 502.26 | 87.21 | 36 | 1564.94 |
| 2000 | 377.9 | 525.3 | 91.55 | 25.91 | 1712.64 |
| 2005 | 619.23 | 967.74 | 167.18 | 32.1 | 3136.48 |
| 2010 | 901.6 | 1532.7 | 201.402 | 56.75 | 5138.11 |
| Out-of-pocket health expenditure (as a % of the total health expenditure) | | | | | |
| 1995 | 67.37 | 25.85 | 71.58 | 2.51 | 100 |
| 2000 | 68.27 | 24.27 | 71.58 | 2.51 | 100 |
| 2005 | 63.89 | 23.58 | 63.48 | 2.51 | 100 |
| 2010 | 62.11 | 24.24 | 62.58 | 0.53 | 100 |
| Public health expenditure | | | | | |
| 1995 | 81.13 | 11.88 | 79.1 | 64.65 | 99.74 |
| 2000 | 81.8 | 12.25 | 81.73 | 58.53 | 99.97 |
| 2005 | 83.8 | 9.54 | 81.38 | 66.88 | 99.9 |
| 2010 | 83.2 | 9.35 | 83.03 | 67.82 | 99.9 |
| **Social determinants** | | | | | |
| **Cultural fragmentation** | | | | | |
| Linguistic | 0.4 | 0.25 | 0.37 | 0.011 | 0.7 |
| Ethnic | 0.26 | 0.22 | 0.15 | 0.04 | 0.7 |
| Religion | 0.6 | 0.13 | 0.62 | 0.25 | 0.82 |
| **Income inequality** | | | | | |
| 1990 | - | - | - | - | - |
| 1995 | - | - | - | - | - |
| 2000 | 61.1 | - | 61.1 | 61.1 | 61.1 |
| 2005 | - | - | - | - | - |
| 2010 | - | - | - | - | - |
| **Corruption index** | | | | | |
| 2007 | 4.42 | 2.95 | 3.2 | 1.7 | 9.4 |
| 2008 | 4.46 | 2.88 | 3 | 2 | 9.3 |
| 2009 | 4.56 | 2.85 | 3.1 | 2.1 | 9.4 |
| 2010 | 4.6 | 2.78 | 3.4 | 2.1 | 9.3 |
| 2011 | 4.6 | 2.86 | 3.33 | 2.16 | 9.46 |
| 2012 | 6.6 | 3.6 | 8.5 | 2.5 | 9 |
|  |  |  |  |  |  |
| **Index of freedom** | **Free** | **Partially free** | **Not free** |  | |
| 1990 | 9 (81.8%) | 2 (18.2%) | - |  |  |
| 1995 | 11 (78.5%) | 3 (21.4%) | - |  |  |
| 2000 | 11 (78.5%) | 3 (21.4%) | - |  |  |
| 2005 | 10 (71.42%) | 4 (28.6%) | - |  |  |
| 2010 | 10 (71.42%) | 4 (28.6%) | - |  |  |
