# Supplementary material for: Determinants of Performance of Health Systems Concerning Maternal and Child Health: A Global Approach
Source: PLoS One. 2015 Mar 30;10(3):e0120747. doi: 10.1371/journal.pone.0120747 (PMC4378969; doi:10.1371/journal.pone.0120747)
Supplement: S3 Table — Bivariate analysis per health result (DOCX) [file pone.0120747.s003.docx]

Supplement 3. Bivariate analysis per health result

| **Variable** | **IMR<5 years** | | | | | **IMR < 1 year** | | | | **MMR** | | | |
| --- | --- | --- | --- | --- | --- | --- | --- | --- | --- | --- | --- | --- | --- |
|  | **Categories** | **Median** | **p25** | **p75** | **p** | **Median** | **p25** | **p75** | **p** | **Median** | **p25** | **p75** | **p** |
| **% of access to fresh water** | Q1 | 109 | 80 | 143 | <0.01 | 72 | 57 | 90 | <0.01 | 535 | 360 | 750 | <0.01 |
|  | Q2 | 33 | 23 | 53.5 |  | 27 | 20 | 41.5 |  | 110 | 67 | 220 |  |
|  | Q3 | 17 | 11 | 23 |  | 14 | 10 | 20 |  | 35 | 22 | 61 |  |
|  | Q4 | 5 | 4 | 7 |  | 4 | 4 | 6 |  | 9 | 6 | 12 |  |
|  | Total | 26 | 11 | 78 |  | 21 | 9 | 55 |  | 75 | 21 | 340 |  |
| **% of access to sanitation systems** | Q1 | 104 | 79 | 138 | <0.01 | 70 | 55 | 86 | <0.01 | 530 | 360 | 730 | <0.01 |
|  | Q2 | 34 | 23 | 60 |  | 28 | 20 | 45 |  | 120 | 71 | 240 |  |
|  | Q3 | 17 | 12 | 24 |  | 15 | 10 | 21 |  | 40 | 23 | 62 |  |
|  | Q4 | 6 | 4 | 8 |  | 5 | 4 | 7 |  | 9 | 7 | 14.5 |  |
|  | Total | 26 | 11 | 78 |  | 22 | 10 | 55 |  | 75 | 21 | 345 |  |
| **% of births attended by health professionals** | Q1 | 93 | 63 | 131 | <0.01 | 63.5 | 49 | 84 | <0.01 | 565 | 355 | 845 | <0.01 |
|  | Q2 | 23 | 17 | 37 |  | 20 | 14 | 31 |  | 91 | 50 | 270 |  |
|  | Q3 | 13 | 8 | 21 |  | 11 | 7 | 18 |  | 36 | 21 | 60 |  |
|  | Q4 | 13 | 9.5 | 17.5 |  | 10.5 | 8.5 | 14.5 |  | 23 | 11 | 35 |  |
|  | Total | 22 | 13 | 60 |  | 19 | 11 | 44 |  | 75 | 31 | 370 |  |
| **Prenatal control coverage – one (1) visit** | Q1 | 83 | 49 | 115 | <0.01 | 59 | 41 | 81 | <0.01 | 425 | 200 | 800 | <0.01 |
|  | Q2 | 41 | 23 | 114 |  | 33 | 20 | 72 |  | 330 | 85 | 610 |  |
|  | Q3 | 22 | 14 | 38 |  | 18 | 12 | 31 |  | 95.5 | 48 | 330 |  |
|  | Q4 | 18.5 | 11.5 | 29.5 |  | 16 | 10 | 25 |  | 55 | 25 | 81 |  |
|  | Total | 33 | 18 | 87 |  | 27 | 16 | 61 |  | 145 | 59 | 540 |  |
| **Prenatal control coverage – four (4) visits** | Q1 | 105 | 72 | 142 | <0.01 | 70 | 54 | 90 | <0.01 | 510 | 370 | 820 | <0.01 |
|  | Q2 | 78 | 37 | 105 |  | 53.5 | 31 | 72 |  | 540 | 200 | 670 |  |
|  | Q3 | 38 | 23 | 103 |  | 31 | 20 | 66 |  | 160 | 73 | 530 |  |
|  | Q4 | 25.5 | 18.5 | 38 |  | 21 | 16 | 30.5 |  | 80.5 | 42 | 120 |  |
|  | Total | 63.5 | 27 | 108.5 |  | 47 | 23 | 71.5 |  | 345 | 80.5 | 605 |  |
| **% of prenatal control** | Q1 | 83 | 52.5 | 115.5 | <0.01 | 60 | 42.5 | 81 | <0.01 | 455 | 210 | 820 | <0.01 |
|  | Q2 | 40 | 23 | 112 |  | 32 | 20 | 72 |  | 330 | 89 | 610 |  |
|  | Q3 | 22 | 14 | 42 |  | 19 | 12 | 34 |  | 100 | 48 | 350 |  |
|  | Q4 | 18 | 12 | 26 |  | 15.5 | 11 | 23 |  | 55 | 25 | 110 |  |
|  | Total | 32.5 | 18 | 87 |  | 27 | 16 | 61 |  | 150 | 59 | 540 |  |
| **Measles vaccination con trol** | Q1 | 99 | 69 | 139 | <0.01 | 68 | 50 | 88 | <0.01 | 76 | 13 | 410 | <0.01 |
|  | Q2 | 26 | 8 | 70.5 |  | 22 | 7 | 52 |  | 80.5 | 23 | 310 |  |
|  | Q3 | 17 | 6 | 27 |  | 15 | 5 | 23 |  | 80 | 13 | 330 |  |
|  | Q4 | 15 | 9 | 24 |  | 13 | 8 | 20.5 |  | 62.5 | 24 | 260 |  |
|  | Total | 24 | 10 | 77 |  | 21 | 9 | 54 |  | 72 | 19 | 330 |  |
| **DPT vaccination coverage** | Q1 | 100 | 66 | 138 | <0.01 | 68 | 48 | 88 | <0.01 | 500 | 335 | 685 | <0.01 |
|  | Q2 | 32 | 14 | 74 |  | 26 | 12 | 54 |  | 91.5 | 54 | 205 |  |
|  | Q3 | 13.5 | 6 | 25 |  | 11.5 | 5 | 21 |  | 41 | 21 | 90 |  |
|  | Q4 | 14 | 7 | 23 |  | 12 | 6 | 20 |  | 9 | 6 | 13.5 |  |
|  | Total | 24 | 11 | 77 |  | 21 | 9 | 54 |  | 72 | 19 | 330 |  |
| **Health out-of-pocket expenditure** | Q1 | 22 | 8 | 79 | 0.02 | 18 | 6 | 54 | <0.01 | 120 | 33 | 370 | <0.01 |
|  | Q2 | 23 | 9 | 66 |  | 19 | 7 | 48 |  | 94 | 50 | 390 |  |
|  | Q3 | 25 | 11 | 70 |  | 21 | 10 | 52 |  | 83 | 24 | 470 |  |
|  | Q4 | 27 | 14 | 76 |  | 23 | 12 | 56 |  | 11 | 7 | 73 |  |
|  | Total | 24 | 10 | 75 |  | 20 | 9 | 53 |  | 72 | 19 | 330 |  |
| **Health expenditure per capita** | Q1 | 100 | 75 | 132 | <0.01 | 68 | 54 | 83 | <0.01 | 290 | 85 | 600 | <0.01 |
|  | Q2 | 33 | 22 | 63 |  | 27 | 19 | 49 |  | 80 | 39 | 410 |  |
|  | Q3 | 17 | 12 | 25 |  | 15 | 10 | 21 |  | 33 | 10 | 120 |  |
|  | Q4 | 6 | 4 | 8 |  | 5 | 4 | 7 |  | 15 | 7 | 89 |  |
|  | Total | 24 | 10 | 75 |  | 20 | 9 | 53 |  | 72 | 19 | 330 |  |
| **Total health expenditure** | Q1 | 33 | 15 | 83 | <0.01 | 27 | 13 | 58 | <0.01 |  |  |  |  |
|  | Q2 | 35 | 19 | 86 |  | 29 | 16 | 59 |  |  |  |  |  |
|  | Q3 | 23 | 10 | 83 |  | 20 | 9 | 57.5 |  |  |  |  |  |
|  | Q4 | 8 | 5 | 27 |  | 7 | 4 | 23 |  |  |  |  |  |
|  | Total | 24 | 10 | 75 |  | 20 | 9 | 53 |  |  |  |  |  |
| **Public health expenditure - % of total expenditure** | Q1 | 73.5 | 34 | 116 | <0.01 | 52.5 | 27 | 77 | <0.01 |  |  |  |  |
|  | Q2 | 28 | 18 | 86 |  | 24 | 15 | 57 |  |  |  |  |  |
|  | Q3 | 15 | 8 | 37 |  | 13 | 7 | 31 |  |  |  |  |  |
|  | Q4 | 10 | 5 | 24 |  | 8 | 4 | 20 |  |  |  |  |  |
|  | Total | 24 | 10 | 75 |  | 20 | 9 | 53 |  |  |  |  |  |
| **Number of physicians per 1,000 inhabitants** | Q1 | 84 | 39 | 121 | <0.01 | 58 | 32 | 79 | <0.01 | 390 | 170 | 540 | <0.01 |
|  | Q2 | 22 | 10 | 38 |  | 18 | 9 | 31 |  | 62 | 27.5 | 120 |  |
|  | Q3 | 9 | 5 | 19 |  | 8 | 4 | 16 |  | 13.5 | 8 | 40 |  |
|  | Q4 | 7 | 5 | 16 |  | 6 | 4 | 14 |  | 10 | 6.5 | 31 |  |
|  | Total | 19 | 7 | 50 |  | 16 | 6 | 41 |  | 42 | 11 | 130 |  |
| **Number of nurses and midwives per 1,000 inhabitants** | Q1 | 83 | 24 | 114 | <0.01 | 55 | 20 | 77 | <0.01 | 325 | 67 | 540 | <0.01 |
|  | Q2 | 33 | 19 | 78 |  | 27.5 | 16 | 54 |  | 120 | 60 | 220 |  |
|  | Q3 | 14 | 8 | 28 |  | 12 | 7 | 25 |  | 26.5 | 10 | 60 |  |
|  | Q4 | 6 | 4 | 14 |  | 5 | 4 | 12 |  | 9.5 | 6.5 | 27 |  |
|  | Total | 21 | 8 | 67 |  | 18 | 7 | 51 |  | 50 | 13 | 200 |  |
| **Number of beds per 1,000 inhabitants** | Q1 | 39 | 24 | 87 | <0.01 | 32 | 20 | 61 | <0.01 | 200 | 77 | 470 | <0.01 |
|  | Q2 | 18 | 11 | 25 |  | 15 | 10 | 21 |  | 47.5 | 19 | 73 |  |
|  | Q3 | 8 | 5 | 21 |  | 7 | 4 | 17 |  | 12 | 8 | 38 |  |
|  | Q4 | 8 | 5 | 18 |  | 7 | 4 | 15 |  | 11.5 | 7 | 32 |  |
|  | Total | 18 | 7 | 33 |  | 15 | 6 | 27 |  | 36 | 10 | 96 |  |
| **Corruption index** | Q1 | 78 | 35 | 111 | <0.01 | 54 | 30 | 74 | <0.01 | 290 | 110 | 600 | <0.01 |
|  | Q2 | 38 | 20 | 81 |  | 31 | 17 | 55 |  | 175 | 51 | 370 |  |
|  | Q3 | 17 | 9 | 23 |  | 15 | 8 | 20 |  | 39 | 14.5 | 83.5 |  |
|  | Q4 | 5 | 4 | 9 |  | 4 | 3.5 | 8 |  | 9 | 6 | 20 |  |
|  | Total | 22 | 9 | 69 |  | 19 | 8 | 51 |  | 73.5 | 15 | 280 |  |
| **Index of freedom** | Not free | 13 | 5 | 25 | <0.01 | 11 | 5 | 22 | <0.01 | 27.5 | 9 | 89 | <0.01 |
|  | Partially free | 41.5 | 21 | 103 |  | 34.5 | 18 | 70 |  | 170 | 57 | 540 |  |
|  | Free | 55 | 20 | 109.5 |  | 44 | 17 | 74 |  | 89 | 40 | 510 |  |
|  | Total | 24 | 10 | 77 |  | 21 | 9 | 54 |  | 73.5 | 21 | 340 |  |
| **Gini** | Q1 | 9 | 5 | 37 | <0.01 | 7.5 | 4 | 31 | <0.01 | 14 | 8 | 59 | <0.01 |
|  | Q2 | 28 | 10 | 72 |  | 23.5 | 8 | 54 |  | 58 | 21 | 320 |  |
|  | Q3 | 40 | 18 | 111 |  | 32 | 16 | 73 |  | 130 | 39 | 530 |  |
|  | Q4 | 49 | 24 | 99 |  | 37.5 | 20 | 64.5 |  | 160 | 85 | 480 |  |
|  | Total | 29 | 11 | 87 |  | 24 | 9 | 60 |  | 79 | 21.5 | 390 |  |
| **Linguistic fragmentation** | Q1 | 20 | 7 | 30 | <0.01 | 17 | 6 | 25 | <0.01 | 47.5 | 11 | 98 | <0.01 |
|  | Q2 | 18 | 9 | 37 |  | 15 | 7 | 30 |  | 36 | 12 | 120 |  |
|  | Q3 | 20 | 9 | 40 |  | 17 | 8 | 33 |  | 48 | 15 | 100 |  |
|  | Q4 | 101 | 67 | 141 |  | 67 | 47 | 88 |  | 500 | 270 | 710 |  |
|  | Total | 25 | 10 | 78 |  | 21 | 9 | 54 |  | 73 | 20 | 330 |  |
| **Ethnica fragmentation** | Q1 | 11 | 5 | 26 | <0.01 | 10 | 4 | 22 | <0.01 | 21 | 8 | 90 | <0.01 |
|  | Q2 | 22 | 11 | 47 |  | 18 | 9 | 40 |  | 49.5 | 21 | 120 |  |
|  | Q3 | 20 | 11 | 34 |  | 17 | 9 | 29 |  | 58 | 24 | 115 |  |
|  | Q4 | 103 | 54 | 140 |  | 67 | 42 | 89 |  | 500 | 250 | 740 |  |
|  | Total | 24 | 10 | 77 |  | 20 | 9 | 54 |  | 72.5 | 19 | 340 |  |
| **Religious fragmentation** | Q1 | 26 | 14 | 67 | <0.01 | 22 | 12 | 51 | <0.01 | 76 | 22 | 290 | <0.01 |
|  | Q2 | 18 | 6 | 40 |  | 15 | 5 | 33 |  | 44 | 11 | 140 |  |
|  | Q3 | 26.5 | 13.5 | 90.5 |  | 22.5 | 12 | 62 |  | 71.5 | 24.5 | 465 |  |
|  | Q4 | 26 | 11 | 101.5 |  | 22.5 | 10 | 64.5 |  | 110 | 26 | 540 |  |
|  | Total | 25 | 10 | 77 |  | 21 | 9 | 54 |  | 73 | 21 | 340 |  |
| **% Primary education in women** | Q1 | - | | | | 10 | 5 | 17 | <0.01 | - | | | |
|  | Q2 | - | | | | 13 | 5 | 23 |  | - | | | |
|  | Q3 | - | | | | 30 | 14.5 | 61 |  | - | | | |
|  | Q4 | - | | | | 60 | 32 | 81.5 |  | - | | | |
|  | Total | - | | | | 21 | 8 | 52 |  | - | | | |
| **% of women employed** | Q1 | - | | | | 21 | 11 | 53 | <0.01 | - | | | |
|  | Q2 | - | | | | 13 | 5 | 25 |  | - | | | |
|  | Q3 | - | | | | 15 | 5 | 30 |  | - | | | |
|  | Q4 | - | | | | 60 | 40 | 83 |  | - | | | |
|  | Total | - | | | | 22 | 9 | 56 |  | - | | | |
| **$2-per-day poverty rate (PPA) (% of the population)** | Q1 | - | | | | 9 | 7 | 13 | <0.01 | - | | | |
|  | Q2 | - | | | | 18 | 14 | 24 |  | - | | | |
|  | Q3 | - | | | | 22 | 19 | 28.5 |  | - | | | |
|  | Q4 | - | | | | 61 | 40 | 81 |  | - | | | |
|  | Total | - | | | | 20 | 13 | 32 |  | - | | | |
| **% of population growth** | Q1 | - | | | | - | | | | 19 | 8 | 37 | <0.01 |
|  | Q2 | - | | | | - | | | | 50 | 15 | 100 |  |
|  | Q3 | - | | | | - | | | | 120 | 44 | 310 |  |
|  | Q4 | - | | | | - | | | | 475 | 170 | 680 |  |
|  | Total | - | | | | - | | | | 73 | 21 | 330 |  |
| **Fertility rate** | Q1 | - | | | | - | | | | 11 | 7 | 26 | <0.01 |
|  | Q2 | - | | | | - | | | | 36 | 16 | 70 |  |
|  | Q3 | - | | | | - | | | | 120 | 63 | 230 |  |
|  | Q4 | - | | | | - | | | | 530 | 370 | 750 |  |
|  | Total | - | | | | - | | | | 73 | 21 | 330 |  |
