# Supplementary material for: Determinants of Performance of Health Systems Concerning Maternal and Child Health: A Global Approach
Source: PLoS One. 2015 Mar 30;10(3):e0120747. doi: 10.1371/journal.pone.0120747 (PMC4378969; doi:10.1371/journal.pone.0120747)
Supplement: S4 Table — Quartile analyse per variable of health system performance and social determinants. (DOCX) [file pone.0120747.s004.docx]

Supplement 4. Quartile analyse per variable of health system performance and social determinants.

| **Region** | **Country** | **% of access to fresh water** | | | **% of access to sanitation systems** | | | **% of births attended by healthcare professionals** | | | **Prenatal control coverage – one (1) visit** | | | **Measles vaccination coverage** | | |
| --- | --- | --- | --- | --- | --- | --- | --- | --- | --- | --- | --- | --- | --- | --- | --- | --- |
|  |  | **2000** | **2005** | **2010** | **2000** | **2005** | **2010** | **2000** | **2005** | **2010** | **2000** | **2005** | **2010** | **2000** | **2005** | **2010** |
| Africa | Sudan | Q1 | Q1 | Q1 | Q1 | Q1 | Q1 |  |  | Q1 | Q1 |  | Q1 | Q1 | Q1 | Q2 |
|  | Comoros | Q3 | Q3 | Q2 | Q1 | Q1 | Q1 | Q2 |  |  | Q2 |  |  | Q1 | Q1 | Q1 |
|  | Tunisia | Q2 | Q2 | Q3 | Q3 | Q3 | Q3 | Q2 |  |  | Q3 |  |  | Q3 | Q3 | Q3 |
|  | Gambia, The | Q2 | Q2 | Q2 | Q2 | Q2 | Q2 | Q1 |  | Q1 | Q3 |  | Q3 | Q3 | Q2 | Q2 |
|  | Guinea-Bissau | Q1 | Q1 | Q1 | Q1 | Q1 | Q1 | Q1 |  | Q1 | Q1 |  | Q2 | Q1 | Q1 | Q1 |
|  | Mauritania | Q1 | Q1 | Q1 | Q1 | Q1 | Q1 |  |  |  |  |  |  | Q1 | Q1 | Q1 |
|  | Egypt, Arab Rep. | Q3 | Q3 | Q3 | Q3 | Q3 | Q3 | Q2 | Q1 |  | Q1 | Q1 |  | Q4 | Q4 | Q3 |
|  | Algeria | Q2 | Q2 | Q2 | Q3 | Q3 | Q3 | Q2 |  |  | Q2 |  |  | Q2 | Q2 | Q3 |
|  | Rwanda | Q1 | Q1 | Q1 | Q2 | Q2 | Q2 | Q1 | Q1 | Q2 | Q3 | Q2 | Q3 | Q2 | Q2 | Q3 |
|  | Morocco | Q2 | Q1 | Q2 | Q2 | Q2 | Q2 |  |  |  |  |  |  | Q3 | Q4 | Q4 |
|  | Central African Republic | Q1 | Q1 | Q1 | Q1 | Q1 | Q1 | Q1 |  | Q1 | Q1 |  | Q1 | Q1 | Q1 | Q1 |
|  | Burundi | Q1 | Q1 | Q1 | Q2 | Q2 | Q1 | Q1 | Q1 | Q1 | Q2 | Q2 | Q4 | Q1 | Q2 | Q2 |
|  | Nigeria | Q1 | Q1 | Q1 | Q1 | Q1 | Q1 |  |  |  |  |  |  | Q1 | Q1 | Q1 |
|  | Botswana | Q3 | Q3 | Q3 | Q2 | Q2 | Q2 | Q2 |  |  | Q4 |  |  | Q3 | Q3 | Q2 |
|  | Cote d'Ivoire | Q2 | Q1 | Q1 | Q1 | Q1 | Q1 | Q2 | Q1 |  | Q3 | Q1 |  | Q1 | Q2 | Q1 |
|  | Angola | Q1 | Q1 | Q1 | Q2 | Q2 | Q2 |  |  |  |  |  |  | Q1 | Q1 | Q2 |
|  | South Africa | Q2 | Q2 | Q2 | Q2 | Q2 | Q2 |  |  |  |  |  |  | Q1 | Q1 | Q1 |
|  | Mali | Q1 | Q1 | Q1 | Q1 | Q1 | Q1 |  |  | Q1 |  |  | Q1 | Q1 | Q1 | Q1 |
|  | Benin | Q1 | Q1 | Q1 | Q1 | Q1 | Q1 |  | Q1 |  |  |  |  | Q1 | Q1 | Q1 |
|  | Congo, Rep. | Q1 | Q1 | Q1 | Q1 | Q1 | Q1 |  | Q1 |  |  | Q1 |  | Q1 | Q1 | Q2 |
|  | Somalia | Q1 | Q1 | Q1 | Q1 | Q1 | Q1 |  |  |  |  |  |  | Q1 | Q1 | Q1 |
|  | Congo, Dem. Rep. | Q1 | Q1 | Q1 | Q1 | Q1 | Q1 |  |  | Q2 |  |  | Q2 | Q1 | Q1 | Q1 |
|  | Liberia | Q1 | Q1 | Q1 | Q1 | Q1 | Q1 | Q1 |  |  | Q2 |  |  | Q1 | Q1 | Q1 |
|  | Ghana | Q1 | Q1 | Q2 | Q1 | Q1 | Q1 |  |  |  |  |  |  | Q3 | Q2 | Q2 |
|  | Cabo Verde | Q2 | Q2 | Q2 | Q2 | Q2 | Q2 |  | Q1 |  |  | Q3 |  | Q2 | Q2 | Q3 |
|  | Zambia | Q1 | Q1 | Q1 | Q1 | Q1 | Q1 |  |  |  |  |  |  | Q2 | Q2 | Q3 |
|  | Mozambique | Q1 | Q1 | Q1 | Q1 | Q1 | Q1 |  |  |  | Q2 |  |  | Q1 | Q2 | Q2 |
|  | Niger | Q1 | Q1 | Q1 | Q1 | Q1 | Q1 | Q1 |  |  | Q1 |  |  | Q1 | Q1 | Q1 |
|  | Cameroon | Q1 | Q1 | Q1 | Q2 | Q1 | Q1 | Q1 |  |  | Q2 |  |  | Q1 | Q1 | Q1 |
|  | Sao Tome and Principe | Q2 | Q2 | Q2 | Q1 | Q1 | Q1 | Q2 |  |  | Q3 |  |  | Q1 | Q2 | Q2 |
|  | Uganda | Q1 | Q1 | Q1 | Q1 | Q1 | Q1 |  |  |  |  |  |  | Q1 | Q1 | Q1 |
|  | Ethiopia | Q1 | Q1 | Q1 | Q1 | Q1 | Q1 | Q1 | Q1 |  | Q1 | Q1 |  | Q1 | Q1 | Q1 |
|  | Gabon | Q2 | Q2 | Q2 | Q1 | Q1 | Q1 | Q2 |  |  | Q3 |  |  | Q1 | Q1 | Q1 |
|  | Chad | Q1 | Q1 | Q1 | Q1 | Q1 | Q1 | Q1 |  | Q1 | Q1 |  | Q1 | Q1 | Q1 | Q1 |
|  | Zimbabwe | Q2 | Q1 | Q1 | Q1 | Q1 | Q1 |  |  |  |  |  |  | Q2 | Q1 | Q2 |
|  | Seychelles | Q3 | Q3 | Q3 | Q3 | Q3 | Q3 |  |  |  |  |  |  | Q4 | Q4 | Q4 |
|  | Namibia | Q2 | Q2 | Q2 | Q1 | Q1 | Q1 | Q2 |  |  | Q3 |  |  | Q1 | Q1 | Q1 |
|  | Guinea | Q1 | Q1 | Q1 | Q1 | Q1 | Q1 |  | Q1 |  |  | Q1 |  | Q1 | Q1 | Q1 |
|  | Malawi | Q1 | Q1 | Q1 | Q1 | Q1 | Q1 | Q1 |  | Q2 | Q3 |  | Q3 | Q1 | Q2 | Q2 |
|  | Mauritius | Q3 | Q4 | Q4 | Q3 | Q3 | Q3 |  | Q2 |  |  |  |  | Q2 | Q4 | Q4 |
|  | Tanzania | Q1 | Q1 | Q1 | Q1 | Q1 | Q1 |  | Q1 | Q1 |  | Q1 | Q2 | Q2 | Q2 | Q2 |
|  | Burkina Faso | Q1 | Q1 | Q1 | Q1 | Q1 | Q1 |  |  | Q2 |  |  | Q2 | Q1 | Q2 | Q2 |
|  | Swaziland | Q1 | Q1 | Q1 | Q2 | Q2 | Q2 | Q2 |  | Q2 | Q3 |  | Q3 | Q3 | Q3 | Q2 |
|  | Lesotho | Q2 | Q1 | Q1 | Q1 | Q1 | Q1 | Q1 |  |  | Q2 |  |  | Q2 | Q2 | Q2 |
|  | Equatorial Guinea | Q1 | Q1 |  | Q3 | Q3 |  | Q2 |  |  | Q3 |  |  | Q1 | Q1 | Q1 |
|  | Togo | Q1 | Q1 | Q1 | Q1 | Q1 | Q1 | Q1 |  | Q1 | Q2 |  | Q1 | Q1 | Q1 | Q1 |
|  | Senegal | Q1 | Q1 | Q1 | Q2 | Q2 | Q2 | Q1 | Q1 |  | Q2 | Q1 |  | Q1 | Q1 | Q2 |
|  | Eritrea | Q1 | Q1 |  | Q1 | Q1 |  |  |  |  |  |  |  | Q2 | Q3 | Q4 |
|  | Djibouti | Q2 | Q2 | Q2 | Q2 | Q2 | Q2 |  |  |  |  |  |  | Q1 | Q1 | Q2 |
|  | Madagascar | Q1 | Q1 | Q1 | Q1 | Q1 | Q1 | Q1 |  |  | Q1 |  |  | Q1 | Q1 | Q1 |
|  | Kenya | Q1 | Q1 | Q1 | Q1 | Q1 | Q1 |  |  |  | Q2 |  |  | Q2 | Q1 | Q2 |
|  | Sierra Leone | Q1 | Q1 | Q1 | Q1 | Q1 | Q1 | Q1 | Q1 | Q1 | Q1 | Q1 | Q2 | Q1 | Q1 | Q1 |
| Latin America and the Caribbean | Virgin Islands (U.S.) | Q4 | Q4 | Q4 | Q3 | Q3 | Q3 |  |  |  |  |  |  |  |  |  |
|  | Brazil | Q3 | Q3 | Q3 | Q2 | Q2 | Q2 |  |  |  |  | Q3 |  | Q4 | Q4 | Q4 |
|  | Puerto Rico | Q3 |  |  | Q4 | Q4 | Q4 |  |  |  |  |  |  |  |  |  |
|  | Jamaica | Q3 | Q2 | Q2 | Q3 | Q2 | Q2 | Q3 | Q2 |  | Q1 | Q2 |  | Q2 | Q2 | Q2 |
|  | Trinidad and Tobago | Q3 | Q2 | Q2 | Q3 | Q3 | Q3 | Q3 |  |  | Q3 |  |  | Q3 | Q3 | Q2 |
|  | Haiti | Q1 | Q1 | Q1 | Q1 | Q1 | Q1 | Q1 |  |  | Q2 |  |  | Q1 | Q1 | Q1 |
|  | Dominica | Q3 | Q3 |  | Q3 | Q2 |  | Q4 | Q2 |  | Q4 | Q4 |  | Q4 | Q4 | Q4 |
|  | Dominican Republic | Q2 | Q2 | Q1 | Q2 | Q2 | Q2 | Q3 |  |  | Q4 |  |  | Q2 | Q2 | Q1 |
|  | St. Kitts and Nevis | Q3 | Q3 | Q3 | Q3 | Q3 |  | Q3 | Q3 |  |  | Q4 |  | Q4 | Q4 | Q4 |
|  | St. Vincent and the Grenadines | Q3 | Q3 | Q2 | Q2 | Q2 |  | Q4 | Q3 |  | Q4 | Q2 |  | Q4 | Q4 | Q4 |
|  | Peru | Q2 | Q2 | Q2 | Q2 | Q2 | Q2 | Q1 |  | Q2 | Q2 |  | Q3 | Q4 | Q3 | Q2 |
|  | Mexico | Q2 | Q2 | Q2 | Q2 | Q2 | Q2 |  |  |  |  |  |  | Q4 | Q3 | Q3 |
|  | Guatemala | Q2 | Q2 | Q2 | Q2 | Q2 | Q2 |  |  |  |  |  |  | Q2 | Q3 | Q2 |
|  | Bolivia | Q2 | Q2 | Q2 | Q1 | Q1 | Q1 | Q2 |  |  | Q2 |  |  | Q2 | Q2 | Q1 |
|  | Paraguay | Q1 | Q2 | Q2 | Q2 | Q2 | Q2 |  |  |  |  |  |  | Q3 | Q2 | Q2 |
|  | St. Lucia | Q3 | Q3 | Q2 | Q2 | Q2 | Q2 | Q4 | Q3 |  | Q1 | Q3 |  | Q2 | Q3 | Q3 |
|  | Ecuador | Q2 | Q2 | Q2 | Q2 | Q2 | Q2 |  |  |  |  |  |  | Q4 | Q3 | Q3 |
|  | Venezuela, RB | Q3 | Q2 |  | Q3 | Q3 |  | Q2 |  |  | Q3 |  |  | Q2 | Q1 | Q1 |
|  | Uruguay | Q3 | Q3 | Q3 | Q3 | Q3 | Q3 |  |  |  |  | Q3 |  | Q3 | Q3 | Q3 |
|  | Suriname | Q2 | Q2 | Q2 | Q3 | Q2 | Q2 | Q2 |  | Q2 | Q3 |  | Q2 | Q2 | Q2 | Q2 |
|  | El Salvador | Q2 | Q2 | Q2 | Q2 | Q2 | Q2 |  |  |  |  |  |  | Q4 | Q4 | Q2 |
|  | Guyana | Q2 | Q2 | Q3 | Q2 | Q2 | Q2 | Q2 | Q2 |  | Q2 | Q3 |  | Q2 | Q2 | Q3 |
|  | Nicaragua | Q2 | Q2 | Q2 | Q2 | Q2 | Q2 |  |  |  |  |  |  | Q2 | Q3 | Q4 |
|  | Costa Rica | Q3 | Q3 | Q3 | Q3 | Q3 | Q3 |  |  | Q3 |  |  |  | Q2 | Q2 | Q2 |
|  | Bahamas, The | Q3 | Q3 | Q3 | Q3 | Q3 | Q3 | Q3 |  |  |  |  |  | Q3 | Q2 | Q2 |
|  | Cuba | Q2 | Q2 | Q2 | Q3 | Q3 | Q3 |  | Q3 | Q3 | Q4 | Q4 | Q4 | Q3 | Q4 | Q4 |
|  | Colombia | Q2 | Q2 | Q2 | Q2 | Q2 | Q2 | Q2 | Q2 | Q3 | Q3 | Q2 | Q3 | Q2 | Q3 | Q2 |
|  | Barbados | Q3 | Q4 | Q4 | Q3 | Q3 |  | Q3 | Q3 |  | Q4 | Q4 |  | Q3 | Q3 | Q2 |
|  | Panama | Q2 | Q2 | Q2 | Q2 | Q2 | Q2 |  | Q2 |  |  |  |  | Q4 | Q4 | Q3 |
|  | Argentina | Q3 | Q3 | Q3 | Q3 | Q3 | Q3 |  | Q2 | Q3 |  | Q3 |  | Q3 | Q4 | Q4 |
|  | Cayman Islands | Q3 | Q3 | Q3 | Q3 | Q3 | Q3 |  | Q3 |  |  |  |  |  |  |  |
|  | Antigua and Barbuda | Q3 | Q3 | Q3 | Q3 | Q3 | Q3 | Q4 | Q3 |  | Q4 | Q4 |  | Q3 | Q4 | Q4 |
|  | Aruba | Q3 | Q3 | Q3 | Q4 | Q4 | Q3 |  |  |  |  |  |  |  |  |  |
|  | Belize | Q2 | Q2 | Q3 | Q3 | Q3 | Q3 | Q4 | Q1 | Q2 | Q4 | Q2 |  | Q4 | Q3 | Q4 |
|  | Grenada | Q3 | Q3 | Q3 | Q4 | Q4 | Q3 | Q4 | Q3 |  | Q2 | Q4 |  | Q3 | Q4 | Q3 |
|  | Chile | Q3 | Q3 | Q3 | Q3 | Q3 | Q3 |  | Q3 | Q3 |  |  |  | Q4 | Q2 | Q2 |
|  | Honduras | Q2 | Q2 | Q2 | Q2 | Q2 | Q2 |  |  |  |  |  |  | Q4 | Q3 | Q4 |
| North America | United States | Q3 | Q3 | Q3 | Q4 | Q4 | Q4 |  |  |  |  |  |  | Q3 | Q2 | Q2 |
|  | Canada | Q4 | Q4 | Q4 | Q4 | Q4 | Q4 |  |  |  |  |  |  | Q4 | Q3 | Q3 |
| Asia | Yemen, Rep. | Q1 | Q1 | Q1 | Q1 | Q2 | Q2 |  |  |  |  |  |  | Q1 | Q1 | Q1 |
|  | Israel | Q4 | Q4 | Q4 | Q4 | Q4 | Q4 |  |  |  |  |  |  | Q4 | Q3 | Q3 |
|  | India | Q2 | Q2 | Q2 | Q1 | Q1 | Q1 | Q1 |  |  | Q1 |  |  | Q1 | Q1 | Q1 |
|  | Cambodia | Q1 | Q1 | Q1 | Q1 | Q1 | Q1 | Q1 | Q1 | Q2 | Q1 | Q1 | Q2 | Q1 | Q2 | Q2 |
|  | Vietnam | Q2 | Q2 | Q2 | Q2 | Q2 | Q2 | Q2 |  |  | Q1 |  |  | Q4 | Q3 | Q4 |
|  | Indonesia | Q2 | Q2 | Q2 | Q2 | Q2 | Q2 | Q2 |  | Q2 | Q3 | Q2 | Q2 | Q2 | Q2 | Q1 |
|  | Pakistan | Q2 | Q2 | Q2 | Q1 | Q1 | Q1 |  | Q1 |  |  | Q1 |  | Q1 | Q2 | Q2 |
|  | Iraq | Q2 | Q2 | Q2 | Q2 | Q2 | Q2 | Q2 |  |  | Q2 |  |  | Q2 | Q1 | Q1 |
|  | Libya | Q1 |  |  | Q3 | Q3 | Q3 |  |  |  |  |  |  | Q3 | Q4 | Q4 |
|  | Nepal | Q2 | Q2 | Q2 | Q1 | Q1 | Q1 | Q1 |  |  | Q1 |  |  | Q1 | Q1 | Q2 |
|  | Syrian Arab Republic | Q2 | Q2 | Q2 | Q3 | Q3 | Q3 |  |  |  | Q1 |  |  | Q2 | Q2 | Q2 |
|  | Uzbekistan | Q2 | Q2 | Q2 | Q3 | Q3 | Q4 | Q3 |  |  | Q4 |  |  | Q4 | Q4 | Q4 |
|  | Qatar | Q4 | Q4 | Q4 | Q4 | Q4 | Q4 |  |  |  |  |  |  | Q3 | Q4 | Q4 |
|  | Philippines | Q2 | Q2 | Q2 | Q2 | Q2 | Q2 | Q1 |  |  | Q3 |  |  | Q2 | Q2 | Q1 |
|  | Kuwait | Q3 | Q3 | Q3 | Q4 | Q4 | Q4 |  |  |  |  |  |  | Q4 | Q4 | Q4 |
|  | Oman | Q2 | Q2 | Q2 | Q3 | Q3 | Q3 | Q2 | Q2 |  | Q4 |  |  | Q4 | Q4 | Q4 |
|  | Lebanon | Q4 | Q4 | Q4 | Q4 | Q4 |  |  |  |  |  |  |  | Q1 | Q1 | Q1 |
|  | Jordan | Q3 | Q3 | Q3 | Q4 | Q4 | Q3 |  |  |  |  |  |  | Q3 | Q3 | Q4 |
|  | Armenia | Q3 | Q3 | Q3 | Q3 | Q3 | Q3 | Q3 | Q2 | Q3 | Q3 | Q2 | Q4 | Q3 | Q3 | Q3 |
|  | Brunei Darussalam |  |  |  |  |  |  |  | Q3 |  |  |  |  | Q4 | Q4 | Q2 |
|  | Singapore | Q4 | Q4 | Q4 | Q4 | Q4 | Q4 |  |  |  |  |  |  | Q4 | Q3 | Q3 |
|  | Bahrain | Q3 | Q4 | Q4 | Q4 | Q4 | Q4 |  | Q2 |  |  |  |  | Q4 | Q4 | Q4 |
|  | Saudi Arabia | Q3 | Q3 | Q3 | Q3 | Q4 | Q4 |  |  |  |  |  |  | Q3 | Q4 | Q4 |
|  | Tajikistan | Q1 | Q1 | Q1 | Q3 | Q3 | Q3 | Q2 | Q1 | Q2 | Q1 | Q1 |  | Q2 | Q2 | Q2 |
|  | United Arab Emirates | Q4 | Q4 | Q4 | Q3 | Q4 | Q3 |  |  |  |  |  |  | Q3 | Q2 | Q2 |
|  | Bangladesh | Q1 | Q1 | Q2 | Q2 | Q2 | Q2 | Q1 |  | Q1 | Q1 |  | Q1 | Q1 | Q3 | Q2 |
|  | Thailand | Q3 | Q3 | Q3 | Q3 | Q3 | Q3 | Q3 |  |  | Q3 |  |  | Q3 | Q3 | Q4 |
|  | Azerbaijan | Q1 | Q1 | Q1 | Q2 | Q2 | Q2 | Q2 |  | Q3 | Q1 |  |  | Q1 | Q1 | Q1 |
|  | Hong Kong SAR, China |  |  |  |  |  |  |  | Q3 |  |  |  |  |  |  |  |
|  | Malaysia | Q3 | Q4 | Q4 | Q3 | Q3 | Q3 | Q3 | Q2 |  |  | Q1 |  | Q2 | Q2 | Q3 |
|  | China | Q2 | Q2 | Q2 | Q2 | Q2 | Q2 | Q3 | Q2 | Q3 | Q3 | Q2 | Q2 | Q2 | Q2 | Q4 |
|  | Turkey | Q3 | Q3 | Q4 | Q3 | Q3 | Q3 |  |  |  |  |  |  | Q2 | Q2 | Q3 |
|  | Macau |  |  |  |  |  |  |  |  |  |  |  |  |  |  |  |
|  | Korea, Rep. | Q3 | Q3 | Q3 | Q4 | Q4 | Q4 |  |  |  |  |  |  | Q3 | Q4 | Q4 |
|  | Burma |  |  |  |  |  |  |  |  |  |  |  |  |  |  |  |
|  | Korea, Dem. Rep. | Q4 | Q3 | Q3 | Q2 | Q2 | Q2 | Q3 |  |  | Q4 |  |  | Q2 | Q3 | Q4 |
|  | Timor-Leste | Q1 | Q1 | Q1 | Q1 | Q1 | Q1 |  |  | Q1 |  |  | Q1 |  | Q1 | Q1 |
|  | Mongolia | Q1 | Q1 | Q1 | Q2 | Q2 | Q2 | Q3 | Q2 | Q3 | Q4 | Q3 | Q4 | Q3 | Q4 | Q3 |
|  | Russian Federation | Q3 | Q3 | Q3 | Q2 | Q2 | Q2 | Q3 | Q2 | Q3 |  |  |  | Q4 | Q4 | Q4 |
|  | West Bank and Gaza | Q3 | Q2 | Q1 | Q3 | Q3 | Q3 | Q3 | Q2 | Q3 | Q4 |  | Q3 |  |  |  |
|  | Japan | Q4 | Q4 | Q4 | Q4 | Q4 | Q4 |  |  |  |  |  |  | Q4 | Q4 | Q2 |
|  | Turkmenistan | Q2 | Q1 | Q1 | Q4 | Q4 | Q4 | Q3 | Q3 |  | Q4 |  |  | Q4 | Q4 | Q4 |
|  | Maldives | Q3 | Q3 | Q3 | Q2 | Q3 | Q3 |  |  |  |  |  |  | Q4 | Q4 | Q3 |
|  | Afghanistan | Q1 | Q1 | Q1 | Q1 | Q1 | Q1 | Q1 |  |  | Q1 |  |  | Q1 | Q1 | Q1 |
|  | Lao PDR | Q1 | Q1 | Q1 | Q1 | Q1 | Q2 |  |  | Q1 |  |  | Q1 | Q1 | Q1 | Q1 |
|  | Sri Lanka | Q2 | Q2 | Q2 | Q2 | Q3 | Q3 | Q3 |  |  | Q4 |  |  | Q4 | Q4 | Q4 |
|  | Iran, Islamic Rep. | Q3 | Q3 | Q3 | Q2 | Q3 | Q3 | Q2 | Q2 |  |  | Q3 |  | Q4 | Q3 | Q4 |
|  | Kyrgyz Republic | Q2 | Q2 | Q2 | Q3 | Q3 | Q3 | Q3 | Q2 |  |  |  |  | Q4 | Q4 | Q4 |
|  | Kazakhstan | Q3 | Q3 | Q2 | Q3 | Q3 | Q3 | Q3 | Q2 | Q3 |  |  | Q4 | Q4 | Q4 | Q4 |
|  | Bhutan | Q2 | Q2 | Q3 | Q1 | Q1 | Q1 | Q1 |  | Q2 | Q1 |  | Q3 | Q2 | Q3 | Q3 |
|  | Myanmar | Q1 | Q1 | Q2 | Q2 | Q2 | Q2 |  |  | Q2 |  |  | Q1 | Q2 | Q1 | Q2 |
| Europe | Ireland | Q4 | Q4 | Q4 | Q4 | Q4 | Q4 |  |  |  |  |  |  | Q2 | Q2 | Q2 |
|  | Belgium | Q4 | Q4 | Q4 | Q4 | Q4 | Q4 |  |  |  |  |  |  | Q2 | Q2 | Q3 |
|  | Sweden | Q4 | Q4 | Q4 | Q4 | Q4 | Q4 |  |  |  |  |  |  | Q3 | Q3 | Q3 |
|  | Croatia | Q3 | Q3 | Q3 | Q4 | Q4 | Q3 | Q4 | Q3 | Q3 | Q4 | Q4 | Q4 | Q3 | Q3 | Q3 |
|  | Switzerland | Q4 | Q4 | Q4 | Q4 | Q4 | Q4 |  |  |  |  |  |  | Q2 | Q2 | Q2 |
|  | Montenegro | Q3 | Q3 | Q3 | Q3 | Q3 | Q3 | Q3 |  |  |  |  |  |  |  | Q2 |
|  | Finland | Q4 | Q4 | Q4 | Q4 | Q4 | Q4 |  |  |  |  |  |  | Q4 | Q4 | Q4 |
|  | Norway | Q4 | Q4 | Q4 | Q4 | Q4 | Q4 |  |  |  |  |  |  | Q2 | Q2 | Q2 |
|  | Georgia | Q2 | Q2 | Q3 | Q3 | Q3 | Q3 | Q3 | Q2 |  |  | Q3 | Q3 | Q1 | Q2 | Q2 |
|  | Ukraine | Q3 | Q3 | Q3 | Q3 | Q3 | Q3 | Q4 | Q3 | Q3 |  | Q3 |  | Q4 | Q3 | Q1 |
|  | Germany | Q4 | Q4 | Q4 | Q4 | Q4 | Q4 |  |  |  |  |  |  | Q3 | Q3 | Q3 |
|  | Bosnia and Herzegovina | Q3 | Q3 | Q3 | Q3 | Q3 | Q3 | Q4 | Q3 |  | Q4 |  |  | Q2 | Q2 | Q2 |
|  | Latvia | Q3 | Q3 | Q3 | Q2 | Q2 |  | Q4 | Q3 |  |  |  |  | Q4 | Q3 | Q2 |
|  | Malta | Q4 | Q4 | Q4 | Q4 | Q4 | Q4 |  |  |  |  |  |  | Q2 | Q2 | Q1 |
|  | Slovenia | Q4 | Q4 | Q4 | Q4 | Q4 | Q4 | Q4 | Q3 | Q3 |  |  |  | Q3 | Q3 | Q3 |
|  | Austria | Q4 | Q4 | Q4 | Q4 | Q4 | Q4 |  |  |  |  |  |  | Q2 | Q1 | Q1 |
|  | Portugal | Q3 | Q3 | Q4 | Q4 | Q4 | Q4 | Q4 |  |  |  |  |  | Q2 | Q3 | Q3 |
|  | Luxembourg | Q4 | Q4 | Q4 | Q4 | Q4 | Q4 |  |  |  |  |  |  | Q3 | Q3 | Q3 |
|  | United Kingdom | Q4 | Q4 | Q4 | Q4 | Q4 | Q4 |  |  |  |  |  |  | Q2 | Q2 | Q2 |
|  | Czech Republic | Q4 | Q4 | Q4 | Q4 | Q4 | Q4 | Q4 | Q3 | Q3 |  |  |  | Q4 | Q4 | Q4 |
|  | Netherlands | Q4 | Q4 | Q4 | Q4 | Q4 | Q4 |  |  |  |  |  |  | Q4 | Q3 | Q3 |
|  | San Marino |  |  |  |  |  |  |  |  |  |  |  |  | Q2 | Q3 | Q1 |
|  | Belarus | Q4 | Q4 | Q4 | Q3 | Q3 | Q3 | Q4 | Q3 |  |  | Q3 |  | Q4 | Q4 | Q4 |
|  | Bulgaria | Q4 | Q4 | Q4 | Q4 | Q4 | Q4 | Q4 | Q2 | Q3 |  |  |  | Q3 | Q3 | Q3 |
|  | Serbia | Q4 | Q3 | Q3 | Q3 | Q3 | Q3 | Q3 |  | Q3 |  |  | Q4 | Q3 | Q3 | Q3 |
|  | Andorra | Q4 | Q4 | Q4 | Q4 | Q4 | Q4 |  |  |  |  |  |  | Q4 | Q3 | Q4 |
|  | Estonia | Q3 | Q3 | Q3 | Q3 | Q3 | Q3 | Q4 | Q3 |  |  |  |  | Q3 | Q3 | Q3 |
|  | Hungary | Q3 | Q4 | Q4 | Q4 | Q4 | Q4 | Q4 | Q3 | Q3 |  |  |  | Q4 | Q4 | Q4 |
|  | Iceland | Q4 | Q4 | Q4 | Q4 | Q4 | Q4 |  |  |  |  |  |  | Q3 | Q2 | Q2 |
|  | Italy | Q4 | Q4 | Q4 |  |  |  |  |  |  |  |  |  | Q2 | Q2 | Q2 |
|  | Poland |  |  |  | Q3 | Q3 |  | Q4 | Q3 |  |  |  |  | Q4 | Q4 | Q4 |
|  | Romania | Q2 | Q2 |  | Q2 | Q2 |  | Q3 | Q2 |  |  |  |  | Q4 | Q4 | Q3 |
|  | France | Q4 | Q4 | Q4 | Q4 | Q4 | Q4 |  |  |  |  |  |  | Q2 | Q2 | Q2 |
|  | Slovak Republic | Q4 | Q4 | Q4 | Q4 | Q4 | Q4 | Q4 | Q3 | Q3 |  |  |  | Q4 | Q4 | Q4 |
|  | Moldova | Q3 | Q3 | Q3 | Q2 | Q2 | Q2 | Q3 | Q3 | Q3 |  | Q3 |  | Q3 | Q4 | Q3 |
|  | Denmark | Q4 | Q4 | Q4 | Q4 | Q4 | Q4 |  |  |  |  |  |  | Q4 | Q3 | Q2 |
|  | Albania | Q3 | Q3 | Q3 | Q3 | Q3 | Q3 | Q3 | Q3 |  | Q4 | Q3 |  | Q3 | Q4 | Q4 |
|  | Lithuania | Q2 | Q2 | Q2 | Q3 | Q3 | Q3 | Q4 | Q3 |  |  |  |  | Q4 | Q4 | Q3 |
|  | Cyprus | Q4 | Q4 | Q4 | Q4 | Q4 | Q4 |  |  |  |  |  |  | Q2 | Q2 | Q2 |
|  | Monaco | Q4 | Q4 | Q4 | Q4 | Q4 | Q4 |  |  |  |  |  |  | Q4 | Q4 | Q4 |
|  | Spain | Q4 | Q4 | Q4 | Q4 | Q4 | Q4 |  |  |  |  |  |  | Q3 | Q4 | Q3 |
|  | Macedonia, Q1YR | Q3 | Q3 | Q3 | Q3 | Q3 | Q3 | Q3 | Q2 |  | Q2 |  |  | Q4 | Q3 | Q4 |
|  | Greece | Q3 | Q4 | Q4 | Q4 | Q4 | Q4 |  |  |  |  |  |  | Q3 | Q3 | Q4 |
| Oceania | Fiji | Q2 | Q3 | Q3 | Q2 | Q2 | Q2 | Q3 |  | Q3 |  |  |  | Q2 | Q2 | Q4 |
|  | Micronesia, Q1ed. Sts. | Q2 | Q2 | Q2 | Q1 | Q1 | Q2 |  |  |  |  |  |  | Q2 | Q3 | Q1 |
|  | Samoa | Q3 | Q3 | Q3 | Q3 | Q3 | Q3 |  |  |  |  |  |  | Q3 | Q1 | Q1 |
|  | Tonga | Q3 | Q3 | Q3 | Q3 | Q3 | Q3 | Q2 |  | Q3 |  |  | Q3 | Q3 | Q4 | Q4 |
|  | Guam | Q4 | Q4 | Q4 | Q3 | Q3 | Q3 |  |  |  |  |  |  |  |  |  |
|  | Vanuatu | Q1 | Q2 | Q2 | Q2 | Q2 | Q2 |  |  |  |  |  |  | Q1 | Q1 | Q1 |
|  | Solomon Islands | Q2 | Q1 | Q1 | Q1 | Q1 | Q1 |  |  |  |  |  |  | Q2 | Q1 | Q1 |
|  | Kiribati | Q1 | Q1 | Q1 | Q1 | Q1 | Q1 |  | Q1 |  |  |  |  | Q2 | Q2 | Q2 |
|  | Palau | Q3 | Q3 | Q2 | Q3 | Q4 | Q4 |  |  | Q3 |  |  | Q2 | Q2 | Q4 | Q1 |
|  | New Caledonia | Q3 | Q3 | Q3 | Q4 | Q4 | Q4 |  |  |  |  |  |  |  |  |  |
|  | American Samoa | Q3 | Q4 | Q4 | Q2 | Q2 | Q2 |  |  |  |  |  |  |  |  |  |
|  | French Polynesia | Q4 | Q4 | Q4 | Q4 | Q4 | Q3 | Q3 |  |  |  |  |  |  |  |  |
|  | Marshall Islands | Q3 | Q3 | Q2 | Q2 | Q2 | Q2 |  |  | Q3 |  |  |  | Q3 | Q2 | Q3 |
|  | New Zealand | Q4 | Q4 | Q4 |  |  |  |  |  |  |  |  |  | Q2 | Q2 | Q2 |
|  | Australia | Q4 | Q4 | Q4 | Q4 | Q4 | Q4 |  |  |  |  |  |  | Q3 | Q3 | Q2 |
|  | Northern Mariana Islands | Q3 | Q3 | Q3 | Q2 | Q2 | Q2 | Q4 |  |  |  |  |  |  |  |  |
|  | Papua New Guinea | Q1 | Q1 | Q1 | Q1 | Q1 | Q1 | Q1 |  |  |  |  |  | Q1 | Q1 | Q1 |
|  | Tuvalu |  |  |  |  |  |  |  |  |  |  |  |  |  |  |  |
|  | Nauru |  |  |  |  |  |  |  |  |  |  |  |  |  |  |  |

| **Region** | **Country** | **Prenatal control coverage – four (4) visit** | | | **Prenatal care coverage** | | | **% of primary education in women** | | | **Out-of-pocket health expenditure** | | | **Health expenditure per capita** | | |
| --- | --- | --- | --- | --- | --- | --- | --- | --- | --- | --- | --- | --- | --- | --- | --- | --- |
|  |  | **2000** | **2005** | **2010** | **2000** | **2005** | **2010** | **2000** | **2005** | **2010** | **2000** | **2005** | **2010** | **2000** | **2005** | **2010** |
| Africa | Sudan |  |  | Q2 | Q1 |  | Q1 |  |  |  | Q3 | Q3 | Q4 | Q1 | Q1 | Q2 |
|  | Comoros |  |  |  | Q2 |  |  | Q2 |  |  | Q4 | Q4 | Q4 | Q1 | Q1 | Q1 |
|  | Tunisia |  |  |  | Q3 |  |  | Q3 | Q2 |  | Q2 | Q3 | Q2 | Q3 | Q2 | Q2 |
|  | Gambia, The |  |  | Q3 | Q3 |  | Q4 | Q3 | Q3 | Q3 | Q1 | Q1 | Q1 | Q2 | Q1 | Q1 |
|  | Guinea-Bissau |  |  | Q3 | Q1 |  | Q2 | Q3 |  | Q3 | Q1 | Q1 | Q1 | Q1 | Q1 | Q1 |
|  | Mauritania | Q1 |  |  |  |  |  | Q3 | Q3 | Q4 | Q3 | Q3 | Q4 | Q1 | Q1 | Q1 |
|  | Egypt, Arab Rep. | Q2 | Q2 |  | Q1 | Q1 |  | Q4 |  |  | Q4 | Q4 | Q4 | Q2 | Q2 | Q2 |
|  | Algeria |  |  |  | Q2 |  |  | Q4 | Q4 |  | Q4 | Q3 | Q4 | Q2 | Q2 | Q2 |
|  | Rwanda | Q1 | Q1 | Q1 | Q3 | Q2 | Q3 |  |  | Q4 | Q1 | Q1 | Q1 | Q1 | Q1 | Q1 |
|  | Morocco |  |  |  |  |  |  | Q4 | Q4 | Q4 | Q2 | Q2 | Q3 | Q2 | Q2 | Q2 |
|  | Central African Republic |  |  | Q1 | Q1 |  | Q1 |  | Q4 | Q4 | Q3 | Q3 | Q3 | Q1 | Q1 | Q1 |
|  | Burundi |  |  | Q1 | Q2 | Q2 | Q4 | Q4 | Q4 | Q3 | Q2 | Q1 | Q2 | Q1 | Q1 | Q1 |
|  | Nigeria | Q2 |  |  |  |  |  | Q4 | Q4 | Q4 | Q3 | Q4 | Q4 | Q1 | Q2 | Q1 |
|  | Botswana |  |  |  | Q4 |  |  | Q3 | Q3 |  | Q1 | Q1 | Q1 | Q3 | Q3 | Q3 |
|  | Cote d'Ivoire | Q2 | Q1 |  | Q3 | Q1 |  | Q4 |  |  | Q2 | Q2 | Q2 | Q2 | Q2 | Q2 |
|  | Angola |  |  |  |  |  |  |  |  | Q4 | Q2 | Q2 | Q2 | Q1 | Q2 | Q2 |
|  | South Africa | Q4 |  |  |  |  |  | Q3 | Q4 |  | Q1 | Q1 | Q1 | Q3 | Q3 | Q3 |
|  | Mali | Q1 |  | Q1 |  |  | Q1 |  | Q4 | Q4 | Q4 | Q4 | Q4 | Q1 | Q1 | Q1 |
|  | Benin | Q3 |  |  |  |  |  |  |  |  | Q4 | Q4 | Q3 | Q1 | Q1 | Q1 |
|  | Congo, Rep. |  | Q3 |  |  | Q1 |  |  | Q4 | Q3 | Q4 | Q4 | Q4 | Q1 | Q1 | Q1 |
|  | Somalia |  |  |  |  |  |  |  |  |  |  |  |  |  |  |  |
|  | Congo, Dem. Rep. |  |  | Q2 |  |  | Q2 |  |  |  | Q2 | Q2 | Q1 | Q1 | Q1 | Q1 |
|  | Liberia |  |  |  | Q2 |  |  |  |  |  | Q1 | Q1 | Q1 | Q1 | Q1 | Q1 |
|  | Ghana | Q3 |  |  |  |  |  | Q4 | Q4 |  | Q1 | Q1 | Q2 | Q1 | Q1 | Q1 |
|  | Cabo Verde | Q3 | Q3 |  |  | Q3 |  |  | Q1 | Q2 | Q3 | Q4 | Q4 | Q2 | Q2 | Q2 |
|  | Zambia | Q4 |  |  |  |  |  | Q4 | Q3 | Q4 | Q2 | Q1 | Q2 | Q1 | Q1 | Q1 |
|  | Mozambique | Q2 |  |  | Q2 |  |  | Q4 | Q4 | Q4 | Q1 | Q1 | Q1 | Q1 | Q1 | Q1 |
|  | Niger | Q1 |  |  | Q1 |  |  | Q4 | Q4 | Q4 | Q3 | Q3 | Q3 | Q1 | Q1 | Q1 |
|  | Cameroon | Q2 |  |  | Q2 |  |  |  |  |  | Q3 | Q4 | Q4 | Q2 | Q1 | Q1 |
|  | Sao Tome and Principe |  |  |  | Q3 |  |  |  |  |  | Q2 | Q2 | Q3 | Q2 | Q2 | Q2 |
|  | Uganda | Q2 |  |  |  |  |  |  |  | Q4 | Q1 | Q1 | Q1 | Q1 | Q1 | Q1 |
|  | Ethiopia | Q1 | Q1 |  | Q1 | Q1 |  | Q4 | Q4 |  | Q2 | Q2 | Q2 | Q1 | Q1 | Q1 |
|  | Gabon | Q3 |  |  | Q4 |  |  |  |  |  | Q3 | Q3 | Q3 | Q3 | Q3 | Q3 |
|  | Chad | Q1 |  | Q1 | Q1 |  | Q1 | Q4 |  |  | Q4 | Q4 | Q4 | Q1 | Q1 | Q1 |
|  | Zimbabwe | Q3 |  |  |  |  |  |  |  |  |  |  |  |  |  |  |
|  | Seychelles |  |  |  |  |  |  | Q1 |  |  | Q4 | Q4 | Q1 | Q3 | Q3 | Q3 |
|  | Namibia | Q3 |  |  | Q3 |  |  | Q2 | Q3 | Q3 | Q1 | Q1 | Q1 | Q3 | Q3 | Q3 |
|  | Guinea | Q2 | Q2 |  |  | Q1 |  | Q4 | Q4 | Q4 | Q4 | Q4 | Q3 | Q1 | Q1 | Q1 |
|  | Malawi | Q3 |  | Q2 | Q3 |  | Q3 |  |  |  | Q1 | Q1 | Q1 | Q1 | Q1 | Q1 |
|  | Mauritius |  |  |  |  |  |  | Q2 | Q1 | Q1 | Q2 | Q3 | Q3 | Q3 | Q3 | Q3 |
|  | Tanzania | Q4 | Q2 | Q1 |  | Q1 | Q2 | Q4 | Q4 |  | Q2 | Q2 | Q1 | Q1 | Q1 | Q1 |
|  | Burkina Faso | Q1 |  | Q1 |  |  | Q3 | Q4 | Q4 | Q4 | Q3 | Q3 | Q2 | Q1 | Q1 | Q1 |
|  | Swaziland |  |  | Q3 | Q3 |  | Q3 | Q3 | Q3 |  | Q1 | Q1 | Q1 | Q2 | Q2 | Q2 |
|  | Lesotho |  |  |  | Q2 |  |  | Q3 | Q3 | Q3 | Q1 | Q1 | Q2 | Q2 | Q2 | Q2 |
|  | Equatorial Guinea |  |  |  | Q3 |  |  | Q2 |  | Q3 | Q2 | Q3 | Q3 | Q2 | Q3 | Q3 |
|  | Togo | Q2 |  | Q2 | Q2 |  | Q1 |  | Q3 |  | Q3 | Q3 | Q3 | Q1 | Q1 | Q1 |
|  | Senegal | Q1 | Q1 |  | Q2 | Q2 |  | Q4 | Q4 | Q4 | Q3 | Q2 | Q2 | Q1 | Q1 | Q1 |
|  | Eritrea | Q2 |  |  |  |  |  | Q4 | Q4 | Q4 | Q4 | Q4 | Q4 | Q1 | Q1 | Q1 |
|  | Djibouti | Q1 |  |  |  |  |  | Q3 | Q3 |  | Q4 | Q4 | Q4 | Q2 | Q2 | Q2 |
|  | Madagascar | Q2 |  |  | Q1 |  |  | Q4 |  |  | Q2 | Q2 | Q3 | Q1 | Q1 | Q1 |
|  | Kenya | Q3 |  |  | Q2 |  |  | Q4 | Q4 |  | Q2 | Q2 | Q2 | Q1 | Q1 | Q1 |
|  | Sierra Leone |  |  | Q3 | Q1 | Q1 | Q2 |  |  |  | Q3 | Q3 | Q3 | Q2 | Q2 | Q1 |
| Latin America and the Caribbean | Virgin Islands (U.S.) |  |  |  |  |  |  |  |  |  |  |  |  |  |  |  |
|  | Brazil |  | Q3 |  |  | Q3 |  |  |  |  | Q1 | Q1 | Q1 | Q3 | Q3 | Q4 |
|  | Puerto Rico |  |  |  |  |  |  |  |  | Q2 |  |  |  |  |  |  |
|  | Jamaica | Q4 |  |  | Q1 | Q2 |  | Q2 |  |  | Q1 | Q1 | Q2 | Q3 | Q2 | Q2 |
|  | Trinidad and Tobago |  |  |  | Q3 |  |  | Q1 | Q2 | Q1 | Q3 | Q2 | Q2 | Q3 | Q4 | Q3 |
|  | Haiti | Q2 |  |  | Q2 |  |  |  |  |  | Q1 | Q2 | Q1 | Q1 | Q1 | Q1 |
|  | Dominica |  |  |  | Q4 | Q4 |  |  | Q1 | Q1 | Q3 | Q2 | Q3 | Q3 | Q3 | Q3 |
|  | Dominican Republic | Q4 |  |  | Q4 |  |  | Q3 | Q3 | Q3 | Q2 | Q2 | Q2 | Q3 | Q3 | Q2 |
|  | St. Kitts and Nevis |  |  |  |  | Q4 |  |  | Q1 | Q1 | Q3 | Q4 | Q3 | Q3 | Q3 | Q3 |
|  | St. Vincent and the Grenadines |  |  |  | Q4 | Q2 |  |  | Q1 |  | Q4 | Q4 | Q4 | Q3 | Q2 | Q2 |
|  | Peru | Q3 |  | Q4 | Q2 |  | Q3 |  | Q1 | Q3 | Q2 | Q2 | Q3 | Q2 | Q2 | Q2 |
|  | Mexico |  |  |  |  |  |  | Q4 | Q4 | Q4 | Q3 | Q3 | Q3 | Q3 | Q3 | Q3 |
|  | Guatemala |  |  |  |  |  |  | Q3 | Q3 | Q3 | Q3 | Q2 | Q2 | Q2 | Q2 | Q2 |
|  | Bolivia | Q2 |  |  | Q2 |  |  | Q3 |  | Q3 | Q2 | Q2 | Q2 | Q2 | Q2 | Q2 |
|  | Paraguay |  |  |  |  |  |  | Q2 | Q3 | Q3 | Q3 | Q3 | Q3 | Q3 | Q2 | Q2 |
|  | St. Lucia |  |  |  | Q1 | Q4 |  | Q1 | Q1 | Q1 | Q4 | Q4 | Q4 | Q3 | Q3 | Q3 |
|  | Ecuador |  |  |  |  |  |  | Q2 |  | Q3 | Q3 | Q4 | Q3 | Q2 | Q3 | Q3 |
|  | Venezuela, RB |  |  |  | Q3 |  |  | Q3 | Q4 | Q4 | Q3 | Q3 | Q4 | Q3 | Q3 | Q3 |
|  | Uruguay |  | Q4 |  |  | Q3 |  |  | Q2 |  | Q1 | Q1 | Q1 | Q4 | Q3 | Q4 |
|  | Suriname |  |  | Q2 | Q3 |  | Q2 |  | Q1 |  | Q1 | Q1 | Q1 | Q3 | Q3 | Q3 |
|  | El Salvador | Q4 |  |  |  |  |  | Q3 | Q3 | Q3 | Q3 | Q3 | Q3 | Q3 | Q3 | Q2 |
|  | Guyana |  |  |  | Q2 | Q3 |  |  |  | Q2 | Q3 | Q3 | Q3 | Q2 | Q2 | Q2 |
|  | Nicaragua | Q4 |  |  |  |  |  | Q3 | Q3 | Q3 | Q3 | Q3 | Q3 | Q2 | Q2 | Q2 |
|  | Costa Rica |  |  |  |  |  |  |  |  |  | Q3 | Q3 | Q3 | Q3 | Q3 | Q3 |
|  | Bahamas, The |  |  |  |  |  |  |  | Q1 |  | Q1 | Q1 | Q1 | Q4 | Q4 | Q4 |
|  | Cuba |  |  | Q4 | Q4 | Q4 | Q4 | Q2 | Q3 | Q1 | Q4 | Q4 | Q4 | Q3 | Q3 | Q3 |
|  | Colombia | Q4 | Q3 | Q4 | Q3 | Q2 | Q3 | Q3 | Q3 | Q4 | Q1 | Q1 | Q2 | Q3 | Q3 | Q3 |
|  | Barbados |  |  |  | Q4 | Q4 |  | Q1 | Q1 | Q1 | Q2 | Q2 | Q2 | Q4 | Q4 | Q4 |
|  | Panama |  |  |  |  |  |  | Q2 | Q2 | Q2 | Q2 | Q2 | Q3 | Q3 | Q3 | Q3 |
|  | Argentina |  | Q4 |  |  | Q4 |  |  |  |  | Q1 | Q1 | Q1 | Q4 | Q3 | Q3 |
|  | Cayman Islands |  |  |  |  |  |  |  |  |  |  |  |  |  |  |  |
|  | Antigua and Barbuda |  |  |  | Q4 | Q4 |  |  |  | Q1 | Q3 | Q3 | Q3 | Q4 | Q4 | Q3 |
|  | Aruba |  |  |  |  |  |  | Q1 | Q1 |  |  |  |  |  |  |  |
|  | Belize |  |  |  | Q4 | Q2 |  | Q1 | Q1 | Q1 | Q2 | Q2 | Q2 | Q3 | Q2 | Q2 |
|  | Grenada |  |  |  | Q3 | Q4 |  | Q1 | Q1 |  | Q4 | Q4 | Q4 | Q3 | Q3 | Q3 |
|  | Chile |  |  |  |  |  |  |  |  | Q3 | Q1 | Q1 | Q1 | Q3 | Q3 | Q4 |
|  | Honduras | Q3 |  |  |  |  |  | Q3 | Q3 | Q3 | Q3 | Q3 | Q4 | Q2 | Q2 | Q2 |
| North America | United States |  |  |  |  |  |  | Q4 | Q4 | Q4 | Q1 | Q1 | Q1 | Q4 | Q4 | Q4 |
|  | Canada |  |  |  |  |  |  |  |  |  | Q1 | Q1 | Q1 | Q4 | Q4 | Q4 |
| Asia | Yemen, Rep. | Q1 |  |  |  |  |  |  | Q4 | Q4 | Q3 | Q4 | Q4 | Q1 | Q1 | Q1 |
|  | Israel |  |  |  |  |  |  | Q2 | Q2 | Q2 | Q1 | Q1 | Q1 | Q4 | Q4 | Q4 |
|  | India | Q1 |  |  | Q1 |  |  | Q4 |  |  | Q3 | Q3 | Q3 | Q1 | Q1 | Q1 |
|  | Cambodia | Q1 | Q1 | Q2 | Q1 | Q1 | Q2 | Q3 |  | Q3 | Q3 | Q2 | Q2 | Q1 | Q1 | Q1 |
|  | Vietnam | Q1 |  |  | Q1 |  |  |  |  |  | Q4 | Q3 | Q3 | Q1 | Q1 | Q2 |
|  | Indonesia | Q4 |  |  | Q3 | Q2 | Q2 |  | Q4 | Q4 | Q2 | Q2 | Q2 | Q1 | Q1 | Q2 |
|  | Pakistan |  |  |  |  | Q1 |  |  | Q4 | Q4 | Q2 | Q3 | Q3 | Q1 | Q1 | Q1 |
|  | Iraq |  |  |  | Q2 |  |  | Q4 |  |  | Q4 | Q4 | Q4 | Q1 | Q2 | Q2 |
|  | Libya |  |  |  |  |  |  |  |  |  | Q4 | Q4 | Q4 | Q3 | Q3 | Q3 |
|  | Nepal | Q1 |  |  | Q1 |  |  | Q4 |  |  | Q3 | Q2 | Q2 | Q1 | Q1 | Q1 |
|  | Syrian Arab Republic |  |  |  | Q1 |  |  |  |  |  | Q4 | Q4 | Q4 | Q2 | Q2 | Q2 |
|  | Uzbekistan |  |  |  | Q4 |  |  |  |  | Q4 | Q4 | Q3 | Q4 | Q2 | Q1 | Q1 |
|  | Qatar |  |  |  |  |  |  | Q1 |  |  | Q4 | Q4 | Q2 | Q4 | Q4 | Q4 |
|  | Philippines | Q4 |  |  | Q3 |  |  |  | Q4 |  | Q2 | Q3 | Q2 | Q2 | Q2 | Q2 |
|  | Kuwait |  |  |  |  |  |  | Q1 | Q1 |  | Q3 | Q3 | Q3 | Q4 | Q4 | Q4 |
|  | Oman |  |  |  | Q4 |  |  | Q2 |  |  | Q1 | Q1 | Q1 | Q3 | Q3 | Q3 |
|  | Lebanon |  |  |  |  |  |  |  | Q3 | Q3 | Q2 | Q2 | Q2 | Q4 | Q3 | Q3 |
|  | Jordan | Q4 |  |  |  |  |  |  |  | Q2 | Q2 | Q3 | Q2 | Q3 | Q3 | Q3 |
|  | Armenia | Q3 | Q2 | Q4 | Q3 | Q2 | Q4 |  | Q2 |  | Q3 | Q4 | Q4 | Q2 | Q2 | Q2 |
|  | Brunei Darussalam |  |  |  |  |  |  |  |  | Q1 | Q4 | Q4 | Q4 | Q4 | Q4 | Q3 |
|  | Singapore |  |  |  |  |  |  |  |  |  | Q4 | Q4 | Q4 | Q4 | Q4 | Q4 |
|  | Bahrain |  |  |  |  |  |  |  |  |  | Q1 | Q1 | Q1 | Q4 | Q4 | Q3 |
|  | Saudi Arabia |  |  |  |  |  |  |  |  | Q4 | Q1 | Q1 | Q1 | Q4 | Q3 | Q3 |
|  | Tajikistan |  |  |  | Q2 | Q1 |  | Q3 | Q2 | Q2 | Q4 | Q4 | Q3 | Q1 | Q1 | Q1 |
|  | United Arab Emirates |  |  |  |  |  |  |  | Q2 | Q2 | Q1 | Q2 | Q1 | Q4 | Q4 | Q4 |
|  | Bangladesh | Q1 |  | Q1 | Q1 |  | Q1 |  | Q4 | Q4 | Q3 | Q4 | Q4 | Q1 | Q1 | Q1 |
|  | Thailand |  |  |  | Q3 |  |  |  |  |  | Q2 | Q2 | Q1 | Q2 | Q2 | Q2 |
|  | Azerbaijan | Q1 |  |  | Q1 |  |  | Q3 | Q3 | Q3 | Q2 | Q3 | Q3 | Q2 | Q2 | Q3 |
|  | Hong Kong SAR, China |  |  |  |  |  |  |  | Q3 | Q2 |  |  |  |  |  |  |
|  | Malaysia |  |  |  |  | Q1 |  | Q3 |  |  | Q2 | Q2 | Q2 | Q3 | Q2 | Q3 |
|  | China |  |  |  | Q3 | Q2 | Q2 |  |  |  | Q4 | Q3 | Q2 | Q2 | Q2 | Q2 |
|  | Turkey | Q2 |  |  |  |  |  |  |  | Q4 | Q2 | Q2 | Q1 | Q3 | Q3 | Q3 |
|  | Macau |  |  |  |  |  |  |  |  |  |  |  |  |  |  |  |
|  | Korea, Rep. |  |  |  |  |  |  |  |  | Q3 | Q2 | Q2 | Q2 | Q4 | Q4 | Q4 |
|  | Burma |  |  |  |  |  |  |  |  |  |  |  |  |  |  |  |
|  | Korea, Dem. Rep. |  |  |  | Q4 |  |  |  |  |  |  |  |  |  |  |  |
|  | Timor-Leste | Q1 |  | Q2 |  |  | Q1 |  |  | Q2 | Q1 | Q1 | Q1 | Q1 | Q1 | Q1 |
|  | Mongolia |  |  | Q3 | Q4 | Q3 | Q4 | Q2 | Q2 | Q1 | Q1 | Q3 | Q4 | Q1 | Q2 | Q2 |
|  | Russian Federation |  |  |  |  |  |  |  |  |  | Q2 | Q2 | Q3 | Q2 | Q3 | Q3 |
|  | West Bank and Gaza |  |  | Q4 | Q4 |  | Q3 | Q2 | Q3 | Q3 |  |  |  |  |  |  |
|  | Japan |  |  |  |  |  |  |  |  |  | Q2 | Q2 | Q2 | Q4 | Q4 | Q4 |
|  | Turkmenistan | Q4 |  |  |  |  |  |  |  |  | Q4 | Q4 | Q4 | Q2 | Q2 | Q2 |
|  | Maldives | Q3 |  |  |  |  |  | Q1 | Q1 |  | Q1 | Q2 | Q2 | Q3 | Q3 | Q3 |
|  | Afghanistan |  |  |  | Q1 |  |  |  |  |  |  | Q3 | Q4 |  | Q1 | Q1 |
|  | Lao PDR |  |  |  |  |  | Q1 | Q3 | Q4 | Q3 | Q3 | Q2 | Q2 | Q1 | Q1 | Q1 |
|  | Sri Lanka |  |  |  | Q4 |  |  |  | Q3 | Q3 | Q2 | Q2 | Q2 | Q2 | Q2 | Q2 |
|  | Iran, Islamic Rep. |  | Q4 |  |  | Q3 |  | Q4 | Q4 |  | Q4 | Q4 | Q3 | Q3 | Q2 | Q3 |
|  | Kyrgyz Republic | Q4 |  |  |  |  |  | Q2 | Q2 | Q2 | Q3 | Q4 | Q3 | Q1 | Q1 | Q1 |
|  | Kazakhstan | Q4 |  | Q3 |  |  | Q4 | Q2 | Q2 | Q2 | Q4 | Q4 | Q4 | Q2 | Q2 | Q3 |
|  | Bhutan |  |  | Q3 | Q1 |  | Q3 | Q3 | Q2 | Q2 | Q4 | Q4 | Q4 | Q2 | Q2 | Q2 |
|  | Myanmar | Q3 |  |  |  |  | Q1 |  |  |  | Q4 | Q4 | Q3 | Q1 | Q1 | Q1 |
| Europe | Ireland |  |  |  |  |  |  | Q1 |  | Q1 | Q1 | Q1 | Q1 | Q4 | Q4 | Q4 |
|  | Belgium |  |  |  |  |  |  | Q1 | Q2 | Q2 | Q2 | Q2 | Q2 | Q4 | Q4 | Q4 |
|  | Sweden |  |  |  |  |  |  | Q2 | Q2 | Q2 | Q3 | Q3 | Q3 | Q4 | Q4 | Q4 |
|  | Croatia |  |  |  | Q4 | Q4 | Q4 | Q2 | Q1 | Q2 | Q4 | Q4 | Q4 | Q3 | Q4 | Q4 |
|  | Switzerland |  |  |  |  |  |  | Q1 | Q2 | Q1 | Q2 | Q2 | Q2 | Q4 | Q4 | Q4 |
|  | Montenegro |  |  |  |  |  |  |  | Q1 |  | Q3 | Q3 | Q3 | Q3 | Q3 | Q3 |
|  | Finland |  |  |  |  |  |  |  | Q2 | Q2 | Q2 | Q2 | Q2 | Q4 | Q4 | Q4 |
|  | Norway |  |  |  |  |  |  | Q1 | Q1 | Q2 | Q3 | Q3 | Q3 | Q4 | Q4 | Q4 |
|  | Georgia |  | Q3 | Q4 |  | Q3 | Q3 |  | Q3 |  | Q4 | Q4 | Q3 | Q2 | Q2 | Q2 |
|  | Ukraine |  |  |  |  | Q3 |  |  | Q3 | Q3 | Q3 | Q3 | Q4 | Q2 | Q2 | Q2 |
|  | Germany |  |  |  |  |  |  |  | Q3 |  | Q1 | Q1 | Q1 | Q4 | Q4 | Q4 |
|  | Bosnia and Herzegovina |  |  |  | Q4 |  |  |  |  |  | Q4 | Q4 | Q4 | Q3 | Q3 | Q3 |
|  | Latvia |  |  |  |  |  |  |  |  | Q1 | Q4 | Q4 | Q3 | Q3 | Q3 | Q3 |
|  | Malta |  |  |  |  |  |  |  |  |  | Q4 | Q3 | Q4 | Q4 | Q4 | Q4 |
|  | Slovenia |  |  |  |  |  |  | Q1 | Q1 | Q1 | Q1 | Q1 | Q1 | Q4 | Q4 | Q4 |
|  | Austria |  |  |  |  |  |  |  |  |  | Q1 | Q1 | Q1 | Q4 | Q4 | Q4 |
|  | Portugal |  |  |  |  |  |  |  | Q2 | Q1 | Q2 | Q2 | Q2 | Q4 | Q4 | Q4 |
|  | Luxembourg |  |  |  |  |  |  | Q1 | Q1 | Q1 | Q2 | Q2 | Q2 | Q4 | Q4 | Q4 |
|  | United Kingdom |  |  |  |  |  |  | Q1 | Q1 | Q2 | Q1 | Q1 | Q1 | Q4 | Q4 | Q4 |
|  | Czech Republic |  |  |  |  |  |  |  |  |  | Q4 | Q3 | Q3 | Q3 | Q4 | Q4 |
|  | Netherlands |  |  |  |  |  |  | Q2 | Q3 |  | Q1 | Q1 | Q1 | Q4 | Q4 | Q4 |
|  | San Marino |  |  |  |  |  |  |  |  |  | Q3 | Q4 | Q4 | Q4 | Q4 | Q4 |
|  | Belarus |  |  |  |  | Q4 |  |  |  | Q2 | Q1 | Q2 | Q3 | Q2 | Q3 | Q3 |
|  | Bulgaria |  |  |  |  |  |  | Q2 | Q2 | Q1 | Q4 | Q4 | Q4 | Q2 | Q3 | Q3 |
|  | Serbia |  |  | Q4 |  |  | Q4 |  |  | Q2 | Q3 | Q3 | Q4 | Q2 | Q3 | Q3 |
|  | Andorra |  |  |  |  |  |  |  |  |  | Q2 | Q2 | Q2 | Q4 | Q4 | Q4 |
|  | Estonia |  |  |  |  |  |  | Q1 | Q1 | Q1 | Q3 | Q3 | Q3 | Q3 | Q3 | Q4 |
|  | Hungary |  |  |  |  |  |  | Q2 | Q2 | Q2 | Q3 | Q2 | Q2 | Q3 | Q4 | Q4 |
|  | Iceland |  |  |  |  |  |  |  | Q1 | Q1 | Q4 | Q3 | Q3 | Q4 | Q4 | Q4 |
|  | Italy | Q3 |  |  |  |  |  | Q2 | Q2 | Q2 | Q4 | Q3 | Q3 | Q4 | Q4 | Q4 |
|  | Poland |  |  |  |  |  |  | Q3 | Q3 | Q3 | Q4 | Q3 | Q2 | Q3 | Q3 | Q4 |
|  | Romania | Q3 |  |  |  |  |  | Q3 | Q2 |  | Q4 | Q4 | Q4 | Q2 | Q3 | Q3 |
|  | France |  |  |  |  |  |  | Q2 | Q2 | Q2 | Q1 | Q1 | Q1 | Q4 | Q4 | Q4 |
|  | Slovak Republic |  |  |  |  |  |  |  |  |  | Q3 | Q3 | Q2 | Q3 | Q4 | Q4 |
|  | Moldova | Q4 | Q4 |  |  | Q3 |  | Q2 | Q2 | Q2 | Q2 | Q2 | Q2 | Q1 | Q2 | Q2 |
|  | Denmark |  |  |  |  |  |  | Q1 | Q2 | Q2 | Q3 | Q3 | Q3 | Q4 | Q4 | Q4 |
|  | Albania | Q2 |  |  | Q4 | Q3 |  | Q2 |  |  | Q4 | Q3 | Q4 | Q2 | Q2 | Q2 |
|  | Lithuania |  |  |  |  |  |  | Q1 | Q2 | Q1 | Q3 | Q4 | Q4 | Q3 | Q3 | Q3 |
|  | Cyprus |  |  |  |  |  |  | Q1 | Q1 | Q1 | Q4 | Q2 | Q3 | Q4 | Q4 | Q4 |
|  | Monaco |  |  |  |  |  |  |  |  |  | Q1 | Q1 | Q1 | Q4 | Q4 | Q4 |
|  | Spain |  |  |  |  |  |  | Q1 | Q2 | Q2 | Q2 | Q2 | Q2 | Q4 | Q4 | Q4 |
|  | Macedonia, Q1YR |  |  |  | Q2 |  |  | Q2 | Q2 | Q1 | Q4 | Q4 | Q4 | Q3 | Q3 | Q2 |
|  | Greece |  |  |  |  |  |  | Q2 | Q1 | Q1 | Q3 | Q3 | Q3 | Q4 | Q4 | Q4 |
| Oceania | Fiji |  |  |  |  |  |  | Q1 |  |  | Q1 | Q1 | Q2 | Q2 | Q2 | Q2 |
|  | Micronesia, Q1ed. Sts. |  |  |  |  |  |  |  |  |  | Q4 | Q4 | Q4 | Q3 | Q3 | Q3 |
|  | Samoa |  |  |  |  |  |  | Q1 |  | Q1 | Q2 | Q1 | Q1 | Q2 | Q2 | Q2 |
|  | Tonga |  |  |  |  |  | Q3 |  |  |  | Q2 | Q1 | Q2 | Q2 | Q2 | Q2 |
|  | Guam |  |  |  |  |  |  |  |  |  |  |  |  |  |  |  |
|  | Vanuatu |  |  |  |  |  |  |  |  |  | Q2 | Q2 | Q1 | Q2 | Q2 | Q2 |
|  | Solomon Islands |  |  |  |  |  |  |  | Q2 |  | Q1 | Q1 | Q1 | Q2 | Q2 | Q2 |
|  | Kiribati |  |  |  |  |  |  |  |  |  | Q1 | Q1 | Q1 | Q2 | Q2 | Q2 |
|  | Palau |  |  | Q3 |  |  | Q2 |  |  |  | Q2 | Q1 | Q1 | Q4 | Q4 | Q4 |
|  | New Caledonia |  |  |  |  |  |  |  |  |  |  |  |  |  |  |  |
|  | American Samoa |  |  |  |  |  |  |  |  |  |  |  |  |  |  |  |
|  | French Polynesia |  |  |  |  |  |  |  |  |  |  |  |  |  |  |  |
|  | Marshall Islands |  |  |  |  |  |  |  |  |  | Q2 | Q2 | Q2 | Q4 | Q3 | Q3 |
|  | New Zealand |  |  |  |  |  |  | Q1 | Q1 | Q1 | Q1 | Q1 | Q1 | Q4 | Q4 | Q4 |
|  | Australia |  |  |  |  |  |  | Q3 | Q3 | Q3 | Q1 | Q1 | Q1 | Q4 | Q4 | Q4 |
|  | Northern Mariana Islands |  |  |  |  |  |  |  |  |  |  |  |  |  |  |  |
|  | Papua New Guinea |  |  |  |  |  |  |  |  |  | Q1 | Q1 | Q1 | Q2 | Q1 | Q1 |
|  | Tuvalu |  |  |  |  |  |  |  |  |  | Q4 | Q4 | Q4 | Q3 | Q3 | Q3 |
|  | Nauru |  |  |  |  |  |  |  |  |  |  |  |  |  |  |  |

| **Region** | **Country** | **Health total expenditure** | | | **Public health expenditure - % of total expenditure** | | | **Number of beds per 1,000 inhabitans** | | | **Number of nurses per 1,000 inhabitans** | | | **Number of physicians per 1,000 inhabitants** | | |
| --- | --- | --- | --- | --- | --- | --- | --- | --- | --- | --- | --- | --- | --- | --- | --- | --- |
|  |  | **2000** | **2005** | **2010** | **2000** | **2005** | **2010** | **2000** | **2005** | **2010** | **2000** | **2005** | **2010** | **2000** | **2005** | **2010** |
| Africa | Sudan | Q1 | Q1 | Q2 | Q1 | Q1 | Q1 |  |  |  |  |  | Q2 | Q1 |  | Q1 |
|  | Comoros | Q1 | Q1 | Q1 | Q2 | Q2 | Q1 |  |  |  |  |  |  |  |  |  |
|  | Tunisia | Q2 | Q2 | Q3 | Q3 | Q2 | Q2 |  |  | Q2 |  |  | Q3 |  |  | Q2 |
|  | Gambia, The | Q2 | Q2 | Q1 | Q1 | Q2 | Q2 |  | Q1 |  |  |  | Q1 |  |  | Q1 |
|  | Guinea-Bissau | Q2 | Q2 | Q3 | Q1 | Q1 | Q1 |  |  |  |  |  | Q1 |  |  | Q1 |
|  | Mauritania | Q3 | Q2 | Q2 | Q3 | Q3 | Q3 |  |  |  |  |  | Q1 |  |  | Q1 |
|  | Egypt, Arab Rep. | Q2 | Q2 | Q2 | Q1 | Q1 | Q1 |  | Q2 | Q2 |  | Q3 | Q3 | Q3 | Q3 | Q3 |
|  | Algeria | Q1 | Q1 | Q1 | Q3 | Q3 | Q4 |  |  |  |  |  | Q2 |  |  | Q2 |
|  | Rwanda | Q1 | Q3 | Q4 | Q1 | Q2 | Q2 |  |  |  |  | Q1 | Q1 |  | Q1 | Q1 |
|  | Morocco | Q1 | Q2 | Q2 | Q1 | Q1 | Q1 |  |  |  |  |  | Q2 |  |  | Q2 |
|  | Central African Republic | Q1 | Q1 | Q1 | Q2 | Q2 | Q2 |  |  |  |  |  |  |  |  |  |
|  | Burundi | Q3 | Q4 | Q4 | Q1 | Q1 | Q3 |  |  |  |  |  |  | Q1 |  |  |
|  | Nigeria | Q2 | Q3 | Q2 | Q1 | Q1 | Q1 | Q1 |  |  |  |  | Q2 | Q1 |  | Q2 |
|  | Botswana | Q2 | Q2 | Q2 | Q3 | Q3 | Q3 |  |  | Q2 |  |  | Q2 |  |  | Q2 |
|  | Cote d'Ivoire | Q3 | Q2 | Q3 | Q1 | Q1 | Q1 |  |  |  |  |  | Q1 |  |  | Q1 |
|  | Angola | Q1 | Q1 | Q1 | Q2 | Q2 | Q3 |  | Q1 |  |  |  |  |  |  |  |
|  | South Africa | Q4 | Q4 | Q4 | Q1 | Q1 | Q2 |  | Q2 |  |  |  |  |  |  |  |
|  | Mali | Q3 | Q3 | Q3 | Q1 | Q2 | Q1 |  | Q1 | Q1 |  |  | Q1 | Q1 |  | Q1 |
|  | Benin | Q2 | Q2 | Q1 | Q2 | Q2 | Q2 |  | Q1 | Q1 |  |  | Q2 |  |  | Q1 |
|  | Congo, Rep. | Q1 | Q1 | Q1 | Q3 | Q3 | Q3 |  | Q1 |  |  |  | Q2 |  |  | Q1 |
|  | Somalia |  |  |  |  |  |  |  |  |  |  |  | Q1 |  |  | Q1 |
|  | Congo, Dem. Rep. | Q2 | Q2 | Q3 | Q1 | Q1 | Q2 |  | Q1 |  |  |  |  |  |  |  |
|  | Liberia | Q3 | Q3 | Q4 | Q1 | Q1 | Q1 |  |  | Q1 |  |  | Q1 |  |  | Q1 |
|  | Ghana | Q2 | Q3 | Q2 | Q2 | Q3 | Q2 |  | Q1 |  |  |  | Q2 |  |  | Q1 |
|  | Cabo Verde | Q2 | Q2 | Q1 | Q3 | Q4 | Q4 |  | Q2 | Q2 |  |  | Q1 |  |  | Q2 |
|  | Zambia | Q3 | Q3 | Q2 | Q2 | Q2 | Q3 |  |  | Q2 |  |  | Q2 |  |  | Q1 |
|  | Mozambique | Q3 | Q3 | Q2 | Q3 | Q3 | Q2 |  |  |  |  |  | Q1 | Q1 |  | Q1 |
|  | Niger | Q3 | Q3 | Q3 | Q1 | Q1 | Q1 |  | Q1 |  |  |  | Q1 |  |  | Q1 |
|  | Cameroon | Q2 | Q2 | Q2 | Q1 | Q1 | Q1 |  |  | Q1 |  |  |  |  |  |  |
|  | Sao Tome and Principe | Q4 | Q4 | Q3 | Q2 | Q2 | Q1 |  |  |  |  |  |  |  |  |  |
|  | Uganda | Q3 | Q4 | Q4 | Q1 | Q1 | Q1 |  | Q1 | Q1 |  | Q2 | Q2 |  | Q1 | Q1 |
|  | Ethiopia | Q1 | Q1 | Q1 | Q2 | Q3 | Q2 |  |  |  |  |  | Q1 | Q1 |  | Q1 |
|  | Gabon | Q1 | Q1 | Q1 | Q1 | Q1 | Q2 |  |  | Q4 |  |  |  |  |  |  |
|  | Chad | Q3 | Q2 | Q1 | Q2 | Q1 | Q1 |  | Q1 |  |  |  |  |  |  |  |
|  | Zimbabwe |  |  |  |  |  |  |  |  |  |  |  |  |  |  |  |
|  | Seychelles | Q2 | Q1 | Q1 | Q4 | Q4 | Q4 |  |  |  |  |  |  |  |  |  |
|  | Namibia | Q3 | Q3 | Q3 | Q3 | Q2 | Q2 |  |  |  |  |  | Q2 |  |  | Q2 |
|  | Guinea | Q3 | Q2 | Q2 | Q1 | Q1 | Q1 |  | Q1 |  |  | Q1 | Q1 | Q1 | Q1 | Q1 |
|  | Malawi | Q3 | Q4 | Q3 | Q2 | Q4 | Q3 |  |  |  |  |  | Q1 |  |  | Q1 |
|  | Mauritius | Q1 | Q2 | Q2 | Q2 | Q2 | Q2 |  | Q2 |  |  |  |  |  |  |  |
|  | Tanzania | Q1 | Q1 | Q3 | Q2 | Q2 | Q1 |  |  | Q1 |  |  | Q1 |  |  | Q1 |
|  | Burkina Faso | Q2 | Q3 | Q3 | Q1 | Q3 | Q2 |  |  | Q1 |  |  | Q1 |  |  | Q1 |
|  | Swaziland | Q2 | Q3 | Q3 | Q3 | Q3 | Q3 |  |  |  |  |  |  | Q1 |  |  |
|  | Lesotho | Q3 | Q3 | Q4 | Q2 | Q2 | Q4 |  |  |  |  |  |  |  |  |  |
|  | Equatorial Guinea | Q1 | Q1 | Q1 | Q4 | Q2 | Q3 |  | Q2 | Q2 |  |  |  |  |  |  |
|  | Togo | Q2 | Q3 | Q3 | Q1 | Q1 | Q2 |  | Q1 |  |  |  | Q1 |  |  | Q1 |
|  | Senegal | Q2 | Q2 | Q2 | Q1 | Q2 | Q2 |  |  |  |  |  | Q1 |  |  | Q1 |
|  | Eritrea | Q2 | Q1 | Q1 | Q1 | Q1 | Q1 |  |  |  |  |  |  |  |  |  |
|  | Djibouti | Q3 | Q3 | Q4 | Q3 | Q3 | Q2 | Q1 |  | Q1 |  | Q1 | Q2 |  | Q1 | Q1 |
|  | Madagascar | Q2 | Q2 | Q1 | Q2 | Q2 | Q2 |  | Q1 | Q1 |  |  |  |  |  | Q1 |
|  | Kenya | Q2 | Q1 | Q1 | Q2 | Q1 | Q1 |  |  | Q1 |  |  |  |  |  |  |
|  | Sierra Leone | Q4 | Q4 | Q4 | Q1 | Q1 | Q1 |  |  |  |  |  | Q1 |  |  | Q1 |
| Latin America and the Caribbean | Virgin Islands (U.S.) |  |  |  |  |  |  |  |  |  |  |  |  |  |  |  |
|  | Brazil | Q4 | Q4 | Q4 | Q1 | Q1 | Q2 |  | Q2 | Q2 | Q4 |  | Q4 | Q2 |  | Q3 |
|  | Puerto Rico |  |  |  |  |  |  |  |  |  |  |  |  |  |  |  |
|  | Jamaica | Q2 | Q1 | Q2 | Q2 | Q2 | Q2 |  | Q1 | Q2 |  |  |  |  |  |  |
|  | Trinidad and Tobago | Q1 | Q2 | Q2 | Q2 | Q2 | Q2 | Q1 | Q2 | Q2 |  |  | Q3 |  |  | Q2 |
|  | Haiti | Q3 | Q1 | Q3 | Q1 | Q1 | Q1 | Q1 |  |  |  |  |  |  |  |  |
|  | Dominica | Q2 | Q2 | Q2 | Q3 | Q3 | Q3 | Q2 | Q3 | Q3 |  |  |  |  |  |  |
|  | Dominican Republic | Q3 | Q2 | Q2 | Q1 | Q2 | Q2 |  | Q2 | Q2 | Q2 |  |  | Q2 |  |  |
|  | St. Kitts and Nevis | Q1 | Q1 | Q2 | Q3 | Q2 | Q1 |  | Q4 | Q4 | Q4 |  |  | Q2 |  |  |
|  | St. Vincent and the Grenadines | Q1 | Q1 | Q1 | Q4 | Q4 | Q4 |  | Q3 | Q2 | Q3 |  |  | Q1 |  |  |
|  | Peru | Q2 | Q1 | Q2 | Q3 | Q3 | Q2 |  |  | Q2 |  |  | Q2 |  |  | Q2 |
|  | Mexico | Q2 | Q2 | Q2 | Q2 | Q2 | Q2 | Q1 |  | Q2 | Q1 |  |  | Q2 |  | Q3 |
|  | Guatemala | Q3 | Q3 | Q3 | Q1 | Q1 | Q1 |  | Q1 | Q1 |  |  |  |  |  |  |
|  | Bolivia | Q3 | Q2 | Q2 | Q3 | Q3 | Q3 |  |  |  |  |  |  |  |  |  |
|  | Paraguay | Q4 | Q2 | Q4 | Q1 | Q1 | Q1 |  | Q1 | Q1 |  |  |  | Q2 |  |  |
|  | St. Lucia | Q2 | Q3 | Q3 | Q2 | Q2 | Q2 | Q1 | Q2 |  |  |  |  |  |  |  |
|  | Ecuador | Q1 | Q3 | Q3 | Q1 | Q1 | Q1 |  |  | Q2 | Q2 |  | Q2 | Q2 |  | Q3 |
|  | Venezuela, RB | Q3 | Q2 | Q2 | Q2 | Q2 | Q1 |  |  |  |  |  |  |  |  |  |
|  | Uruguay | Q4 | Q4 | Q4 | Q2 | Q2 | Q3 |  | Q2 | Q1 |  |  | Q3 |  |  | Q4 |
|  | Suriname | Q4 | Q3 | Q2 | Q2 | Q2 | Q2 | Q2 |  | Q3 | Q2 |  |  | Q1 |  |  |
|  | El Salvador | Q4 | Q3 | Q3 | Q2 | Q2 | Q3 |  | Q1 | Q1 |  |  | Q1 | Q2 |  | Q2 |
|  | Guyana | Q3 | Q2 | Q2 | Q4 | Q4 | Q3 |  | Q2 |  | Q3 |  | Q1 | Q1 |  | Q1 |
|  | Nicaragua | Q2 | Q3 | Q3 | Q2 | Q2 | Q2 |  | Q1 | Q1 |  |  |  |  |  |  |
|  | Costa Rica | Q3 | Q3 | Q4 | Q4 | Q3 | Q3 |  |  | Q1 | Q1 |  |  | Q2 |  |  |
|  | Bahamas, The | Q2 | Q2 | Q3 | Q2 | Q2 | Q2 | Q2 |  | Q3 |  |  |  |  |  |  |
|  | Cuba | Q3 | Q4 | Q4 | Q4 | Q4 | Q4 |  | Q3 | Q4 |  |  | Q4 | Q4 |  | Q4 |
|  | Colombia | Q3 | Q2 | Q3 | Q4 | Q4 | Q3 |  |  |  |  |  | Q1 | Q2 | Q2 | Q2 |
|  | Barbados | Q3 | Q3 | Q3 | Q3 | Q3 | Q3 |  | Q4 | Q4 |  | Q3 | Q3 |  | Q3 | Q3 |
|  | Panama | Q4 | Q3 | Q3 | Q3 | Q3 | Q3 |  | Q2 | Q2 | Q3 |  |  | Q2 |  |  |
|  | Argentina | Q4 | Q4 | Q3 | Q2 | Q2 | Q3 | Q2 | Q3 | Q3 |  |  |  |  | Q4 | Q4 |
|  | Cayman Islands |  |  |  |  |  |  |  |  |  |  |  |  |  |  |  |
|  | Antigua and Barbuda | Q1 | Q2 | Q2 | Q3 | Q3 | Q4 |  | Q2 | Q2 |  |  |  |  |  |  |
|  | Aruba |  |  |  |  |  |  |  |  |  |  |  |  |  |  |  |
|  | Belize | Q1 | Q1 | Q2 | Q2 | Q3 | Q3 | Q1 | Q1 | Q1 | Q2 |  | Q2 | Q2 |  | Q2 |
|  | Grenada | Q3 | Q3 | Q2 | Q2 | Q2 | Q1 | Q3 | Q3 |  |  |  |  |  |  |  |
|  | Chile | Q4 | Q3 | Q3 | Q2 | Q1 | Q2 |  | Q2 | Q2 |  |  | Q1 |  |  | Q2 |
|  | Honduras | Q3 | Q3 | Q4 | Q2 | Q2 | Q2 |  |  | Q1 | Q2 | Q2 |  | Q1 | Q1 |  |
| North America | United States | Q4 | Q4 | Q4 | Q2 | Q2 | Q2 | Q2 | Q3 | Q3 | Q4 | Q4 | Q4 | Q3 |  | Q3 |
|  | Canada | Q4 | Q4 | Q4 | Q3 | Q3 | Q3 | Q2 | Q3 | Q3 |  |  |  | Q2 |  | Q3 |
| Asia | Yemen, Rep. | Q1 | Q2 | Q2 | Q2 | Q1 | Q1 |  | Q1 | Q1 |  |  | Q1 |  |  | Q1 |
|  | Israel | Q4 | Q3 | Q3 | Q3 | Q3 | Q3 | Q3 | Q4 | Q3 |  |  | Q3 | Q4 |  | Q4 |
|  | India | Q1 | Q1 | Q1 | Q1 | Q1 | Q1 |  | Q1 |  |  | Q2 | Q2 |  | Q2 | Q2 |
|  | Cambodia | Q3 | Q3 | Q2 | Q1 | Q1 | Q1 |  |  | Q1 | Q1 |  | Q2 | Q1 |  | Q1 |
|  | Vietnam | Q2 | Q2 | Q3 | Q1 | Q1 | Q2 |  | Q2 | Q2 |  |  | Q2 |  |  | Q2 |
|  | Indonesia | Q1 | Q1 | Q1 | Q1 | Q1 | Q1 |  |  | Q1 |  |  | Q2 | Q1 |  | Q1 |
|  | Pakistan | Q1 | Q1 | Q1 | Q1 | Q1 | Q1 |  | Q1 | Q1 |  | Q1 | Q1 |  | Q2 | Q2 |
|  | Iraq | Q1 | Q1 | Q1 | Q1 | Q3 | Q3 |  | Q1 | Q1 |  | Q2 | Q2 |  | Q2 | Q2 |
|  | Libya | Q1 | Q1 | Q1 | Q2 | Q3 | Q3 |  |  |  |  |  | Q4 |  |  | Q3 |
|  | Nepal | Q2 | Q2 | Q2 | Q1 | Q1 | Q1 |  |  |  |  |  |  |  |  |  |
|  | Syrian Arab Republic | Q2 | Q1 | Q1 | Q1 | Q2 | Q2 |  |  | Q2 |  |  | Q2 |  |  | Q2 |
|  | Uzbekistan | Q2 | Q2 | Q2 | Q2 | Q2 | Q2 | Q3 | Q3 | Q3 |  | Q4 | Q4 | Q3 | Q4 | Q3 |
|  | Qatar | Q1 | Q1 | Q1 | Q3 | Q4 | Q4 |  | Q2 |  |  | Q4 | Q4 |  | Q3 | Q4 |
|  | Philippines | Q1 | Q1 | Q1 | Q2 | Q1 | Q1 |  |  |  |  |  |  | Q1 |  |  |
|  | Kuwait | Q1 | Q1 | Q1 | Q4 | Q4 | Q4 |  | Q2 |  |  | Q3 | Q3 |  | Q3 | Q3 |
|  | Oman | Q1 | Q1 | Q1 | Q4 | Q4 | Q4 |  | Q2 |  |  | Q3 | Q3 |  | Q3 | Q3 |
|  | Lebanon | Q4 | Q3 | Q3 | Q1 | Q1 | Q1 |  | Q3 |  |  | Q2 | Q2 |  | Q3 | Q4 |
|  | Jordan | Q4 | Q4 | Q3 | Q2 | Q2 | Q3 |  | Q1 | Q2 |  | Q3 | Q3 |  | Q3 | Q3 |
|  | Armenia | Q3 | Q2 | Q1 | Q1 | Q1 | Q1 | Q3 | Q3 |  |  |  | Q3 | Q3 |  | Q4 |
|  | Brunei Darussalam | Q1 | Q1 | Q1 | Q4 | Q4 | Q4 | Q1 | Q2 |  |  |  | Q4 | Q2 |  | Q2 |
|  | Singapore | Q1 | Q1 | Q1 | Q2 | Q1 | Q1 |  | Q3 |  |  |  | Q4 |  |  | Q3 |
|  | Bahrain | Q1 | Q1 | Q1 | Q3 | Q3 | Q3 |  | Q2 |  |  | Q4 | Q3 |  | Q4 | Q2 |
|  | Saudi Arabia | Q1 | Q1 | Q1 | Q3 | Q3 | Q3 |  | Q2 |  |  |  | Q2 |  |  | Q2 |
|  | Tajikistan | Q2 | Q2 | Q2 | Q1 | Q1 | Q1 | Q3 | Q4 |  |  |  | Q3 | Q3 |  | Q3 |
|  | United Arab Emirates | Q1 | Q1 | Q1 | Q4 | Q2 | Q3 |  | Q2 |  |  |  | Q3 |  | Q2 | Q3 |
|  | Bangladesh | Q1 | Q1 | Q1 | Q1 | Q1 | Q1 |  | Q1 |  |  | Q1 | Q1 |  | Q1 | Q2 |
|  | Thailand | Q1 | Q1 | Q1 | Q3 | Q3 | Q4 | Q1 |  | Q2 | Q3 |  | Q2 | Q1 | Q1 | Q2 |
|  | Azerbaijan | Q2 | Q3 | Q2 | Q1 | Q1 | Q1 | Q4 | Q4 |  |  |  | Q4 | Q4 |  | Q4 |
|  | Hong Kong SAR, China |  |  |  |  |  |  |  |  |  |  |  |  |  |  |  |
|  | Malaysia | Q1 | Q1 | Q1 | Q3 | Q2 | Q2 |  | Q2 | Q2 |  |  | Q3 | Q1 |  | Q2 |
|  | China | Q2 | Q2 | Q2 | Q1 | Q1 | Q2 | Q1 | Q2 | Q3 | Q1 | Q1 | Q2 | Q2 | Q2 | Q3 |
|  | Turkey | Q2 | Q2 | Q3 | Q3 | Q3 | Q4 | Q1 | Q2 | Q2 |  |  | Q1 | Q2 | Q2 | Q2 |
|  | Macau |  |  |  |  |  |  |  |  |  |  |  |  |  |  |  |
|  | Korea, Rep. | Q1 | Q2 | Q3 | Q2 | Q2 | Q2 | Q3 |  |  |  |  | Q3 | Q2 |  | Q3 |
|  | Burma |  |  |  |  |  |  |  |  |  |  |  |  |  |  |  |
|  | Korea, Dem. Rep. |  |  |  |  |  |  |  |  |  |  |  |  |  |  |  |
|  | Timor-Leste | Q2 | Q3 | Q2 | Q3 | Q4 | Q4 |  |  | Q4 |  |  |  |  |  |  |
|  | Mongolia | Q2 | Q2 | Q2 | Q4 | Q2 | Q3 |  |  | Q4 |  |  | Q3 |  |  | Q3 |
|  | Russian Federation | Q2 | Q2 | Q2 | Q3 | Q3 | Q2 | Q4 | Q4 |  |  |  | Q4 | Q4 | Q4 | Q4 |
|  | West Bank and Gaza |  |  |  |  |  |  |  |  |  |  |  |  |  |  |  |
|  | Japan | Q4 | Q4 | Q4 | Q4 | Q4 | Q4 | Q4 | Q4 |  |  |  | Q3 | Q2 |  | Q3 |
|  | Turkmenistan | Q1 | Q1 | Q1 | Q4 | Q3 | Q2 |  |  |  |  |  | Q3 |  |  | Q3 |
|  | Maldives | Q3 | Q3 | Q2 | Q3 | Q2 | Q3 | Q1 | Q2 |  |  |  | Q3 | Q1 |  | Q2 |
|  | Afghanistan |  | Q4 | Q3 |  | Q1 | Q1 |  |  | Q1 |  | Q1 | Q1 |  | Q1 | Q1 |
|  | Lao PDR | Q1 | Q1 | Q1 | Q1 | Q1 | Q2 |  | Q1 | Q1 |  | Q1 | Q2 |  | Q1 | Q1 |
|  | Sri Lanka | Q1 | Q1 | Q1 | Q2 | Q2 | Q1 | Q1 |  |  |  |  | Q2 | Q1 |  | Q2 |
|  | Iran, Islamic Rep. | Q2 | Q2 | Q3 | Q2 | Q2 | Q1 |  | Q2 |  |  | Q2 | Q2 |  | Q2 | Q2 |
|  | Kyrgyz Republic | Q2 | Q2 | Q2 | Q2 | Q1 | Q2 | Q3 | Q3 |  |  |  | Q3 | Q3 |  | Q3 |
|  | Kazakhstan | Q1 | Q1 | Q1 | Q2 | Q3 | Q2 | Q3 | Q4 |  |  |  | Q4 | Q4 | Q4 | Q4 |
|  | Bhutan | Q3 | Q1 | Q1 | Q4 | Q4 | Q4 |  |  |  |  |  | Q1 |  |  | Q1 |
|  | Myanmar | Q1 | Q1 | Q1 | Q1 | Q1 | Q1 | Q1 |  |  |  |  | Q2 | Q1 |  | Q2 |
| Europe | Ireland | Q3 | Q3 | Q4 | Q4 | Q4 | Q3 | Q2 | Q4 | Q3 |  |  | Q4 | Q3 |  | Q4 |
|  | Belgium | Q4 | Q4 | Q4 | Q4 | Q4 | Q4 | Q3 | Q4 | Q4 |  |  | Q4 | Q4 |  | Q4 |
|  | Sweden | Q4 | Q4 | Q4 | Q4 | Q4 | Q4 | Q2 |  | Q3 |  |  | Q4 | Q3 |  | Q4 |
|  | Croatia | Q4 | Q3 | Q3 | Q4 | Q4 | Q4 | Q3 | Q4 | Q4 |  |  | Q3 | Q3 | Q3 | Q3 |
|  | Switzerland | Q4 | Q4 | Q4 | Q3 | Q3 | Q3 |  | Q4 | Q4 | Q4 |  | Q4 | Q4 |  | Q4 |
|  | Montenegro | Q4 | Q4 | Q3 | Q3 | Q3 | Q2 |  | Q3 | Q3 |  |  | Q3 |  |  | Q3 |
|  | Finland | Q4 | Q4 | Q4 | Q3 | Q4 | Q4 | Q4 | Q4 | Q4 |  |  | Q4 | Q3 |  | Q3 |
|  | Norway | Q4 | Q4 | Q4 | Q4 | Q4 | Q4 | Q2 | Q3 | Q3 |  |  | Q4 | Q3 |  | Q4 |
|  | Georgia | Q3 | Q4 | Q4 | Q1 | Q1 | Q1 | Q2 | Q3 |  |  |  | Q2 | Q4 |  | Q4 |
|  | Ukraine | Q3 | Q3 | Q3 | Q2 | Q3 | Q2 | Q4 | Q4 |  |  |  | Q4 | Q3 |  | Q4 |
|  | Germany | Q4 | Q4 | Q4 | Q4 | Q4 | Q4 | Q4 | Q4 | Q4 |  | Q4 | Q4 | Q4 |  | Q4 |
|  | Bosnia and Herzegovina | Q3 | Q4 | Q4 | Q3 | Q2 | Q3 | Q1 | Q3 | Q3 |  | Q3 | Q3 | Q2 | Q2 | Q3 |
|  | Latvia | Q3 | Q3 | Q2 | Q2 | Q2 | Q2 | Q4 | Q4 | Q4 |  |  | Q3 | Q4 |  | Q3 |
|  | Malta | Q3 | Q4 | Q3 | Q3 | Q3 | Q3 | Q3 | Q4 | Q3 |  |  | Q4 | Q3 |  | Q4 |
|  | Slovenia | Q4 | Q4 | Q4 | Q4 | Q3 | Q3 | Q3 | Q3 | Q3 |  | Q4 | Q4 | Q3 | Q3 | Q3 |
|  | Austria | Q4 | Q4 | Q4 | Q4 | Q4 | Q4 | Q4 | Q4 | Q4 |  |  | Q4 | Q3 |  | Q4 |
|  | Portugal | Q4 | Q4 | Q4 | Q3 | Q3 | Q3 | Q2 | Q3 | Q3 |  | Q3 | Q3 | Q3 | Q4 | Q4 |
|  | Luxembourg | Q4 | Q3 | Q3 | Q4 | Q4 | Q4 | Q3 |  | Q4 |  | Q4 | Q1 | Q3 | Q4 | Q3 |
|  | United Kingdom | Q3 | Q4 | Q4 | Q4 | Q4 | Q4 | Q2 |  | Q3 |  |  | Q4 | Q2 |  | Q3 |
|  | Czech Republic | Q3 | Q3 | Q3 | Q4 | Q4 | Q4 | Q4 | Q4 | Q4 |  |  | Q4 | Q4 |  | Q4 |
|  | Netherlands | Q4 | Q4 | Q4 | Q3 | Q3 | Q4 | Q3 |  |  |  |  |  | Q3 | Q4 | Q3 |
|  | San Marino | Q2 | Q2 | Q2 | Q4 | Q4 | Q4 |  |  |  |  |  |  |  |  |  |
|  | Belarus | Q3 | Q3 | Q2 | Q4 | Q4 | Q4 | Q4 | Q4 |  |  |  | Q4 | Q4 | Q4 | Q4 |
|  | Bulgaria | Q3 | Q3 | Q3 | Q3 | Q3 | Q2 | Q3 | Q4 | Q4 |  |  | Q3 | Q4 |  | Q4 |
|  | Serbia | Q4 | Q4 | Q4 | Q3 | Q3 | Q3 |  | Q4 |  |  |  | Q3 |  |  | Q3 |
|  | Andorra | Q3 | Q2 | Q3 | Q3 | Q3 | Q3 | Q1 | Q2 |  |  |  | Q3 | Q3 |  | Q4 |
|  | Estonia | Q2 | Q2 | Q2 | Q4 | Q4 | Q4 | Q3 |  | Q4 |  |  | Q4 | Q4 |  | Q4 |
|  | Hungary | Q4 | Q4 | Q3 | Q3 | Q3 | Q3 | Q4 | Q4 | Q4 |  |  | Q4 |  |  | Q4 |
|  | Iceland | Q4 | Q4 | Q4 | Q4 | Q4 | Q4 |  |  |  |  |  | Q4 | Q4 |  | Q4 |
|  | Italy | Q4 | Q4 | Q4 | Q4 | Q4 | Q4 | Q2 | Q3 | Q3 |  |  | Q1 | Q4 |  | Q4 |
|  | Poland | Q2 | Q3 | Q3 | Q3 | Q3 | Q3 | Q3 | Q3 | Q4 |  | Q3 | Q3 | Q3 | Q3 | Q3 |
|  | Romania | Q2 | Q2 | Q2 | Q4 | Q4 | Q4 | Q4 | Q4 | Q4 |  |  | Q3 | Q2 |  | Q3 |
|  | France | Q4 | Q4 | Q4 | Q4 | Q4 | Q4 | Q4 | Q4 | Q4 |  |  | Q1 | Q4 |  | Q4 |
|  | Slovak Republic | Q2 | Q3 | Q4 | Q4 | Q4 | Q3 | Q4 | Q4 | Q4 |  | Q4 | Q1 | Q3 |  | Q4 |
|  | Moldova | Q3 | Q4 | Q4 | Q2 | Q2 | Q2 | Q4 | Q4 |  |  |  | Q4 | Q3 |  | Q3 |
|  | Denmark | Q4 | Q4 | Q4 | Q4 | Q4 | Q4 | Q2 |  | Q3 |  |  | Q4 | Q3 |  | Q4 |
|  | Albania | Q3 | Q3 | Q2 | Q1 | Q2 | Q1 | Q1 | Q2 |  |  |  | Q3 | Q2 |  | Q2 |
|  | Lithuania | Q3 | Q2 | Q3 | Q3 | Q3 | Q3 | Q4 | Q4 | Q4 |  |  | Q4 | Q4 |  | Q4 |
|  | Cyprus | Q3 | Q3 | Q3 | Q2 | Q1 | Q1 |  | Q3 | Q3 |  |  | Q3 | Q3 |  | Q3 |
|  | Monaco | Q1 | Q1 | Q1 | Q4 | Q4 | Q4 |  |  |  |  |  |  |  |  |  |
|  | Spain | Q4 | Q4 | Q4 | Q3 | Q3 | Q3 | Q2 | Q3 | Q3 |  |  | Q3 | Q3 |  | Q4 |
|  | Macedonia, Q1YR | Q4 | Q3 | Q3 | Q3 | Q3 | Q3 | Q3 | Q3 | Q3 |  |  | Q1 | Q3 |  | Q3 |
|  | Greece | Q4 | Q4 | Q4 | Q3 | Q3 | Q3 | Q2 | Q3 |  |  | Q3 | Q1 | Q4 | Q4 | Q4 |
| Oceania | Fiji | Q1 | Q1 | Q1 | Q4 | Q4 | Q3 |  | Q2 |  |  |  | Q2 |  |  | Q2 |
|  | Micronesia, Q1ed. Sts. | Q4 | Q4 | Q4 | Q4 | Q4 | Q4 | Q1 | Q3 |  |  | Q2 | Q3 | Q1 | Q2 | Q1 |
|  | Samoa | Q3 | Q2 | Q2 | Q4 | Q4 | Q4 | Q1 | Q1 |  |  | Q1 | Q2 |  | Q1 | Q2 |
|  | Tonga | Q2 | Q3 | Q2 | Q3 | Q4 | Q4 |  |  | Q2 |  |  | Q3 | Q1 |  | Q2 |
|  | Guam |  |  |  |  |  |  |  |  |  |  |  |  |  |  |  |
|  | Vanuatu | Q1 | Q1 | Q1 | Q4 | Q3 | Q4 |  | Q3 |  |  |  | Q2 |  |  | Q1 |
|  | Solomon Islands | Q2 | Q3 | Q3 | Q4 | Q4 | Q4 |  | Q1 |  |  | Q2 | Q2 |  | Q1 | Q1 |
|  | Kiribati | Q4 | Q4 | Q4 | Q4 | Q4 | Q4 |  | Q1 | Q1 |  |  | Q3 |  |  | Q2 |
|  | Palau | Q4 | Q4 | Q4 | Q3 | Q4 | Q4 |  |  | Q4 |  |  | Q4 | Q2 |  | Q2 |
|  | New Caledonia |  |  |  |  |  |  |  |  |  |  |  |  |  |  |  |
|  | American Samoa |  |  |  |  |  |  |  |  |  |  |  |  |  |  |  |
|  | French Polynesia |  |  |  |  |  |  |  |  |  |  |  |  | Q2 |  |  |
|  | Marshall Islands | Q4 | Q4 | Q4 | Q4 | Q4 | Q4 |  |  | Q3 | Q3 |  | Q2 | Q1 |  | Q2 |
|  | New Zealand | Q4 | Q4 | Q4 | Q4 | Q4 | Q4 |  |  |  |  |  | Q4 | Q3 |  | Q3 |
|  | Australia | Q4 | Q4 | Q4 | Q3 | Q3 | Q3 | Q4 | Q3 | Q3 |  |  | Q4 | Q3 |  | Q4 |
|  | Northern Mariana Islands |  |  |  |  |  |  |  |  |  |  |  |  |  |  |  |
|  | Papua New Guinea | Q1 | Q1 | Q1 | Q4 | Q4 | Q4 |  |  |  | Q1 |  | Q1 | Q1 |  | Q1 |
|  | Tuvalu | Q4 | Q4 | Q4 | Q4 | Q4 | Q4 |  |  |  |  |  | Q4 |  |  | Q2 |
|  | Nauru |  |  |  |  |  |  |  |  |  |  |  |  |  |  |  |

| **Region** | **Country** | **DPT vaccination coverage** | | | **Ethnic fragmentation** | **Linguistic fragmentation** | **Religious fragmentation** | **Index of corruption** | | | |
| --- | --- | --- | --- | --- | --- | --- | --- | --- | --- | --- | --- |
|  |  | **2000** | **2005** | **2010** | **2000** | **2000** | **2000** | **2007** | **2008** | **2009** | **2010** |
| Africa | Sudan | Q1 | Q1 | Q2 | Q4 | Q4 | Q2 | Q1 | Q1 | Q1 | Q1 |
|  | Comoros | Q1 | Q1 | Q1 | Q1 | Q1 | Q1 | Q2 | Q1 | Q1 | Q1 |
|  | Tunisia | Q4 | Q4 | Q4 | Q1 | Q1 | Q1 | Q3 | Q3 | Q3 | Q3 |
|  | Gambia, The | Q2 | Q3 | Q3 | Q4 | Q4 | Q1 | Q1 | Q1 | Q2 | Q2 |
|  | Guinea-Bissau | Q1 | Q1 | Q1 | Q4 | Q4 | Q3 | Q1 | Q1 | Q1 | Q1 |
|  | Mauritania | Q1 | Q1 | Q1 | Q3 | Q2 | Q1 | Q2 | Q2 | Q1 | Q1 |
|  | Egypt, Arab Rep. | Q4 | Q4 | Q3 | Q1 | Q1 | Q1 | Q2 | Q2 | Q2 | Q2 |
|  | Algeria | Q2 | Q2 | Q3 | Q2 | Q3 | Q1 | Q2 | Q2 | Q2 | Q2 |
|  | Rwanda | Q2 | Q3 | Q3 | Q2 |  | Q3 | Q2 | Q2 | Q2 | Q3 |
|  | Morocco | Q3 | Q4 | Q4 | Q3 | Q3 | Q1 | Q3 | Q3 | Q2 | Q3 |
|  | Central African Republic | Q1 | Q1 | Q1 | Q4 | Q4 | Q4 | Q1 | Q1 | Q1 | Q1 |
|  | Burundi | Q2 | Q2 | Q3 | Q2 | Q2 | Q3 | Q1 | Q1 | Q1 | Q1 |
|  | Nigeria | Q1 | Q1 | Q1 | Q4 | Q4 | Q4 | Q1 | Q2 | Q1 | Q1 |
|  | Botswana | Q4 | Q3 | Q3 | Q2 | Q3 | Q3 | Q4 | Q4 | Q4 | Q4 |
|  | Cote d'Ivoire | Q1 | Q1 | Q2 | Q4 | Q4 | Q4 | Q1 | Q1 | Q1 | Q1 |
|  | Angola | Q1 | Q1 | Q2 | Q4 | Q4 | Q3 | Q1 | Q1 | Q1 | Q1 |
|  | South Africa | Q1 | Q1 | Q1 | Q4 | Q4 | Q4 | Q4 | Q3 | Q3 | Q3 |
|  | Mali | Q1 | Q1 | Q1 | Q4 | Q4 | Q1 | Q2 | Q2 | Q2 | Q2 |
|  | Benin | Q2 | Q1 | Q1 | Q4 | Q4 | Q3 | Q2 | Q2 | Q2 | Q2 |
|  | Congo, Rep. | Q1 | Q1 | Q2 | Q4 | Q4 | Q4 | Q1 | Q1 | Q1 | Q1 |
|  | Somalia | Q1 | Q1 | Q1 | Q4 | Q1 | Q1 | Q1 | Q1 | Q1 | Q1 |
|  | Congo, Dem. Rep. | Q1 | Q1 | Q1 | Q4 | Q4 | Q4 | Q1 | Q1 | Q1 | Q1 |
|  | Liberia | Q1 | Q1 | Q1 | Q4 | Q4 | Q3 | Q1 | Q1 | Q2 | Q2 |
|  | Ghana | Q2 | Q2 | Q2 | Q4 | Q4 | Q4 | Q3 | Q3 | Q3 | Q3 |
|  | Cabo Verde | Q2 | Q3 | Q4 | Q2 |  | Q1 | Q3 | Q3 | Q3 | Q3 |
|  | Zambia | Q2 | Q2 | Q1 | Q4 | Q4 | Q4 | Q2 | Q2 | Q2 | Q2 |
|  | Mozambique | Q1 | Q1 | Q1 | Q4 | Q4 | Q4 | Q2 | Q2 | Q1 | Q2 |
|  | Niger | Q1 | Q1 | Q1 | Q3 | Q4 | Q1 | Q2 | Q2 | Q2 | Q2 |
|  | Cameroon | Q1 | Q1 | Q1 | Q4 | Q4 | Q4 | Q1 | Q1 | Q1 | Q1 |
|  | Sao Tome and Principe | Q2 | Q3 | Q4 |  | Q2 | Q1 | Q2 | Q2 | Q2 | Q2 |
|  | Uganda | Q1 | Q1 | Q1 | Q4 | Q4 | Q3 | Q2 | Q2 | Q1 | Q2 |
|  | Ethiopia | Q1 | Q1 | Q1 | Q4 | Q4 | Q3 | Q1 | Q2 | Q2 | Q2 |
|  | Gabon | Q1 | Q1 | Q1 | Q4 | Q4 | Q4 | Q2 | Q2 | Q2 | Q2 |
|  | Chad | Q1 | Q1 | Q1 | Q4 | Q4 | Q4 | Q1 | Q1 | Q1 | Q1 |
|  | Zimbabwe | Q2 | Q1 | Q2 | Q2 | Q3 | Q4 | Q1 | Q1 | Q1 | Q1 |
|  | Seychelles | Q4 | Q4 | Q4 | Q2 | Q2 | Q1 | Q3 | Q3 | Q3 | Q3 |
|  | Namibia | Q2 | Q2 | Q1 | Q3 | Q4 | Q4 | Q3 | Q3 | Q3 | Q3 |
|  | Guinea | Q1 | Q1 | Q1 | Q4 | Q4 | Q2 | Q1 | Q1 | Q1 | Q1 |
|  | Malawi | Q1 | Q2 | Q2 | Q4 | Q3 | Q4 | Q2 | Q2 | Q2 | Q3 |
|  | Mauritius | Q2 | Q3 | Q4 | Q3 | Q3 | Q4 | Q3 | Q4 | Q4 | Q4 |
|  | Tanzania | Q2 | Q2 | Q2 | Q4 | Q4 | Q3 | Q2 | Q2 | Q2 | Q2 |
|  | Burkina Faso | Q1 | Q2 | Q2 | Q4 | Q4 | Q3 | Q2 | Q3 | Q3 | Q2 |
|  | Swaziland | Q2 | Q2 | Q2 | Q1 | Q2 | Q2 | Q2 | Q3 | Q3 | Q2 |
|  | Lesotho | Q2 | Q2 | Q1 | Q2 | Q2 | Q4 | Q2 | Q2 | Q2 | Q3 |
|  | Equatorial Guinea | Q1 | Q1 | Q1 | Q2 | Q2 | Q1 | Q1 | Q1 | Q1 | Q1 |
|  | Togo | Q1 | Q2 | Q2 | Q4 | Q4 | Q4 | Q1 | Q2 | Q2 | Q1 |
|  | Senegal | Q1 | Q2 | Q2 | Q4 | Q4 | Q1 | Q3 | Q2 | Q2 | Q2 |
|  | Eritrea | Q2 | Q3 | Q4 | Q3 | Q4 | Q2 | Q2 | Q2 | Q2 | Q2 |
|  | Djibouti | Q1 | Q1 | Q2 | Q4 | Q4 | Q1 | Q2 | Q2 | Q2 | Q2 |
|  | Madagascar | Q1 | Q2 | Q2 | Q4 | Q1 | Q3 | Q2 | Q2 | Q2 | Q2 |
|  | Kenya | Q2 | Q1 | Q1 | Q4 | Q4 | Q4 | Q1 | Q1 | Q1 | Q1 |
|  | Sierra Leone | Q1 | Q1 | Q1 | Q4 | Q4 | Q3 | Q1 | Q1 | Q1 | Q1 |
| Latin America and the Caribbean | Virgin Islands (U.S.) |  |  |  |  | Q2 | Q3 |  |  |  |  |
|  | Brazil | Q4 | Q3 | Q4 | Q3 | Q1 | Q3 | Q3 | Q3 | Q3 | Q3 |
|  | Puerto Rico |  |  |  |  | Q1 | Q3 |  | Q4 | Q4 | Q4 |
|  | Jamaica | Q3 | Q4 | Q4 | Q2 | Q1 | Q3 | Q2 | Q2 | Q2 | Q2 |
|  | Trinidad and Tobago | Q2 | Q3 | Q2 | Q3 | Q1 | Q4 | Q3 | Q3 | Q3 | Q3 |
|  | Haiti | Q1 | Q1 | Q1 | Q1 |  | Q3 | Q1 | Q1 | Q1 | Q1 |
|  | Dominica | Q4 | Q4 | Q4 | Q2 |  | Q2 | Q4 | Q4 | Q4 | Q4 |
|  | Dominican Republic | Q2 | Q2 | Q2 | Q2 | Q1 | Q2 | Q2 | Q2 | Q2 | Q2 |
|  | St. Kitts and Nevis | Q4 | Q4 | Q3 | Q1 |  | Q4 |  |  |  |  |
|  | St. Vincent and the Grenadines | Q4 | Q4 | Q4 | Q2 | Q1 | Q4 | Q4 | Q4 | Q4 |  |
|  | Peru | Q4 | Q3 | Q2 | Q3 | Q2 | Q1 | Q3 | Q3 | Q3 | Q3 |
|  | Mexico | Q4 | Q4 | Q3 | Q3 | Q2 | Q1 | Q3 | Q3 | Q2 | Q2 |
|  | Guatemala | Q2 | Q2 | Q2 | Q3 | Q3 | Q2 | Q2 | Q2 | Q3 | Q2 |
|  | Bolivia | Q1 | Q2 | Q1 | Q4 | Q2 | Q1 | Q2 | Q2 | Q2 | Q2 |
|  | Paraguay | Q2 | Q3 | Q2 | Q1 | Q3 | Q1 | Q1 | Q1 | Q1 | Q1 |
|  | St. Lucia | Q1 | Q3 | Q3 | Q1 | Q2 | Q2 | Q4 | Q4 | Q4 |  |
|  | Ecuador | Q2 | Q3 | Q4 | Q3 | Q2 | Q1 | Q1 | Q1 | Q1 | Q2 |
|  | Venezuela, RB | Q2 | Q2 | Q1 | Q3 | Q1 | Q1 | Q1 | Q1 | Q1 | Q1 |
|  | Uruguay | Q2 | Q3 | Q3 | Q2 | Q1 | Q2 | Q4 | Q4 | Q4 | Q4 |
|  | Suriname | Q1 | Q2 | Q3 | Q4 | Q2 | Q4 | Q3 | Q3 | Q3 |  |
|  | El Salvador | Q4 | Q2 | Q2 | Q1 |  | Q2 | Q3 | Q3 | Q3 | Q3 |
|  | Guyana | Q2 | Q2 | Q3 | Q3 | Q1 | Q4 | Q2 | Q2 | Q2 | Q2 |
|  | Nicaragua | Q2 | Q2 | Q4 | Q3 | Q1 | Q2 | Q2 | Q1 | Q1 | Q2 |
|  | Costa Rica | Q2 | Q2 | Q2 | Q2 | Q1 | Q2 | Q3 | Q3 | Q4 | Q4 |
|  | Bahamas, The | Q4 | Q2 | Q4 | Q2 | Q2 | Q4 |  |  |  |  |
|  | Cuba | Q3 | Q2 | Q3 | Q3 |  | Q3 | Q3 | Q3 | Q3 | Q3 |
|  | Colombia | Q2 | Q2 | Q2 | Q3 | Q1 | Q1 | Q3 | Q3 | Q3 | Q3 |
|  | Barbados | Q3 | Q2 | Q2 | Q1 | Q1 | Q4 | Q4 | Q4 | Q4 | Q4 |
|  | Panama | Q4 | Q2 | Q2 | Q3 | Q3 | Q2 | Q2 | Q2 | Q3 | Q3 |
|  | Argentina | Q2 | Q4 | Q2 | Q2 | Q1 | Q1 | Q2 | Q2 | Q2 | Q2 |
|  | Cayman Islands |  |  |  |  |  |  |  |  |  |  |
|  | Antigua and Barbuda | Q3 | Q4 | Q4 | Q1 | Q1 | Q4 |  |  |  |  |
|  | Aruba |  |  |  |  | Q3 | Q2 |  |  |  |  |
|  | Belize | Q3 | Q3 | Q3 | Q4 | Q3 | Q3 | Q2 | Q2 |  |  |
|  | Grenada | Q4 | Q4 | Q3 | Q2 |  | Q3 | Q3 |  |  |  |
|  | Chile | Q3 | Q2 | Q2 | Q1 | Q2 | Q2 | Q4 | Q4 | Q4 | Q4 |
|  | Honduras | Q3 | Q4 | Q3 | Q1 | Q1 | Q2 | Q1 | Q2 | Q1 | Q1 |
| North America | United States | Q3 | Q3 | Q3 | Q3 | Q2 | Q4 | Q4 | Q4 | Q4 | Q4 |
|  | Canada | Q3 | Q3 | Q3 | Q4 | Q3 | Q4 | Q4 | Q4 | Q4 | Q4 |
| Asia | Yemen, Rep. | Q1 | Q2 | Q2 |  | Q1 | Q1 | Q1 | Q1 | Q1 | Q1 |
|  | Israel | Q3 | Q2 | Q2 | Q2 | Q3 | Q2 | Q4 | Q4 | Q4 | Q4 |
|  | India | Q1 | Q1 | Q1 | Q2 | Q4 | Q2 | Q3 | Q2 | Q3 | Q2 |
|  | Cambodia | Q1 | Q2 | Q2 | Q2 | Q2 | Q1 | Q1 | Q1 | Q1 | Q1 |
|  | Vietnam | Q3 | Q3 | Q2 | Q2 | Q2 | Q3 | Q2 | Q2 | Q2 | Q2 |
|  | Indonesia | Q1 | Q1 | Q1 | Q4 | Q4 | Q2 | Q1 | Q2 | Q2 | Q2 |
|  | Pakistan | Q1 | Q1 | Q2 | Q4 | Q4 | Q2 | Q1 | Q1 | Q1 | Q1 |
|  | Iraq | Q2 | Q1 | Q1 | Q2 | Q3 | Q3 | Q1 | Q1 | Q1 | Q1 |
|  | Libya | Q3 | Q4 | Q4 | Q4 | Q1 | Q1 | Q1 | Q2 | Q1 | Q1 |
|  | Nepal | Q1 | Q1 | Q1 | Q4 | Q4 | Q1 | Q1 | Q2 | Q1 | Q1 |
|  | Syrian Arab Republic | Q2 | Q1 | Q1 | Q3 | Q2 | Q2 | Q1 | Q1 | Q2 | Q2 |
|  | Uzbekistan | Q4 | Q4 | Q4 | Q2 | Q3 | Q1 | Q1 | Q1 | Q1 | Q1 |
|  | Qatar | Q2 | Q3 | Q3 | Q4 | Q3 | Q1 | Q4 | Q4 | Q4 | Q4 |
|  | Philippines | Q2 | Q2 | Q1 | Q2 | Q4 | Q2 | Q1 | Q1 | Q1 | Q1 |
|  | Kuwait | Q4 | Q4 | Q4 | Q3 | Q2 | Q4 | Q3 | Q3 | Q3 | Q3 |
|  | Oman | Q4 | Q4 | Q4 | Q3 | Q2 | Q2 | Q3 | Q4 | Q4 | Q4 |
|  | Lebanon | Q2 | Q1 | Q1 | Q1 | Q2 | Q4 | Q2 | Q2 | Q1 | Q2 |
|  | Jordan | Q3 | Q3 | Q4 | Q3 | Q1 | Q1 | Q3 | Q3 | Q3 | Q3 |
|  | Armenia | Q3 | Q2 | Q2 | Q1 | Q1 | Q2 | Q2 | Q2 | Q2 | Q2 |
|  | Brunei Darussalam | Q4 | Q3 | Q3 | Q3 | Q2 | Q2 |  |  | Q4 | Q4 |
|  | Singapore | Q4 | Q3 | Q3 | Q2 | Q3 | Q4 | Q4 | Q4 | Q4 | Q4 |
|  | Bahrain | Q4 | Q4 | Q4 | Q3 | Q3 | Q3 | Q3 | Q4 | Q3 | Q3 |
|  | Saudi Arabia | Q3 | Q3 | Q4 | Q1 | Q1 | Q1 | Q3 | Q3 | Q3 | Q3 |
|  | Tajikistan | Q2 | Q2 | Q2 | Q3 | Q3 | Q2 | Q1 | Q1 | Q1 | Q1 |
|  | United Arab Emirates | Q3 | Q3 | Q2 | Q3 | Q3 | Q2 | Q4 | Q4 | Q4 | Q4 |
|  | Bangladesh | Q2 | Q3 | Q3 | Q1 | Q1 | Q1 | Q1 | Q1 | Q1 | Q1 |
|  | Thailand | Q4 | Q4 | Q4 | Q3 | Q3 | Q1 | Q2 | Q3 | Q3 | Q3 |
|  | Azerbaijan | Q1 | Q1 | Q1 | Q2 | Q2 | Q3 | Q1 | Q1 | Q1 | Q1 |
|  | Hong Kong SAR, China |  |  |  | Q1 | Q2 | Q2 | Q4 | Q4 | Q4 | Q4 |
|  | Malaysia | Q3 | Q3 | Q2 | Q3 | Q3 | Q4 | Q4 | Q3 | Q3 | Q3 |
|  | China | Q2 | Q2 | Q4 | Q1 | Q2 | Q4 | Q3 | Q3 | Q3 | Q3 |
|  | Turkey | Q2 | Q2 | Q3 | Q2 | Q2 | Q1 | Q3 | Q3 | Q3 | Q3 |
|  | Macau |  |  |  |  | Q2 | Q3 | Q4 | Q4 |  |  |
|  | Korea, Rep. | Q4 | Q3 | Q2 | Q1 | Q1 | Q3 |  |  |  |  |
|  | Burma |  |  |  | Q3 | Q3 | Q1 |  |  |  |  |
|  | Korea, Dem. Rep. | Q1 | Q1 | Q2 | Q1 | Q1 | Q4 | Q4 | Q4 | Q4 | Q4 |
|  | Timor-Leste |  | Q1 | Q1 |  |  |  | Q2 | Q1 | Q1 | Q2 |
|  | Mongolia | Q3 | Q4 | Q3 | Q2 | Q3 | Q1 | Q2 | Q2 | Q2 | Q2 |
|  | Russian Federation | Q3 | Q4 | Q3 | Q2 | Q2 | Q2 | Q1 | Q1 | Q1 | Q1 |
|  | West Bank and Gaza |  |  |  |  | Q1 | Q1 |  |  |  |  |
|  | Japan | Q2 | Q4 | Q4 | Q1 | Q1 | Q3 | Q4 | Q4 | Q4 | Q4 |
|  | Turkmenistan |  |  |  | Q2 | Q3 | Q1 | Q1 | Q1 | Q1 | Q1 |
|  | Maldives | Q4 | Q4 | Q3 |  |  |  | Q2 | Q2 | Q1 | Q1 |
|  | Afghanistan | Q1 | Q1 | Q1 | Q4 | Q3 | Q2 | Q1 | Q1 | Q1 | Q1 |
|  | Lao PDR | Q1 | Q1 | Q1 | Q3 | Q4 | Q3 | Q1 | Q1 | Q1 | Q1 |
|  | Sri Lanka | Q4 | Q4 | Q4 | Q2 | Q3 | Q3 | Q2 | Q2 | Q2 | Q2 |
|  | Iran, Islamic Rep. | Q4 | Q3 | Q4 | Q4 | Q4 | Q1 | Q1 | Q1 | Q1 | Q1 |
|  | Kyrgyz Republic | Q4 | Q4 | Q3 | Q4 | Q3 | Q2 | Q1 | Q1 | Q1 | Q1 |
|  | Kazakhstan | Q4 | Q4 | Q4 | Q3 | Q4 | Q3 | Q1 | Q1 | Q2 | Q2 |
|  | Bhutan | Q3 | Q3 | Q2 | Q3 | Q3 | Q2 | Q3 | Q3 | Q3 | Q4 |
|  | Myanmar | Q2 | Q1 | Q2 |  |  |  | Q1 | Q1 | Q1 | Q1 |
| Europe | Ireland | Q2 | Q2 | Q2 | Q1 | Q1 | Q1 | Q4 | Q4 | Q4 | Q4 |
|  | Belgium | Q3 | Q3 | Q4 | Q3 | Q3 | Q1 | Q4 | Q4 | Q4 | Q4 |
|  | Sweden | Q4 | Q4 | Q4 | Q1 | Q2 | Q2 | Q4 | Q4 | Q4 | Q4 |
|  | Croatia | Q3 | Q3 | Q3 | Q2 | Q1 | Q2 | Q3 | Q3 | Q3 | Q3 |
|  | Switzerland | Q2 | Q3 | Q3 | Q3 | Q3 | Q3 | Q4 | Q4 | Q4 | Q4 |
|  | Montenegro |  |  | Q2 |  |  |  | Q2 | Q2 | Q3 | Q3 |
|  | Finland | Q4 | Q3 | Q4 | Q1 | Q2 | Q2 | Q4 | Q4 | Q4 | Q4 |
|  | Norway | Q2 | Q2 | Q2 | Q1 | Q1 | Q1 | Q4 | Q4 | Q4 | Q4 |
|  | Georgia | Q2 | Q2 | Q2 | Q3 | Q3 | Q4 | Q3 | Q3 | Q3 | Q3 |
|  | Ukraine | Q4 | Q3 | Q1 | Q3 | Q3 | Q3 | Q2 | Q1 | Q1 | Q1 |
|  | Germany | Q2 | Q2 | Q2 | Q1 | Q2 | Q4 | Q4 | Q4 | Q4 | Q4 |
|  | Bosnia and Herzegovina | Q2 | Q2 | Q2 | Q3 | Q4 | Q4 | Q2 | Q2 | Q2 | Q2 |
|  | Latvia | Q3 | Q4 | Q2 | Q3 | Q3 | Q3 | Q3 | Q3 | Q3 | Q3 |
|  | Malta | Q3 | Q2 | Q1 | Q1 | Q1 | Q1 | Q4 | Q4 | Q4 | Q4 |
|  | Slovenia | Q3 | Q3 | Q3 | Q2 | Q2 | Q2 | Q4 | Q4 | Q4 | Q4 |
|  | Austria | Q2 | Q2 | Q1 | Q1 | Q2 | Q2 | Q4 | Q4 | Q4 | Q4 |
|  | Portugal | Q3 | Q2 | Q4 | Q1 | Q1 | Q1 | Q4 | Q4 | Q4 | Q4 |
|  | Luxembourg | Q4 | Q4 | Q4 | Q3 | Q4 | Q1 | Q4 | Q4 | Q4 | Q4 |
|  | United Kingdom | Q3 | Q2 | Q2 | Q1 | Q1 | Q4 | Q4 | Q4 | Q4 | Q4 |
|  | Czech Republic | Q4 | Q3 | Q4 | Q2 | Q2 | Q4 | Q4 | Q3 | Q3 | Q3 |
|  | Netherlands | Q4 | Q4 | Q3 | Q1 | Q3 | Q4 | Q4 | Q4 | Q4 | Q4 |
|  | San Marino | Q3 | Q3 | Q1 | Q2 |  | Q1 |  |  |  |  |
|  | Belarus | Q4 | Q4 | Q4 | Q2 | Q3 | Q3 | Q1 | Q1 | Q1 | Q2 |
|  | Bulgaria | Q3 | Q3 | Q2 | Q2 | Q2 | Q3 | Q3 | Q3 | Q3 | Q3 |
|  | Serbia | Q3 | Q4 | Q2 | Q3 |  |  | Q3 | Q2 | Q3 | Q3 |
|  | Andorra |  |  |  | Q4 | Q4 | Q1 |  |  |  |  |
|  | Estonia | Q3 | Q3 | Q2 | Q3 | Q3 | Q3 | Q4 | Q4 | Q4 | Q4 |
|  | Hungary | Q4 | Q4 | Q4 | Q1 | Q1 | Q3 | Q4 | Q3 | Q3 | Q3 |
|  | Iceland | Q4 | Q3 | Q3 | Q1 | Q1 | Q1 | Q4 | Q4 | Q4 | Q4 |
|  | Italy | Q2 | Q3 | Q3 | Q1 | Q1 | Q2 | Q4 | Q3 | Q3 | Q3 |
|  | Poland | Q4 | Q4 | Q4 | Q1 | Q1 | Q1 | Q3 | Q3 | Q3 | Q4 |
|  | Romania | Q4 | Q3 | Q2 | Q2 | Q2 | Q2 | Q3 | Q3 | Q3 | Q3 |
|  | France | Q4 | Q4 | Q4 | Q1 | Q1 | Q2 | Q4 | Q4 | Q4 | Q4 |
|  | Slovak Republic | Q4 | Q4 | Q4 | Q2 | Q2 | Q3 | Q3 | Q3 | Q3 | Q3 |
|  | Moldova | Q3 | Q4 | Q2 | Q3 | Q3 | Q3 | Q2 | Q2 | Q2 | Q2 |
|  | Denmark | Q4 | Q2 | Q2 | Q1 | Q1 | Q2 | Q4 | Q4 | Q4 | Q4 |
|  | Albania | Q4 | Q4 | Q4 | Q2 | Q1 | Q3 | Q2 | Q2 | Q2 | Q2 |
|  | Lithuania | Q3 | Q3 | Q3 | Q2 | Q2 | Q2 | Q3 | Q3 | Q3 | Q3 |
|  | Cyprus | Q4 | Q4 | Q4 | Q1 | Q3 | Q2 | Q4 | Q4 | Q4 | Q4 |
|  | Monaco | Q4 | Q4 | Q4 | Q4 | Q4 | Q2 |  |  |  |  |
|  | Spain | Q3 | Q3 | Q3 | Q2 | Q3 | Q2 | Q4 | Q4 | Q4 | Q4 |
|  | Macedonia, Q1YR | Q3 | Q3 | Q3 | Q3 | Q3 | Q3 | Q2 | Q3 | Q3 | Q3 |
|  | Greece | Q2 | Q3 | Q4 | Q1 | Q1 | Q1 | Q3 | Q3 | Q3 | Q3 |
| Oceania | Fiji | Q2 | Q3 | Q4 | Q3 | Q3 | Q3 |  |  |  |  |
|  | Micronesia, Q1ed. Sts. | Q2 | Q3 | Q2 | Q4 | Q4 | Q4 |  |  |  |  |
|  | Samoa | Q4 | Q1 | Q2 | Q1 | Q1 | Q4 | Q3 | Q3 | Q3 | Q3 |
|  | Tonga | Q3 | Q4 | Q4 | Q1 | Q3 | Q3 | Q1 | Q1 | Q2 | Q2 |
|  | Guam |  |  |  |  | Q4 | Q2 |  |  |  |  |
|  | Vanuatu | Q1 | Q1 | Q1 | Q1 | Q3 | Q4 | Q2 | Q2 | Q2 | Q3 |
|  | Solomon Islands | Q2 | Q1 | Q1 | Q1 | Q3 | Q4 | Q2 | Q2 | Q2 | Q2 |
|  | Kiribati | Q2 | Q1 | Q2 | Q1 | Q1 | Q3 | Q2 | Q2 | Q2 | Q2 |
|  | Palau | Q3 | Q4 | Q1 | Q2 | Q2 | Q4 |  |  |  |  |
|  | New Caledonia |  |  |  |  | Q4 | Q3 |  |  |  |  |
|  | American Samoa |  |  |  |  | Q2 | Q4 |  |  |  |  |
|  | French Polynesia |  |  |  |  | Q3 | Q3 |  |  |  |  |
|  | Marshall Islands | Q1 | Q1 | Q2 | Q1 | Q1 | Q3 |  |  |  |  |
|  | New Zealand | Q2 | Q2 | Q2 | Q2 | Q2 | Q4 | Q4 | Q4 | Q4 | Q4 |
|  | Australia | Q2 | Q2 | Q2 | Q1 | Q2 | Q4 | Q4 | Q4 | Q4 | Q4 |
|  | Northern Mariana Islands |  |  |  |  | Q4 | Q3 |  |  |  |  |
|  | Papua New Guinea | Q1 | Q1 | Q1 | Q2 | Q2 | Q3 | Q1 | Q1 | Q1 | Q1 |
|  | Tuvalu |  |  |  | Q1 | Q2 | Q2 |  |  |  |  |
|  | Nauru |  |  |  | Q3 | Q3 | Q3 |  |  |  |  |

| **Region** | **Country** | **Gini Coefficient** | **Index of freedom** | | |
| --- | --- | --- | --- | --- | --- |
|  |  | **2005** | **2000** | **2005** | **2010** |
| Africa | Sudan | Q2 | 3 | 3 | 3 |
|  | Comoros | Q4 | 2 | 2 | 2 |
|  | Tunisia | Q2 | 3 | 3 | 3 |
|  | Gambia, The | Q4 | 3 | 2 | 2 |
|  | Guinea-Bissau | Q2 | 2 | 2 | 2 |
|  | Mauritania | Q3 | 3 | 2 | 3 |
|  | Egypt, Arab Rep. | Q1 | 3 | 3 | 3 |
|  | Algeria | Q2 | 3 | 3 | 3 |
|  | Rwanda | Q4 | 3 | 3 | 3 |
|  | Morocco | Q3 | 2 | 2 | 2 |
|  | Central African Republic | Q4 | 2 | 2 | 2 |
|  | Burundi | Q1 | 3 | 2 | 2 |
|  | Nigeria | Q4 | 2 | 2 | 2 |
|  | Botswana | Q4 | 1 | 1 | 1 |
|  | Cote d'Ivoire | Q3 | 2 | 3 | 3 |
|  | Angola | Q3 | 3 | 3 | 3 |
|  | South Africa | Q4 |  |  |  |
|  | Mali | Q1 | 1 | 1 | 1 |
|  | Benin | Q2 | 1 | 1 | 1 |
|  | Congo, Rep. | Q4 | 1 | 1 | 1 |
|  | Somalia |  | 2 | 2 | 3 |
|  | Congo, Dem. Rep. | Q3 | 3 | 3 | 3 |
|  | Liberia | Q2 | 3 | 3 | 3 |
|  | Ghana | Q3 | 2 | 2 | 2 |
|  | Cabo Verde | Q4 |  |  |  |
|  | Zambia | Q4 | 1 | 1 | 1 |
|  | Mozambique | Q4 | 1 | 1 | 1 |
|  | Niger | Q2 | 2 | 2 | 2 |
|  | Cameroon | Q3 | 2 | 2 | 2 |
|  | Sao Tome and Principe | Q4 | 2 | 2 | 2 |
|  | Uganda | Q3 | 3 | 3 | 3 |
|  | Ethiopia | Q1 | 1 | 1 | 1 |
|  | Gabon | Q3 | 2 | 2 | 2 |
|  | Chad | Q3 | 2 | 2 | 3 |
|  | Zimbabwe | Q4 | 2 | 2 | 3 |
|  | Seychelles | Q4 | 3 | 3 | 3 |
|  | Namibia | Q4 | 2 | 3 | 3 |
|  | Guinea | Q3 | 2 | 2 | 2 |
|  | Malawi | Q3 | 1 | 1 | 1 |
|  | Mauritius |  | 3 | 3 | 2 |
|  | Tanzania | Q2 | 2 | 2 | 2 |
|  | Burkina Faso | Q3 | 1 | 1 | 1 |
|  | Swaziland | Q4 | 2 | 2 | 2 |
|  | Lesotho | Q4 | 2 | 2 | 2 |
|  | Equatorial Guinea |  | 3 | 3 | 3 |
|  | Togo | Q2 | 2 | 1 | 2 |
|  | Senegal | Q3 | 3 | 3 | 3 |
|  | Eritrea |  | 2 | 3 | 2 |
|  | Djibouti | Q3 | 2 | 1 | 2 |
|  | Madagascar | Q3 | 3 | 3 | 3 |
|  | Kenya | Q4 | 2 | 2 | 3 |
|  | Sierra Leone | Q3 | 2 | 2 | 2 |
| Latin America and the Caribbean | Virgin Islands (U.S.) |  | 3 | 2 | 2 |
|  | Brazil | Q4 | 2 | 2 | 2 |
|  | Puerto Rico |  |  |  |  |
|  | Jamaica | Q3 | 2 | 1 | 1 |
|  | Trinidad and Tobago |  |  |  |  |
|  | Haiti | Q4 |  |  |  |
|  | Dominica |  | 1 | 1 | 1 |
|  | Dominican Republic | Q4 | 1 | 1 | 1 |
|  | St. Kitts and Nevis |  | 3 | 3 | 2 |
|  | St. Vincent and the Grenadines |  | 1 | 1 | 1 |
|  | Peru | Q4 | 1 | 1 | 1 |
|  | Mexico | Q4 | 1 | 1 | 1 |
|  | Guatemala |  | 1 | 1 | 1 |
|  | Bolivia | Q4 | 2 | 1 | 1 |
|  | Paraguay | Q4 | 1 | 1 | 2 |
|  | St. Lucia | Q3 | 2 | 2 | 2 |
|  | Ecuador | Q4 | 1 | 2 | 2 |
|  | Venezuela, RB | Q3 | 2 | 2 | 2 |
|  | Uruguay | Q3 | 1 | 1 | 1 |
|  | Suriname | Q4 | 2 | 2 | 2 |
|  | El Salvador | Q4 | 2 | 2 | 2 |
|  | Guyana | Q3 | 1 | 1 | 1 |
|  | Nicaragua | Q3 | 1 | 1 | 1 |
|  | Costa Rica | Q4 | 1 | 1 | 1 |
|  | Bahamas, The |  | 1 | 2 | 1 |
|  | Cuba |  | 2 | 2 | 2 |
|  | Colombia | Q4 |  |  |  |
|  | Barbados |  | 1 | 1 | 1 |
|  | Panama | Q4 | 1 | 1 | 1 |
|  | Argentina | Q3 | 3 | 3 | 3 |
|  | Cayman Islands |  | 2 | 2 | 2 |
|  | Antigua and Barbuda |  | 1 | 1 | 1 |
|  | Aruba |  |  |  |  |
|  | Belize | Q4 | 1 | 1 | 1 |
|  | Grenada |  | 1 | 1 | 1 |
|  | Chile | Q4 |  |  |  |
|  | Honduras | Q4 | 2 | 1 | 1 |
| North America | United States | Q3 |  |  |  |
|  | Canada | Q1 |  |  |  |
| Asia | Yemen, Rep. | Q2 | 1 | 1 | 1 |
|  | Israel | Q3 | 1 | 1 | 1 |
|  | India | Q2 | 1 | 1 | 1 |
|  | Cambodia | Q2 | 2 | 2 | 2 |
|  | Vietnam | Q2 | 1 | 1 | 1 |
|  | Indonesia | Q2 | 1 | 1 | 1 |
|  | Pakistan | Q1 |  |  |  |
|  | Iraq | Q1 | 3 | 2 | 3 |
|  | Libya |  | 1 | 1 | 1 |
|  | Nepal | Q1 | 1 | 1 | 1 |
|  | Syrian Arab Republic | Q2 | 3 | 3 | 3 |
|  | Uzbekistan | Q2 | 3 | 3 | 3 |
|  | Qatar | Q3 | 2 | 1 | 1 |
|  | Philippines | Q3 | 3 | 3 | 2 |
|  | Kuwait |  | 3 | 3 | 3 |
|  | Oman |  | 3 | 3 | 3 |
|  | Lebanon |  | 2 | 3 | 2 |
|  | Jordan | Q2 | 3 | 3 | 3 |
|  | Armenia | Q1 | 3 | 3 | 3 |
|  | Brunei Darussalam |  | 3 | 3 | 3 |
|  | Singapore | Q3 | 1 | 2 | 2 |
|  | Bahrain |  | 2 | 2 | 2 |
|  | Saudi Arabia |  | 3 | 3 | 3 |
|  | Tajikistan | Q1 | 3 | 2 | 2 |
|  | United Arab Emirates |  | 2 | 2 | 3 |
|  | Bangladesh | Q1 | 2 | 2 | 2 |
|  | Thailand | Q3 | 3 | 3 | 3 |
|  | Azerbaijan | Q2 | 2 | 2 | 2 |
|  | Hong Kong SAR, China | Q3 | 3 | 2 | 3 |
|  | Malaysia | Q4 | 3 | 3 | 3 |
|  | China | Q3 | 3 | 3 | 3 |
|  | Turkey | Q3 | 3 | 3 | 3 |
|  | Macau |  | 2 | 2 | 2 |
|  | Korea, Rep. | Q1 | 1 | 1 | 1 |
|  | Burma |  | 1 | 2 | 2 |
|  | Korea, Dem. Rep. |  | 2 | 3 | 3 |
|  | Timor-Leste |  |  |  |  |
|  | Mongolia | Q2 | 2 | 2 | 2 |
|  | Russian Federation | Q3 | 3 | 3 | 3 |
|  | West Bank and Gaza | Q2 | 2 | 2 | 2 |
|  | Japan | Q1 |  |  |  |
|  | Turkmenistan | Q3 | 3 | 3 | 3 |
|  | Maldives | Q2 | 3 | 3 | 3 |
|  | Afghanistan | Q1 | 1 | 1 | 1 |
|  | Lao PDR | Q2 |  |  |  |
|  | Sri Lanka | Q2 | 1 | 1 | 1 |
|  | Iran, Islamic Rep. | Q2 | 2 | 3 | 3 |
|  | Kyrgyz Republic | Q2 |  |  |  |
|  | Kazakhstan | Q1 | 1 | 1 | 1 |
|  | Bhutan | Q2 | 3 | 3 | 3 |
|  | Myanmar |  | 3 | 3 | 2 |
| Europe | Ireland | Q2 | 3 | 2 | 3 |
|  | Belgium | Q1 | 3 | 3 | 3 |
|  | Sweden | Q1 | 2 | 2 | 2 |
|  | Croatia | Q1 | 3 | 3 | 3 |
|  | Switzerland | Q1 | 3 | 2 | 2 |
|  | Montenegro | Q1 | 3 | 3 | 3 |
|  | Finland | Q1 |  |  |  |
|  | Norway | Q1 | 3 | 3 | 2 |
|  | Georgia | Q3 |  |  |  |
|  | Ukraine | Q1 | 2 | 2 | 2 |
|  | Germany | Q1 | 1 | 1 | 1 |
|  | Bosnia and Herzegovina | Q2 | 1 | 1 | 1 |
|  | Latvia | Q2 | 1 | 1 | 1 |
|  | Malta |  | 1 | 1 | 1 |
|  | Slovenia | Q1 | 1 | 1 | 1 |
|  | Austria | Q1 | 1 | 1 | 1 |
|  | Portugal | Q2 |  |  | 1 |
|  | Luxembourg | Q1 | 1 | 1 | 1 |
|  | United Kingdom | Q2 | 1 | 1 | 1 |
|  | Czech Republic | Q1 | 2 | 2 | 2 |
|  | Netherlands | Q1 | 2 | 1 | 2 |
|  | San Marino |  | 1 | 1 | 1 |
|  | Belarus | Q1 | 2 | 2 | 2 |
|  | Bulgaria | Q1 |  |  | 2 |
|  | Serbia | Q1 |  |  |  |
|  | Andorra |  | 1 | 1 | 1 |
|  | Estonia | Q2 | 1 | 1 | 1 |
|  | Hungary | Q1 | 1 | 1 | 1 |
|  | Iceland |  | 1 | 1 | 1 |
|  | Italy | Q2 | 1 | 1 | 1 |
|  | Poland | Q2 | 1 | 1 | 1 |
|  | Romania | Q1 | 1 | 1 | 1 |
|  | France | Q1 | 1 | 1 | 1 |
|  | Slovak Republic | Q1 | 1 | 1 | 1 |
|  | Moldova | Q1 | 1 | 1 | 1 |
|  | Denmark | Q1 | 3 | 3 | 3 |
|  | Albania | Q2 | 1 | 1 | 1 |
|  | Lithuania | Q2 |  |  | 1 |
|  | Cyprus |  | 1 | 1 | 1 |
|  | Monaco |  | 1 | 1 | 1 |
|  | Spain | Q2 | 1 | 1 | 1 |
|  | Macedonia, Q1YR | Q3 | 1 | 1 | 1 |
|  | Greece | Q2 | 1 | 1 | 1 |
| Oceania | Fiji | Q3 | 1 | 1 | 1 |
|  | Micronesia, Q1ed. Sts. | Q4 | 1 | 1 | 1 |
|  | Samoa |  | 1 | 1 | 1 |
|  | Tonga |  |  |  |  |
|  | Guam |  | 1 | 1 | 1 |
|  | Vanuatu |  | 2 | 2 | 2 |
|  | Solomon Islands |  | 1 | 1 | 1 |
|  | Kiribati |  | 2 | 2 | 2 |
|  | Palau |  |  |  |  |
|  | New Caledonia |  | 1 | 1 | 1 |
|  | American Samoa |  | 1 | 1 | 1 |
|  | French Polynesia |  | 1 | 1 | 1 |
|  | Marshall Islands |  | 1 | 1 | 1 |
|  | New Zealand |  |  |  |  |
|  | Australia |  | 2 | 2 | 2 |
|  | Northern Mariana Islands |  | 1 | 1 | 1 |
|  | Papua New Guinea |  | 2 | 2 | 2 |
|  | Tuvalu |  | 1 | 1 | 1 |
|  | Nauru |  | 1 | 1 | 1 |
